# An iridium(iii)-based photosensitizer disrupting the mitochondrial respiratory chain induces ferritinophagy-mediated immunogenic cell death†

**DOI:** 10.1039/d4sc01214c

**Published:** 2024-04-02

**Authors:** Tao Feng, Zixin Tang, Johannes Karges, Jun Shu, Kai Xiong, Chengzhi Jin, Yu Chen, Gilles Gasser, Liangnian Ji, Hui Chao

**Affiliations:** a MOE Key Laboratory of Bioinorganic and Synthetic Chemistry, State Key Laboratory of Anti-Infective Drug Discovery and Development, Guangdong Basic Research Center of Excellence for Functional Molecular Engineering, School of Chemistry, Guangdong Provincial Key Laboratory of Digestive Cancer Research, The Seventh Affiliated Hospital, Sun Yat-Sen University Guangzhou 510006 P. R. China ceschh@mail.sysu.edu.cn; b Faculty of Chemistry and Biochemistry, Ruhr University Bochum Universitätsstrasse 150 44780 Bochum Germany; c Chimie ParisTech, PSL University, CNRS, Institute of Chemistry for Life and Health Sciences, Laboratory for Inorganic Chemical Biology 75005 Paris France gilles.gasser@chimieparistech.psl.eu; d MOE Key Laboratory of Theoretical Organic Chemistry and Functional Molecule, School of Chemistry and Chemical Engineering, Hunan University of Science and Technology Xiangtan 400201 P. R. China

## Abstract

Cancer cells have a strategically optimized metabolism and tumor microenvironment for rapid proliferation and growth. Increasing research efforts have been focused on developing therapeutic agents that specifically target the metabolism of cancer cells. In this work, we prepared 1-methyl-4-phenylpyridinium-functionalized Ir(iii) complexes that selectively localize in the mitochondria and generate singlet oxygen and superoxide anion radicals upon two-photon irradiation. The generation of this oxidative stress leads to the disruption of the mitochondrial respiratory chain and therefore the disturbance of mitochondrial oxidative phosphorylation and glycolysis metabolisms, triggering cell death by combining immunogenic cell death and ferritinophagy. To the best of our knowledge, this latter is reported for the first time in the context of photodynamic therapy (PDT). To provide cancer selectivity, the best compound of this work was encapsulated within exosomes to form tumor-targeted nanoparticles. Treatment of the primary tumor of mice with two-photon irradiation (720 nm) 24 h after injection of the nanoparticles in the tail vein stops the primary tumor progression and almost completely inhibits the growth of distant tumors that were not irradiated. Our compound is a promising photosensitizer that efficiently disrupts the mitochondrial respiratory chain and induces ferritinophagy-mediated long-term immunotherapy.

## Abstract 2

A mitochondria-localizing iridium(iii) photosensitizer produces singlet oxygen and superoxide anion radicals upon two-photon irradiation, disrupting the mitochondrial respiratory chain and inducing ferritinophagy and immunogenic cell death.

## Graphical abstract

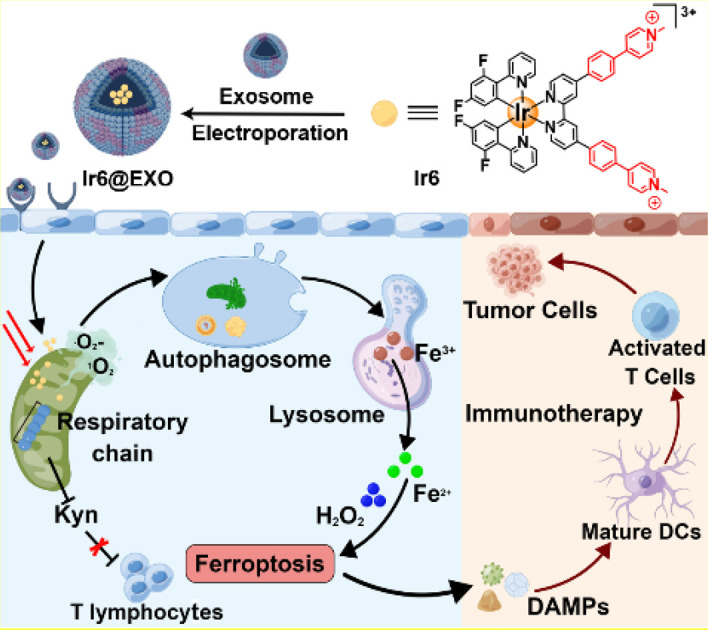

## Introduction

Melanoma is amongst the most aggressive of all skin cancers.^1,2^ As the treatment of melanoma tumors remains challenging in clinics due to elevated rates of drug resistance and rapid metastasis formation, there is a need for the development of new therapeutic mechanisms and anticancer strategies.^1,3^ Metabolic adaptability plays a crucial role in the proliferation and viability of cancer cells and represents a significant factor in the efficacy of anticancer treatments.^4–6^ During tumor genesis and development processes, cancer cells strategically choose metabolic alterations that optimize the tumor microenvironment, thereby promoting cancer cell survival and proliferation.^7,8^ As one of the crucial subcellular organelles, the mitochondria are involved in many biological processes, including energy production, metabolic regulation, immune responses, and programmed cell death.^9–12^ A reduction or imbalance in the regulation of mitochondrial homeostasis can disturb the intracellular environment and ultimately result in the death of cancer cells.^13^ For these reasons, increasing research efforts have been focused on new anticancer strategies that specifically target mitochondria.

Various physiological processes inside cells and the mitochondria are controlled through the signaling properties of reactive oxygen species (ROS) that are produced during the mitochondria respiratory chain (MRC).^14,15^ A malfunctioning of the MRC can lead to electron leakage and uncontrolled ROS generation.^16,17^ This excess in ROS induces oxidative stress inside the cells, ultimately resulting in unregulated cell death.^18–20^ Recent studies have reported on a rarely described form of cell death called ferritinophagy that combines cell death characteristics of autophagy and ferroptosis.^21,22^ During this type of cell death, the expression of the nuclear receptor coactivator 4 (NCOA4) protein is strongly up-regulated. As NCOA4 functions as a selective cargo receptor for ferritin, the autophagic degradation of ferritin is caused. The decomposition of ferritin results in the release of free iron ions that can catalytically produce highly cytotoxic hydroxyl radicals (˙OH) *via* the Fenton reaction, ultimately resulting in cell death.^23^ To date, compounds that disrupt the mitochondrial respiratory chain and trigger ferritinophagy are scarce.

**Fig. 1:**
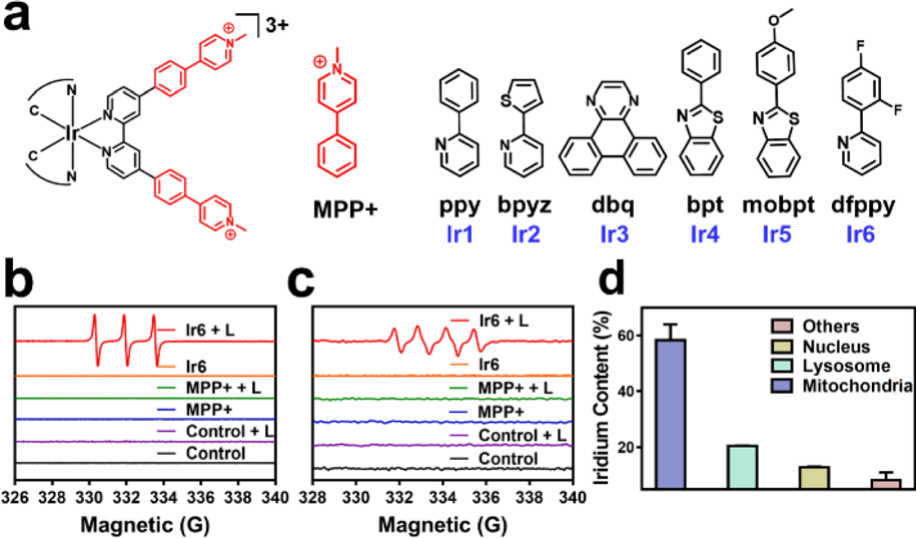
(a) Chemical structures of the Ir(iii) photosensitizers Ir1–Ir6 and MMP+. (b) Electron spin resonance spectra of Ir6 and MPP+ upon incubation with the ^1^O_2_-specific scavenger 2,2,6,6-tetramethylpiperidine in the dark or upon light irradiation (L). (c) Electron spin resonance spectra of Ir6 and MPP+ upon incubation with the scavenger 5,5-dimethyl-1-pyrroline-*N*-oxide in the dark or upon light irradiation (L). (d). The subcellular distribution of A375 cells upon incubation with Ir6 (10 μM) for 6 h to determine the metal content inside the major organelles. L = two-photon laser irradiation at 720 nm, 40 mW, 120 s.

The neurotoxin 1-methyl-4-phenylpyridinium (MPP+, Fig. 1) has been demonstrated to induce oxidative stress in neuronal cells and disturb the MRC's activity, ultimately resulting in the death of dopaminergic neurons.^24,25^ At high concentrations (>1 mM), the compound has been shown to induce ferroptosis in PC12 cells and apoptosis in SK-N-SH or CHP 212 cells through the breaking of the respiratory chain of mitochondria.^26,27^ Over the last years, various types of mitochondria-targeting therapeutic metal complexes (*i.e.*, polypyridine-based metal complexes, cyclometalated complexes, or triphenylphosphine-based metal complexes) have been developed.^28–32^ Among the most promising classes of compounds, cyclometalated Ir(iii) complexes have received increased attention due to their high physiological stability, biocompatibility, and attractive pharmacokinetic properties. Based on their rich photophysical properties (*i.e.*, strong luminescence, significant Stokes shift, high ROS production) that can be fine-tuned through the choice of the ligand environment, cyclometalated Ir(iii) complexes are widely studied as photosensitizers for photodynamic therapy (PDT).^33–35^ Despite these promising properties, most of these photosensitizers are excited with ultraviolet or blue light, limiting their applications *in vivo*.^33,34,36^ To enable the treatment of deep-seated or large tumors, compounds with excitation in the near-infrared region are required. A promising approach to shift the excitation into the desired near-infrared region is the application of two-photon irradiation. Ir(iii) complexes have attracted much attention for this purpose.^37–40^

**Scheme 1:**
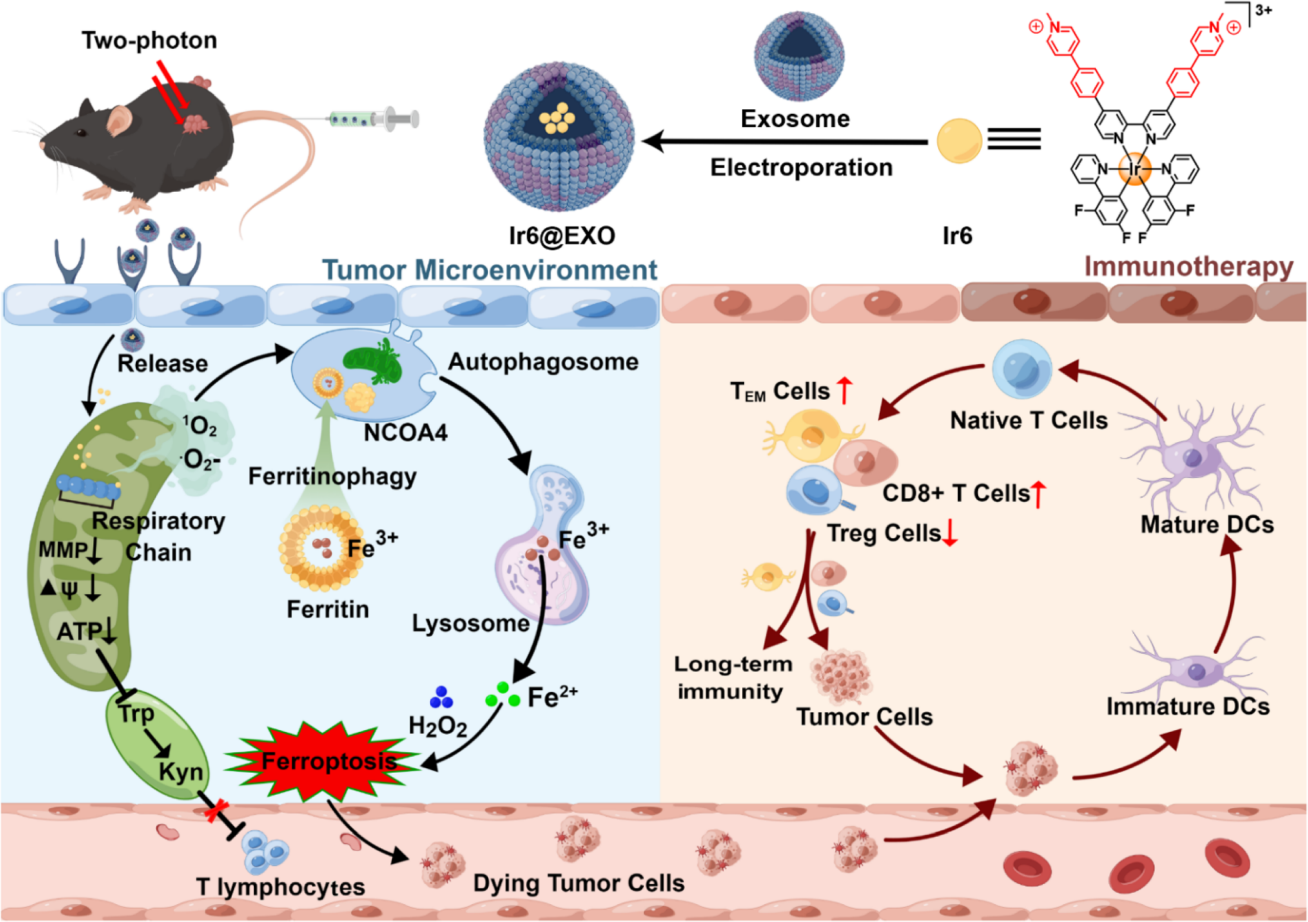
Schematic view of the encapsulation within exosomes of Ir6 that induces mitochondrial ROS production upon two-photon irradiation, leading to the disruption of the mitochondrial respiratory chain and the induction of ferritinophagy-mediated immunogenic cell death.

Herein, the encapsulation of ferritinophagy and immunogenic cell death-inducing cyclometalated Ir(iii) photosensitizers within exosomes for tumor-targeted photodynamic immunotherapy is presented (Scheme 1). A series of 1-methyl-4-phenylpyridinium functionalized Ir(iii) complexes with various ancillary ligands were first synthesized and photophysically evaluated. The biological properties of the most promising derivative, namely Ir6, were then studied in-depth against human malignant melanoma cells, which were found to be the most sensitive cells tested in this work with this compound upon light irradiation. This compound was found to selectively accumulate in the mitochondria and generate a mixture of singlet oxygen and superoxide anion radicals upon two-photon irradiation in this organelle, leading to the disruption of the MRC and triggering cell death by ferritinophagy and immunogenic cell death. The metal complex was encapsulated within exosomes to enhance the pharmacological properties and provide cancer cell selectivity to form tumor-targeted nanoparticles. Upon treatment of the primary tumor with two-photon irradiation (720 nm) 24 h after injection of the nanoparticles in the tail vein of mice, both the primary tumor and the distant secondary human melanoma tumor that was not irradiated were nearly eradicated due to the strong anticancer immune response.

## Results and discussion

### Synthesis and characterization

In this work, cyclometalated Ir(iii) complexes with an MPP+ functionalized bipyridine ligand and various ancillary ligands were synthesized (Ir1–Ir6, Fig. 1a and Scheme S1†). The compounds were characterized by nuclear magnetic resonance spectroscopy and high-resolution electrospray ionization mass spectrometry (Fig. S1–S12†). The purity of the compounds was demonstrated by elemental analysis and HPLC. All complexes have a purity greater than 95% (Fig. S14d–i and Table S1†). Fourier-transform infrared spectroscopy of Ir1–Ir6 showed a low-frequency band at approximately 2920 cm^−1^ and 1460 cm^−1^ corresponding to the CH_3_ vibration, 800–860 cm^−1^ and 680–725 cm^−1^ corresponding to the substituted benzene vibration in complexes (Fig. S13†). Overall, these results indicate the identity and purity of the metal complexes. Ir1–Ir6 showed firm absorption peaks in the ultraviolet and blue regions (Fig. S14a†). Ir1–Ir2 were found with a negligible amount of emission. Ir3–Ir6 exhibited an intense phosphorescence in the red region upon excitation at 405 nm. Ir6 had the most intense emission of the tested series of metal complexes (Fig. S14b†). Based on the high positive charge of the metal complexes, these are considered to be hydrophilic. This was confirmed by determining their partition coefficients between octanol and water (Fig. S14c†). HPLC checked the purity of complexes, and all complexes were greater than 95% (Fig. S14d–i and Table S1†). As shown in Fig. S15,†Ir6 has the highest fluorescence quantum yield in DMSO with 20.27%. The two-photon absorption properties of the metal complexes were measured using the femtosecond luminescence measurement method. The metal complexes showed a two-photon absorption from 700 to 800 nm. Ir6 had the most substantial two-photon absorption of the tested series, with a two-photon absorption cross-section of 128 GM at 720 nm (Fig. S16†). The ^1^O_2_-specific probe 9,10-bis(bromomethyl)anthracene was used to investigate the metal complexes' ability to produce singlet oxygen (^1^O_2_). All tested metal complexes were found to generate ^1^O_2_ upon irradiation at 405 nm (Fig. S17†). Ir6 was demonstrated to produce ^1^O_2_ with a quantum yield of 0.77 (Table S2†). Based on its solid photophysical properties (two-photon absorption, emission, and ROS production), further studies were performed with Ir6.

The absorption spectra of the ^1^O_2_-specific probe 9,10-bis(bromomethyl)anthracene probe demonstrated a time-dependent drastic drop in absorption upon treatment with Ir6 and two-photon irradiation, indicative of the solid ^1^O_2_ production (Fig. S18†). The ability to produce ^1^O_2_ was further verified by electron spin resonance spectroscopy upon incubation with the ^1^O_2_-specific scavenger 2,2,6,6-tetramethylpiperidine. While no signal was observed in the dark, the characteristic signal (peak integral ratio 1 : 1 : 1) was monitored after light irradiation (Fig. 1b). The ability to generate other types of ROS was studied upon incubation with the scavenger 5,5-dimethyl-1-pyrroline-*N*-oxide. The spectra showed the formation of the characteristic peak pattern (peak integral ratio 1 : 1 : 1 : 1) for the generation of superoxide anion radicals (˙O_2_^−^) upon two-photon irradiation (Fig. 1c). We note that ˙OH is not formed during this process, as demonstrated by ESR and UV-Vis spectroscopy, as shown in the ESI.† However, almost no hydrogen peroxide was detected by electron spin resonance spectroscopy and UV-Vis (Fig. S19†). Of high importance, the stability of the compound under physiological conditions was studied upon incubation in DMEM containing fetal bovine serum. No changes in the absorption spectrum of Ir6 was observed in HPLC and UV-Vis (Fig. S20†). In addition, the photostability of Ir6 in methanol was assessed upon continuous irradiation with a two-photon laser at 720 nm. No changes in the nuclear magnetic resonance spectra were observed, indicative of the high photostability of Ir6 (Fig. S21†).

### Localization and photo-cytotoxicity

One- and two-photon excited confocal laser scanning microscopy (CLSM) images showed an intense phosphorescence of Ir6 inside A375 cells (Fig. S22†). Upon co-incubation of Ir6 with commercially available cell organelle trackers, the subcellular localization inside the cancer cells was determined by CLSM. A preferential accumulation inside the mitochondria was observed (Fig. S23†). Inductively coupled plasma mass spectrometry (ICP-MS) was used as a complementary method to quantify the metal content in different organelles. Ir6 preferentially accumulated in the mitochondria (Fig. 1d). To investigate the time-dependent accumulation of Ir6 in the mitochondria, this cell organelle was extracted after various time points using a commercially available kit, and the distribution was quantified by ICP-MS analysis. Ir6 rapidly accumulated in the mitochondria, reaching a maximum after approximately 6 h of incubation (Fig. S24†). The cytotoxicity of MPP+ and Ir6 against various cells was evaluated in the dark and upon exposure to two-photon irradiation (720 nm, 40 mW, 120 s) (Table S3†). MPP+ was found to be non-toxic up to high concentrations in the dark and upon light exposure (IC_50_ > 500 μM). Ir6 showed cytotoxicity in the high micromolar range in the dark (IC_50_, dark = 71.02–89.35 μM) and a phototoxic effect upon two-photon irradiation in the low micromolar range (IC_50_, light = 4.26–12.75 μM). Due to its highest therapeutic effect against human malignant melanoma cells, this cell line was used to study its biological mechanism of action. The therapeutic effect of the treatment was visualized by fluorescence microscopy using the cell live/dead stain calcein-AM/EthD-1. While the cells incubated with MPP+ or Ir6 in the dark were found to be alive, the cells treated with Ir6 and exposed to light were dead, confirming the phototoxicity of the photosensitizer and not MMP+ (Fig. S25†).

### Mitochondrial respiration chain disruption

**Fig. 2:**
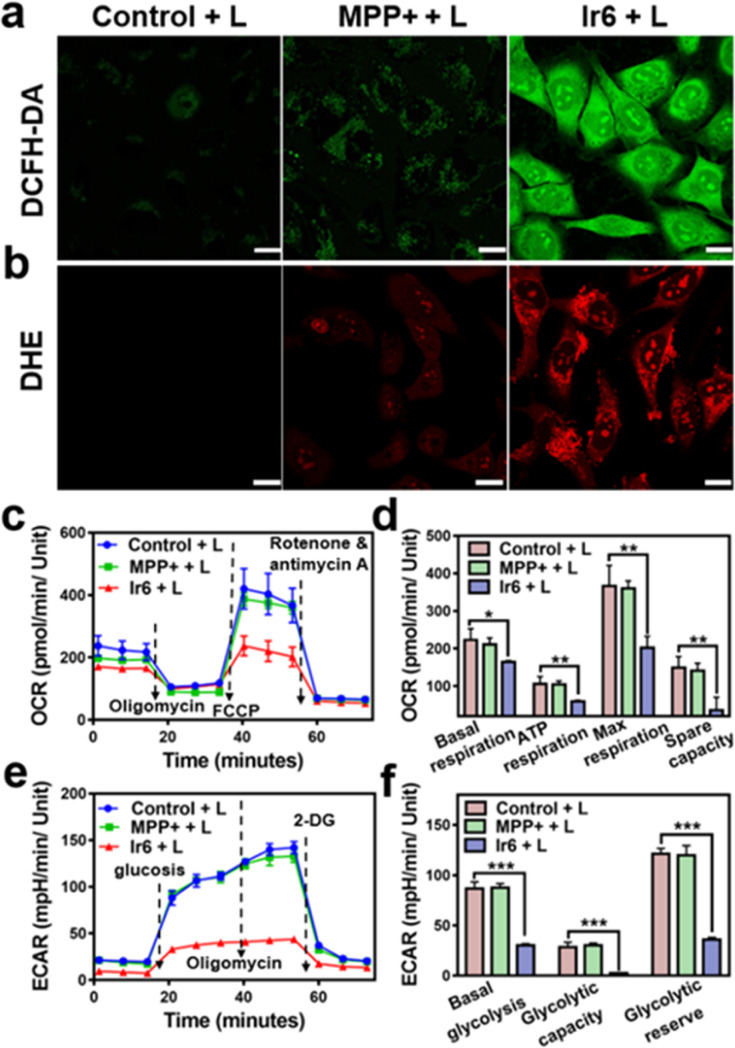
ROS production, modulation of oxidative phosphorylation, and glycolysis metabolism upon treatment of A375 cells with Ir6 and MPP+ for 6 h. (a) Fluorescence microscopy images of A375 cells incubated with the ROS-specific probe 2,7-dichlorofluorescein diacetate and treated with Ir6 or MPP+ upon light irradiation (L). (b) Fluorescence microscopy images of A375 cells incubated with the ˙O_2_^−^-specific probe dihydroethidium and treated with Ir6 or MPP+ upon irradiation (L). (c) Kinetic profile and (d) quantification of the oxygen consumption rate (OCR) of A375 cells treated with Ir6 and MPP+ upon light irradiation (L). (e) Kinetic profile and (f) quantification of the extracellular acidification rate (ECAR) profile of A375 cells treated with Ir6 and MPP+ upon irradiation (L). L = two-photon laser irradiation at 720 nm, 40 mW, 120 s, concentration: Ir6 (10 μM), MPP+ (100 μM). Scale bar = 20 μm. *n* = 3. *p < 0.05, **p < 0.01, ***p < 0.001.

For an understating of the ability of the metal complex to generate ROS inside the cell, the cancer cells were co-incubated with the ROS-specific probe 2,7-dichlorodihydrofluorescein diacetate or ˙O_2_^−^-specific probe dihydroethidium. While no formation of ROS was observed during the incubation with MPP+ or Ir6 in the dark (Fig. S26†), strong fluorescence of the ROS-specific probe (Fig. 2a) and the ˙O_2_^−^-specific probe (Fig. 2b) were monitored upon treatment with Ir6 and exposure to two-photon irradiation. These results were further verified by flow cytometry (Fig. S27†). The generation of ROS inside the mitochondria could lead to mitochondrial dysfunction, a decrease in mitochondrial membrane potential, and the inhibition of the respiration chain. Changes in the mitochondrial membrane potential were monitored by flow cytometry using the specific dye JC-1. The plots demonstrated the loss of the mitochondrial membrane potential upon treatment with Ir6 and exposure to light (Fig. S28†). To investigate the inhibition of the respiration chain, the levels of oxidative phosphorylation in A375 cells were evaluated by measuring the oxygen consumption rate upon treatment. The cancer cells treated with Ir6 and exposure to irradiation exhibited a significant reduction in basal respiration, ATP production, maximal respiration, and spare respiration (Fig. 2c, d, S29a and b†), all indicative of the strong suppression of oxidative phosphorylation during treatment. The changes in the glycolysis metabolism were studied upon determination of the extracellular acidification rate during treatment. The cancer cells treated with Ir6 and exposure to irradiation showed 34% basal glycolysis, 8% glycolytic capacity, and 29% cellular glycolytic reserve (Fig. 2e, f, S29c and d†), suggestive of severe glycolysis disruption during treatment. Previous studies have indicated that treating cancer cells with MPP+ at high concentrations and long incubation times could influence mitochondrial respiration. However, this effect diminished when low concentrations or short incubation times were used. Our results indicate a short-lived and reversible effect of MPP+.^41,42^ For a comparable effect to the evaluation of the MRC disruption in this work, cancer cells were treated with Ir6 for 6 h, the cells were washed to remove any non-internalized compounds, and then irradiated with the light source. Under these conditions, MPP+ did not influence the MRC. In contrast, Ir6 demonstrated the ability to disrupt mitochondrial oxidative phosphorylation and glycolysis processes. The dysregulation of the cancer cells' energy supply was assessed upon determining the intracellular levels of ATP. The treatment of Ir6 with light was found to drastically reduce the ATP levels to 40% (Fig. S30a†) compared to cancer cells treated in the dark (Fig. S30b†). Consequently, the treatment also downregulated the levels of NADH (Fig. S30c†) and inhibited the conversion of tryptophan to kynurenine (Fig. S30d†). Previous studies have suggested that the conversion of tryptophan to kynurenine is downregulated in cancer cells, which can evade immune responses and are associated with a strong tumor progression.^43–45^ Therefore, the therapeutic intervention of Ir6 may support the organism's immunogenic response and enhance the therapeutic effects of immunotherapies. These results indicate that Ir6 can selectively accumulate in the mitochondria, cause oxidative stress in this organelle upon light irradiation, and ultimately trigger the disruption of mitochondrial oxidative phosphorylation and glycolysis metabolisms.

### Cell death mechanism

**Fig. 3:**
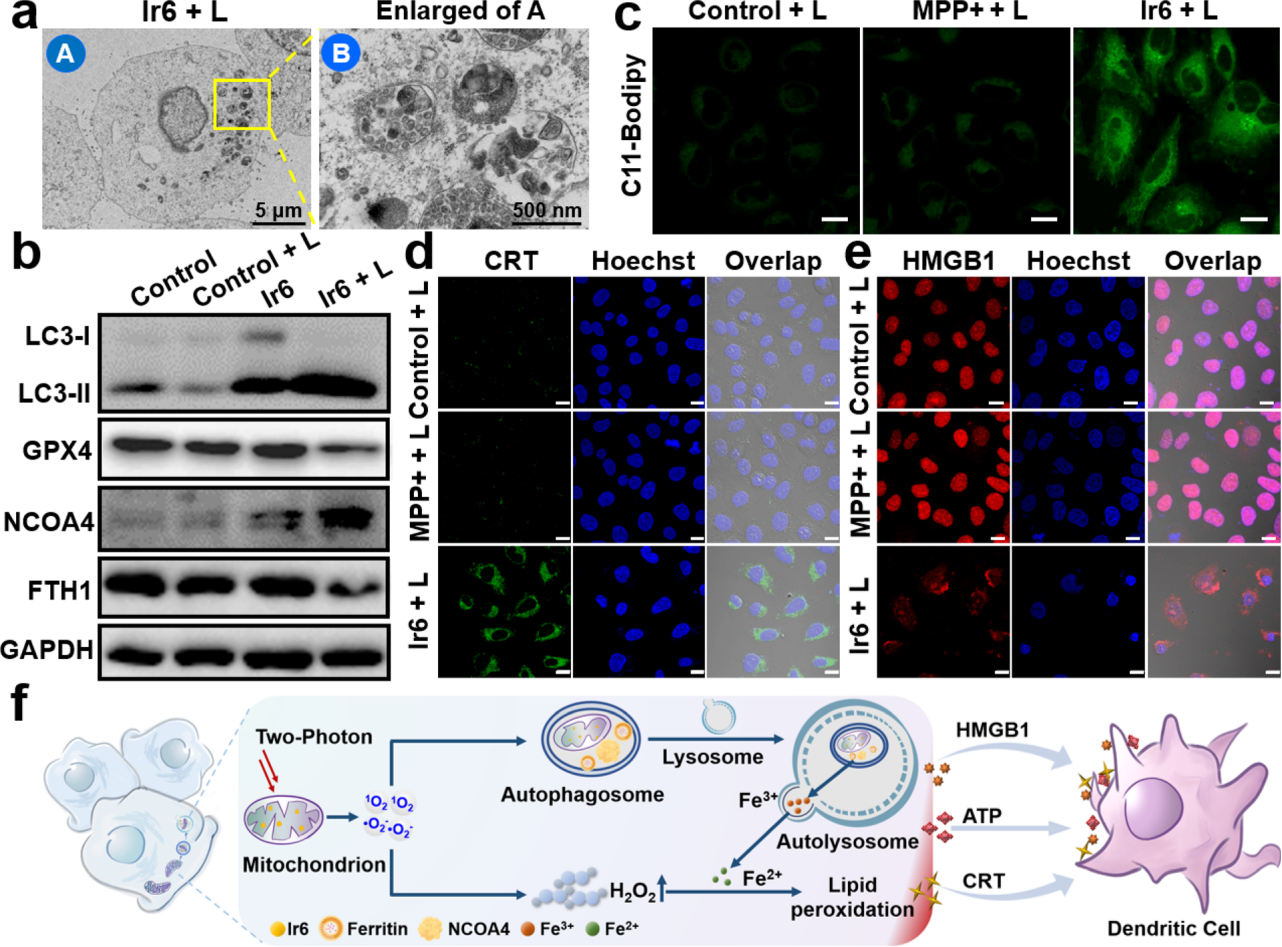
Cell death mechanism of Ir6 in A375 cells. (a) Representative transmission electron microscopy image of the autophagosomes of A375 cells upon treatment with Ir6 upon irradiation (L). (b) Western blot analysis of the expression level of LC3-I, LC3-II, GPX4, NCOA4, and FTH1 of A375 cells upon treatment with Ir6 in the dark or irradiation (L). (c) CLSM of A375 cells incubated with the lipid peroxide-specific probe C11-Bodipy and treated with MPP+ or Ir6 upon irradiation (L). Scale bar = 20 μm. Immunofluorescence CLSM of A375 cells incubated with (d) CRT-specific antibody and (e) HMGB1 protein-specific antibody and treated with MPP+ or Ir6 upon irradiation (L). Scale bar = 20 μm. (f) Proposed cell death mechanism of ferritinophagy and immunogenic cell death by Ir6 upon irradiation. Concentration: Ir6 (10 μM), MPP+ (100 μM). L = two-photon laser irradiation at 720 nm, 40 mW, 120 s.

To investigate the cell death mechanism of the treatment, cancer cells were treated with apoptosis (z-VAD-fmk), necrosis (necrostatin-1), autophagy (3-methyladenine), ferroptosis (ferrostatin-1 and deferoxamine), and pyroptosis (disulfiram) inhibitors before being incubated with Ir6 and irradiated by light. The treatment with apoptosis, necrosis, and pyroptosis inhibitors did not influence cell survival. In contrast, treatment with autophagy and ferroptosis inhibitors strongly augmented cell survival, indicating that these mechanisms are primarily responsible for cell death (Fig. S31†). Following this preliminary insight, the biological effects of Ir6 on these cell death mechanisms were further studied. Using transmission electron microscopy, changes in the cell morphology were monitored upon treatment with Ir6 and exposure to light irradiation. Microcopy images showed that the metal complex induced the formation of autophagosomes upon exposure to light irradiation (Fig. 3a and S32†). Western blot analysis showed the overexpression of LC3-II upon treatment with Ir6 and exposure to light irradiation (Fig. 3b, S33 and S34†), facilitating the proteolytic cleavage/lipidation and induction of autophagic processes. These results indicate that cell death is partly attributed to autophagy. To investigate ferroptotic cell death processes, the marker ferroptosis protein glutathione peroxidase 4 (GPX4) is downregulated in intracellular levels upon treatment with Ir6 and exposure to light irradiation (Fig. 3b, S33 and S34†). The lipid peroxidation during the treatment with the metal complex was further studied using the lipid peroxide-specific probe C11-Bodipy. Microscopy images suggest that treating Ir6 with light-induced the formation of lipid peroxides inside the cancer cells (Fig. 3c). These results indicate that the cell death is partly attributed to ferroptosis. NCOA4, which serves as the primary regulator of ferritinophagy, disrupts iron homeostasis and releases iron ions, thereby promoting the production of ROS through the Fenton reaction. Western blot analysis showed that the treatment with Ir6 and exposure to light triggered the overexpression of NCOA4 (Fig. 3b, S33 and S34†). As NCOA4 serves as a selective cargo receptor for ferritin recruitment in autophagosomes, the ferritin-heavy polypeptide 1 (FTH1) expression was investigated.^21,22,46^ The levels of the FTH1 were found to be reduced upon treatment with Ir6 and exposure to light (Fig. 3b, S33 and S34†). NCOA4 recruits ferritin and delivers it for lysosomal degradation, releasing free Fe(ii) ions.

To assess the intracellular concentration of Fe(ii), the cancer cells were incubated with the Fe(ii)-specific probe FerroOrange. While minimal fluorescence background was observed inside cells treated with MPP+ or Ir6 in the dark, a robust red emission was monitored upon treatment with Ir6 and exposure to light (Fig. S35†), indicative of free Fe(ii) ions. Besides the generation of ^1^O_2_, the metal complex can produce ˙O_2_^−^ upon irradiation that could disproportionate to form hydrogen peroxide (H_2_O_2_) and could catalytically be converted by the free Fe(ii) ions inside the cancer cells into highly cytotoxic ˙OH *via* the Fenton reaction. Using an H_2_O_2_-specific fluorescent probe, the formation of H_2_O_2_ upon treatment with Ir6 and exposure to light was verified (Fig. S36†). The generation of ˙OH upon treatment with Ir6 and exposure to light was confirmed using a ˙OH-specific fluorescent probe (Fig. S37†). These results suggest that Ir6 triggers cell death in cancer cells by a combined mechanism of autophagy and ferroptosis, referred to as ferritinophagy (Fig. 3f). We note that under our experimental conditions, we could not detect H_2_O_2_ in the blank group, which may be due to the low H_2_O_2_ content in the cells, the sensitivity of the H_2_O_2_ probe, or the gain value adjusted together with the laser intensity and other factors.

### *In vitro* immunotherapy

Recent studies have indicated that ferroptosis and downregulation of kynurenine could enhance the therapeutic effectiveness of cancer immunotherapy.^4,17^ Subsequently, the ability of the metal complex to induce immunogenic cell death was studied by monitoring immunogenic cell death hallmarks. The translocation of calreticulin (CRT) from the endoplasmic reticulum to the cell membrane upon treatment with Ir6 and exposure to light irradiation was observed by immunofluorescence CLSM (Fig. 3d and S38a†) and flow cytometry (Fig. S38c†), supporting the interaction of macrophages for tumor antigen presentation. The migration of human nuclear high mobility group box 1 (HMGB1) protein from the nucleus to the cytoplasm upon treatment with Ir6 and exposure to light was monitored by immunofluorescence CLSM (Fig. 3e and S38b†) and an enzyme-linked immunosorbent assay (Fig. S38d†), triggering the myeloid differentiation primary response signaling cascade necessary for antigen processing and presentation to T-cells. The release of ATP from the cytoplasm into the extracellular space was observed upon treatment with Ir6 and exposure to light (Fig. S38e†), promoting the attraction of tumor-specific cytotoxic T-cells. Using immunofluorescence CLSM, the 70 kD heat shock protein (HSP70) overexpression upon treatment with Ir6 and exposure to light irradiation was observed (Fig. S39†). Complementary, the ability of the metal complex to induce immunogenic cell death in the murine analog cell line B16–F10 was investigated (CLSM translocation of CRT: Fig. S40,† CLSM migration of HMGB1 protein: Fig. S41,† flow cytometry translocation of CRT: Fig. S42a,† enzyme-linked immunosorbent assay migration of HMGB1 protein: Fig. S42b,† release of ATP: Fig. S42c,† overexpression of HSP70: Fig. S43†). The combination of these results indicates the ability of Ir6 to efficiently induce immunogenic cell death upon light irradiation (Fig. 3f). To investigate whether the immunity activation is related to the cGAS-STING pathway, A375 cells treated with MPP+ or Ir6 were stained with PicoGreen to visualize the DNA release from the mitochondria. Microscopy images show that the mitochondria were swollen upon treatment with Ir6 and light irradiation, but no release of DNA was observed (Fig. S44†). Western blot analysis demonstrated no changes in the expression level of STING and P-STING (Fig. S45†), indicating that the immune activation does not primarily stem from the cGAS-STING pathway.

### Exosomes encapsulation

Nanoparticles are frequently modified with targeted antibodies, peptides, or other biomolecules to enhance the specific delivery of anticancer drugs to tumors.^47,48^ Nevertheless, including targeted ligands can occasionally impede the delivery of nanoparticles by increasing immune elimination.^49^ Exosomes are extracellular vesicles measuring approximately 40–150 nm in diameter and released by various human cell types. They have recently gained recognition as a promising drug delivery system.^50^ This is attributed to their exceptional biocompatibility, minimal toxicity, and remarkable encapsulation capacity.^51,52^ Significantly, exosomes exhibit a “homing” effect that can be effectively harnessed for the targeted delivery of therapeutic agents to tumors.^53^ Therefore, to enhance the pharmacological properties and provide a cancer-selective delivery of the therapeutic agent, Ir6 was encapsulated with exosomes (EXOs). A375-derived EXOs with an approximate diameter of 75 nm and a polydispersity index of 0.23 (Fig. S46†) were isolated using the ultracentrifugation method as previously described.^54^ Through electroporation, the metal complex was entrapped in the supramolecular structure, followingly referred to as Ir6@EXO-A, resulting in an approximate diameter of 95 nm and a polydispersity index of 0.27 (Fig. S47a†). Transmission electron microscopy images suggested no significant changes in the morphology of the EXOs after the encapsulation (Fig. S47b†). Using CLSM, the successful encapsulation of Ir6 was confirmed through the co-localization of the luminescence of Ir6 and the membrane probe 1,1′-dioctadecyl-3,3,3′,3′-tetramethylindocarbocyanine perchlorate (Fig. S47c†). Zeta potential measurements indicated a change from −38 mV for EXO to −20 mV for Ir6@EXO-A (Fig. S48a†). The stability of the nanoformulation was studied by monitoring their size over five days. As no significant changes were observed upon incubation in PBS (Fig. S48b†), the stability of Ir6@EXO-A under physiological conditions is confirmed. Western blot determined the levels of the marker proteins CD63 and CD9 of the EXOs. No significant changes were observed (Fig. S49 and S59†), suggesting that the incorporation of the metal complex did not drastically change the biological properties of the EXOs. Overall, these findings indicate the successful encapsulation of the photosensitizer into Ir6@EXO-A. (Photo-)toxicity experiments using the MTT assay showed that the encapsulation of Ir6 by exosomes did not affect its toxicity (Fig. S50†).

### Homologous targeting

To study the ability of the EXOs to selectively accumulate in A375 cells, cancerous A375 cells and non-cancerous human lung fibroblasts (HLF) cells were mixed. Ir6 or Ir6@EXO-A were incubated with the cell mixture, and CLSM analyzed the accumulation inside the cells. Ir6 was found to accumulate in A375 and HLF cells non-selectively. In contrast, Ir6@EXO-A was not detected in the HLF cells but was highly taken up by the A375 cells (Fig. S51†). These findings suggest the A375 tumor-selective targeting of Ir6@EXO-A.

### 3D multicellular tumor spheroids

After evaluation in a two-dimensional monolayer cell model, the biological properties of the metal complex were further studied in three-dimensional large multicellular tumor spheroids that can mimic the pathological conditions of solid tumors, such as proliferation gradients, a hypoxic center, or a three-dimensional cellular structure. Thus, A375 multicellular tumor spheroids with an approximate diameter of 900 μm were grown and used to evaluate the therapeutic properties of Ir6 and Ir6@EXO-A. Using z-stack one- and two-photon CLSM, the penetration of the multicellular tumor spheroid was studied. A luminescence signal at every section depth of Ir6@EXO-A (Fig. S52†) and Ir6 (Fig. S53†) was monitored, suggesting the complete penetration of the compounds into the cellular structure.

### *In vivo* therapeutic

**Fig. 4:**
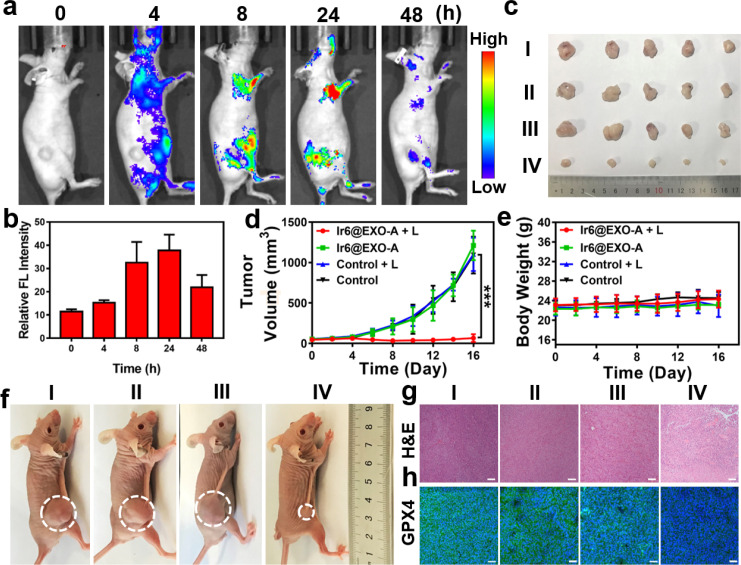
(a) Time-dependent luminescence photographs of an A375 tumor-bearing mouse model after injection of Ir6@EXO-A into the tail vein in an animal imaging system. (b) Determination of the time-dependent fluorescence in the tumor, *n* = 3. (c) Photographs of the tumor of an A375 tumor-bearing mouse model 16 days after treatment. (d) Tumor growth inhibition curves of an A375 tumor-bearing mouse model during the treatment. (e) Changes in the body weight of an A375 tumor-bearing mouse model during the treatment. (f) Representative photos of the xenograft model of A375 tumors after different treatments. (g) Hematoxylin and eosin stain (H&E) of the tumorous tissue of an A375 tumor-bearing mouse model 16 days after treatment. (h) Glutathione peroxidase 4 stain (GPX4) of the tumorous tissue of an A375 tumor-bearing mouse model 16 days after treatment. I: control, II: control + L, III: Ir6@EXO-A, IV: Ir6@EXO-A + L. L = two-photon laser irradiation at 720 nm, 50 mW, 300 s. Scale bar = 50 μm. Error bars = standard deviation, *n* = 5. ***p < 0.001.

Clinical trials have demonstrated that exosomes have emerged as promising drug delivery vehicles due to their ability to mitigate the toxic effects associated with the introduction of foreign substances into the body.^55,56^ Notably, these findings extend to other engineered cell types, as no significant toxicity or immune response was observed.^57,58^ Studies have found that homologous exosomes rarely cause adverse immune responses in the blood circulation.^59,60^ These results highlight the minimal toxicity exhibited by exosomes while concurrently yielding clinical advantages. Based on our promising findings, the therapeutic properties were further studied in a mouse model. As a crucial requirement for an animal experiment, the biosafety of the nanoparticles was assessed upon injection into the tail vein of healthy BALB/c nude mice. No signs of pain, stress, or discomfort were observed. After seven days, blood samples were collected for a biochemical screening that assessed alkaline phosphatase, alanine aminotransferase, creatinine levels, and aspartate aminotransferase. Encouragingly, no deviations from normal levels were observed for any of the biochemical factors (Fig. S54, Table S4†), indicating the high biocompatibility of Ir6@EXO-A. Time-dependent monitoring of the metal concentration in the blood by ICP-MS determined the lifetime of blood circulation. Ir6@EXO-A exhibited a blood circulation half-life of 3.4 h (Fig. S55a†). The excretion of the compound was studied by monitoring the metal content in the urine and feces by ICP-MS. The data indicated that Ir6@EXO-A was nearly eliminated from the animal after 96 h through fecal excretion (Fig. S55b†). The compound's biodistribution in an A375 tumor-bearing mouse model was studied using an animal imaging system based on the metal complex's strong emissive properties. Several hours after injection of the nanoparticles in the tail vein, they are distributed in the body of the animal model (Fig. 4a). The time-dependent monitoring of the emission signal of the nanoparticles suggests that the nanomaterial reached its maximal tumor accumulation approximately 24 h after injection (Fig. 4b). Complementary to this, the organs were collected, and the luminescence intensity is shown in Fig. S56a and b.†Ir6@EXO-A was found to accumulate in the tumor with a maximal tumor accumulation 24 h after the administration. Capitalizing on this, further experiments were performed 24 h after the intravenous injection into the tail vein of Ir6@EXO-A. For an insight into the therapeutic properties, A375 tumor-bearing mice were administered with phosphate-buffered saline or Ir6@EXO-A (Ir dose of 5 mg kg^−1^) and afterward kept in the dark or exposed to two-photon irradiation (720 nm, 50 mW, 300 s). The tumor volume and the body weight of the animal models were monitored every two days for 16 days. Compared with other groups, treating Ir6@EXO-A and exposure to light irradiation caused a strong tumor growth inhibition effect and nearly entirely eradicated the tumor (Fig. 4c, d and f). Importantly, no changes in the weight or signs of pain, stress, or discomfort of the animal were observed during the 16 days (Fig. 4e). Hematoxylin and eosin stain of the major organs of the animal models did not show any histological alterations (Fig. S57†), indicative of the high biocompatibility of the treatment. The tumorous tissue analyzed by a hematoxylin and eosin stain exhibited no pathological changes in animals treated with Ir6@EXO-A in the dark. A significant amount of cell damage, including cell membrane ruptures and shrinkage, was observed in those treated with Ir6@EXO-A under light irradiation (Fig. 4g). Subsequently, the tumorous tissue was incubated with GPX4 fluorescent antibodies. While the animals treated with Ir6@EXO-A in the dark did not exhibit any alterations, the tumor slices of the mice models treated with Ir6@EXO-A and exposed to irradiation showed strongly reduced levels of GPX4 (Fig. 4h), indicative of the cell death by ferritinophagy.

### *In vivo* immunotherapy

**Fig. 5:**
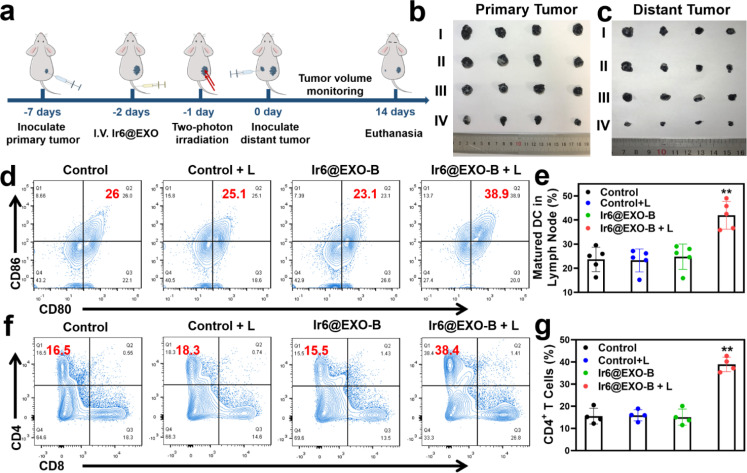
Therapeutic properties of Ir6@EXO-B in primary and secondary B16–F10 tumor-bearing mouse model. (a) Schematic illustration of the treatment protocol. (b) Photographs of the primary tumor after treatment. (c) Photographs of the secondary tumor after treatment. I: control, II: control + L, III: Ir6@EXO-B, IV: Ir6@EXO-B + L. (d) Representative flow cytometry plots and (e) quantitative analysis results of lymphatic derived maturation DCs after gating on CD45^+^ CD11c^+^ T cells in the lymphatic under different treatments. (f) Representative flow cytometry plots and (g) quantitative analysis results of CD8^+^ and CD4^+^ T cells after gating on CD45^+^ CD3^+^ T cells in the primary tumors under different treatments. L = two-photon laser irradiation at 720 nm, 50 mW, 300 s. *n* = 5. **p < 0.01.

Based on the ability of Ir6 to induce immunogenic cell death, the immunotherapeutic effect was studied inside an animal model. To exclude immune rejection and maintain tumor homing targeting, exosomes were extracted from B16–F10 cells to encapsulate Ir6 and assess its immunological impact in B16–F10 tumor-bearing mice. For this purpose, Ir6 was encapsulated with B16–F10 EXOs analogously as for the A375 EXOs, referred to as Ir6@EXO-B (Fig. S58 and S59†). To study the immunotherapeutic effect, mice with a 50 mm^3^ primary B16–F10 tumor were treated with phosphate-buffered saline or Ir6@EXO-B upon intravenous injection. The mice were kept in the dark or exposed to two-photon irradiation (720 nm, 50 mW, 300 s) after 24 h. Subsequently, B16–F10 cells were subcutaneously injected on the other side to form a distant/secondary tumor (Fig. 5a). The tumor volume and the body weight of the animal models were monitored every two days for 14 days. A strong reduction of the primary tumor volume (Fig. 5b and S60a†) was observed, while the secondary/distant tumor volume was also significantly affected, although this tumor was not irradiated (Fig. 5c and S60b†). Importantly, no changes in the weight of the animal models were observed (Fig. S60c†). Hematoxylin and eosin stains of the major organs of the animal models did not show any histological alterations (Fig. S61†), indicative of the high biocompatibility of the treatment. After the treatment, the tumorous tissue was collected and further analyzed. While no pathological changes were observed in animals treated with Ir6@EXO-B in the dark, cell membrane ruptures and shrinkages were observed in the tumors treated with Ir6@EXO-B under light irradiation (Fig. S62†). To investigate the immunogenic properties, the maturation of dendritic cells and activated T cells in the lymph nodes, tumors, and spleen were studied by flow cytometry based on the gating strategy (Schemes S2–S4†). The mice receiving Ir6@EXO-B under light irradiation treatment presented a higher DCs maturation rate (39.6 ± 4.6%) in comparison with the control group (23.7 ± 5.0%), demonstrating that Ir6@EXO-B under light irradiation was beneficial for the immune activation in tumor-draining lymph nodes (Fig. 5d and e). The amount of cytotoxic CD4^+^ T cells was enhanced from 17.7 ± 4.3% to 39.1 ± 3.0% upon treatment with Ir6@EXO-B and exposure to light (Fig. 5f and g), indicative of a potent T cell-mediated immune response in the primary tumor. Despite cytotoxic T cells, the immunogenic effect could be limited due to regulatory T cells (CD3^+^ CD4^+^ CD25^+^ FOXP3^+^ gated on CD4^+^ T cells). Promisingly, the treatment with Ir6@EXO-B and light irradiation decreased the levels of regulatory T cells from 24.5 ± 4.6% to 12.6 ± 4.7%, thereby inhibiting the immunosuppressive properties of the tumor (Fig. S63a and b†). The amount of effector memory T cells (CD3^+^ CD8^+^ CD44^+^ CD62L^−^ gated on CD8^+^ T cells) was examined to evaluate whether the treatment triggers a long-term immune response. Encouragingly, the effector memory T cell levels increased from 10.2 ± 4.7% to 28.1 ± 4.1% (Fig. S63c and d), indicating a long-term immune response in the primary tumor. Complementary to this, the levels of regulatory T cells decreased, and the cytotoxic T cells and effector memory T cells were enhanced in the secondary tumor and spleen (Fig. S64 and S65†). These results suggest that treating Ir6@EXO-B and exposure to light irradiation can promote dendritic cell maturation and reprogram the tumor immune-suppressive microenvironment, resulting in a solid anticancer immune response.

## Conclusion

This study reports on encapsulating cyclometalated Ir(iii)-based photosensitizers within exosomes for tumor-targeted photodynamic immunotherapy. Specifically, a series of 1-methyl-4-phenylpyridinium functionalized Ir(iii) complexes with various ancillary ligands were synthesized and photophysically evaluated. The most promising derivative was studied in-depth against human malignant melanoma cells. This organometallic complex was found to selectively accumulate in the mitochondria of cancer cells, producing a mixture of singlet oxygen and superoxide anion radicals upon two-photon irradiation. This oxidative stress induced the loss of the mitochondria membrane potential and perturbed the mitochondrial respiratory chain, disrupting mitochondrial oxidative phosphorylation and glycolysis metabolisms. These biological alterations triggered cell death by ferritinophagy, a very uncommon type of cell death, and immunogenic cell death. The metal complex was encapsulated within melanoma exosomes to enhance the pharmacological properties and provide cancer selectivity to form tumor-targeted nanoparticles. Upon intravenous injection, the nanoparticles could nearly fully eradicate a human malignant melanoma tumor inside a mouse model upon two-photon irradiation at 720 nm. A mouse model with a primary and distant secondary murine melanoma tumor was prepared to investigate the immunotherapeutic properties. While only the primary tumor was treated with light, a solid therapeutic effect was observed in the primary and secondary tumors. Immunogenic investigations revealed that the nanoparticles could promote dendritic cell maturation and reprogram the tumor immune-suppressive microenvironment, resulting in a strong long-term anticancer immune response. As the treatment of melanoma tumors remains challenging in clinics due to elevated rates of drug resistance and rapid metastasis formation, there is a need for the development of new therapeutic mechanisms and anticancer strategies. Combining ferritinophagy and immunogenic cell death mechanisms could open new avenues in anticancer drug development.

## Statistical analysis

The significance of several experimental results was analyzed by using the analysis of *T*-test. Probabilities *p* < 0.05 (*) and *p* < 0.01 (**), ***P < 0.001 were marked in figures and 0.05 was chosen as the significance level.

## Ethical statement

This study was conducted using Animal Care and Institutional Ethical Guidelines in China and protocols approved by the Sun Yat-Sen University Animal Care and Use Committee (certificate number: SYSU-IACUC-2022-000380).

## Data availability

ESI† is available and includes experimental materials and methods for the synthesis, preparation, and characterization of the cyclometalated Ir(iii)-based photosensitizers, *in vitro* antitumor efficiency, immune activation, and *in vivo* animal experiments.

## Author contributions

H. Chao oversaw and designed all experiments; T. Feng synthesized the complex; T. Feng, Z. Tang, K. Xiong, and C. Jin performed the photodynamic experiments in the solution and in the cancer cells; T. Feng, Z. Tang and J. Shu performed the animal experiments; T. Feng, Y. Chen, J. Karges, G. Gasser and H. Chao analyzed the data and wrote the manuscript. All authors discussed the results and commented on and proofread the manuscript.

## Conflicts of interest

The authors declare no competing interests.

## Supplementary Material

SC-015-D4SC01214C-s001
