# Supplementary material for: An iridium(iii)-based photosensitizer disrupting the mitochondrial respiratory chain induces ferritinophagy-mediated immunogenic cell death
Source: Chem Sci. 2024 Apr 2;15(18):6752–62. doi: 10.1039/d4sc01214c (PMC11077511; doi:10.1039/d4sc01214c)
Supplement: SC-015-D4SC01214C-s001 [file SC-015-D4SC01214C-s001.pdf]

Electronic Supplementary Material (ESI) for Chemical Science.
This journal is © The Royal Society of Chemistry 2024

Supporting information

An iridium(III)-based photosensitizer disrupting the mitochondrial respiratory chain induces ferritinophagy-mediated immunogenic cell death

Tao Feng,[a] Zixin Tang,[a] Johannes Karges,[b] Jun Shu,[a] Kai Xiong,[a] Chengzhi Jin,[a] Yu Chen,[a] Gilles Gasser,*[c] Liangnian Ji,[a] and Hui Chao*[a,d]

[a] MOE Key Laboratory of Bioinorganic and Synthetic Chemistry, State Key Laboratory of Anti-Infective Drug Discovery and Development, Guangdong Basic Research Center of Excellence for Functional Molecular Engineering, School of Chemistry, Guangdong Provincial Key Laboratory of Digestive Cancer Research, The Seventh Affiliated Hospital, Sun Yat-Sen University, Guangzhou, 510006, P. R. China. Email: ceschh@mail.sysu.edu.cn.

[b] Faculty of Chemistry and Biochemistry, Ruhr-University Bochum, Universitätsstrasse 150, 44780 Bochum, Germany.

[c] Chimie ParisTech, PSL University, CNRS, Institute of Chemistry for Life and Health Sciences, Laboratory for Inorganic Chemical Biology, 75005 Paris, France. Email: gilles.gasser@chimieparistech.psl.eu.

[d] MOE Key Laboratory of Theoretical Organic Chemistry and Functional Molecule, School of Chemistry and Chemical Engineering, Hunan University of Science and Technology, Xiangtan, 400201, P. R. China.

*Co-corresponding authors

Contents

Experimental Procedures **6**

Materials and Instrumentation **6**

Synthesis and Characterization 7

Partition-coefficient (Log *P*) measurement 10

Singlet oxygen quantum yield measurement 10

Two-photon absorption cross-section measurement 11

Stability in FBS and culture media 11

Identification of type of ROS 11

Exosome extraction and Ir6 encapsulation 12

Two-photon cellular imaging 12

Localization studied by confocal laser scanning microscopy 12

Localization studied by ICP-MS 13

In vitro cytotoxicity 13

Reactive oxygen species generation during in vitro photodynamic and Fenton treatment 13

Evaluation of mitochondrial oxidative phosphorylation and glycolysis processes 14

Evaluation of intracellular Fe^{2+} 14

Western Blot analysis 14

Cell-survival assay and autophagy analysis 15

Lipid peroxidation 15

Immunofluorescence staining of calreticulin in cells. 15

Immunofluorescence staining of extracellular HMGB1 in cells. 16

Extracellular HMGB1 was detected by ELISA kit. 16

Extracellular ATP detection assay in cells and MCTS 16

Immunofluorescence staining of HSP70 in cells 17

Cellular uptake assay between different cell lines 17

One- and two-photon excited cellular images of 3D multi-cellular spheroids 17

In vivo / in vitro fluorescence imaging and biodistribution 17

In vivo particle excretion and blood circulation time 18

Animal and tumor models 18

In vivo immunity experiment. 18

Ethics statement 21

References **22**

Results and Discussion **23**

Figure S1. .... **23**

Figure S2. .... **24**

Figure S3. .... **25**

Figure S4. .... **26**

Figure S5. .... **27**

Figure S6. .... **28**

Figure S7. .... **29**

Figure S8. .... **30**

Figure S9. .... **31**

Figure S10. .... **32**

Figure S11. .... **33**

Figure S12. .... **34**

Figure S13. .... **35**

Figure S14. .... **36**

Figure S15. .... **37**

Figure S16. .... **38**

Figure S17. .... **39**

Figure S18. .... **40**

Figure S19. .... **41**

Figure S20. .... **42**

Figure S21. .... **43**

Figure S22. .... **44**

Figure S23. .... **45**

Figure S24. 46
Figure S25. 47
Figure S26. 48
Figure S27. 49
Figure S28. 50
Figure S29. 51
Figure S30. 52
Figure S31. 53
Figure S32. 54
Figure S33. 55
Figure S34. 56
Figure S35. 57
Figure S36. 58
Figure S37. 59
Figure S38. 60
Figure S39. 61
Figure S40. 62
Figure S41. 63
Figure S42. 64
Figure S43. 65
Figure S44. 66
Figure S45. 67
Figure S46. 68
Figure S47. 69
Figure S48. 70
Figure S49. 71
Figure S50. 72
Figure S51. 73
Figure S52. 74

Figure S53. 75

Figure S54. 76

Figure S55. 77

Figure S56. 78

Figure S57. 79

Figure S58. 80

Figure S59. 81

Figure S60. 82

Figure S61. 83

Figure S62. 84

Figure S63. 85

Figure S64. 86

Figure S65. 87

Table S1.. 88

Table S2. 89

Table S3. 90

Table S4. 91

Experimental Procedures

Materials and Instrumentation

4,4'-Dibromo-2,2'-bipyridyl, (4-(Pyridin-4-yl)phenyl)boronic acid, Iodomethane and Tetrakis(triphenylphosphine)palladium were purchased from Energy Chemical. 3-(4,5-dimethylthiazol-2-yl)-2,5-diphenyltetrazolium bromide (MTT), 2,2,6,6-tetramethylpiperidine (TEMPO), 1,1'-dioctadecyl-3,3,3',3'-tetramethylindocarbocyanine perchlorate (Dil), 5,5-Dimethyl-1-pyrroline N-oxide (DMPO), 9,10-Anthracenediyl-bis(methylene)dimalonic acid (ABDA), and 2,7-dichlorodihydrofluorescein diacetate (DCFH-DA) were purchased from Sigma Aldrich. FerroOrange was purchased from Dojindo Molecular Technologies. Mito-Tracker™ Deep Red, Lyso-Tracker™ Deep Red, ER-Tracker™ Red, and live/dead cell imaging kits were purchased from Thermo Fisher Scientific. Cell death inhibitor z-VAD-fmk, Necrostatin-1 (Nec-1), Ferrostatin-1 (Fer-1), Deferoxamine (DFO), Disulfiram and 3-Methyladenine (3-MA) were obtained from Shanghai Biyuntian Biotechnology Co., LTD.. Anti-NCOA4 antibody (ab86707), Anti-FLH-1 antibody (ab75972), Anti-GPX4 antibody (ab125066), and Anti-LC3B antibody (ab192890) were purchased from Abcam. Compounds $[Ir(ppy)_2Cl]_2$, $[Ir(pytz)_2Cl]_2$, $[Ir(dbq)_2Cl]_2$, $[Ir(pbt)_2Cl]_2$, $[Ir(mo\text{-}pbt)_2Cl]_2$, and $[Ir(dfppy)_2Cl]_2$ were synthesized according to the corresponding literature methods.[1]

^{1}H NMR and ^{13}C NMR spectra were performed on a nuclear magnetic resonance spectrometer (Bruker Avance III, 400 MHz). High-resolution electrospray ionization mass spectra (HRMS) were recorded by ESI-Q-TOF maxis 4G (Bruker Daltonics). Microanalyses (C, H, N, and S) were determined by an elemental analyzer (Elementar, Vario EL Cube). The absorption and emission spectra were recorded using a Perkin-Elmer Lambda 950 UV/Vis spectrometer and a Perkin-Elmer RF-5301 luminescence spectrometer. The two-photon was performed on a Femtosecond Fluorescence Spectrum Measurement System (Coherent Inc). Transmission electron microscopy (TEM) images were recorded with a spherical aberration-corrected transmission electron microscope (JEM-ARM200P). Dynamic light scattering (DLS) measurements were performed with a Zetasizer Nano ZS analyzer (EliteSizer). Electron spin resonance (ESR) spectra were measured using a Bruker Model A300 spectrometer. The metal content of biological samples was determined by inductively coupled plasma mass spectrometry (ICP-MS) with a Thermo Scientific ICAP RQ series ICP-MS instrument. Confocal microscopy images were recorded on the LSM 880 NLO (Zeiss) microscope. Flow Cytometry was performed on a BD

FACS Canton II flow cytometer. In vivo/Ex vivo images were recorded on an IVIS Lumina XRMS Series III imaging system (PerkinElmer).

Synthesis and Characterization

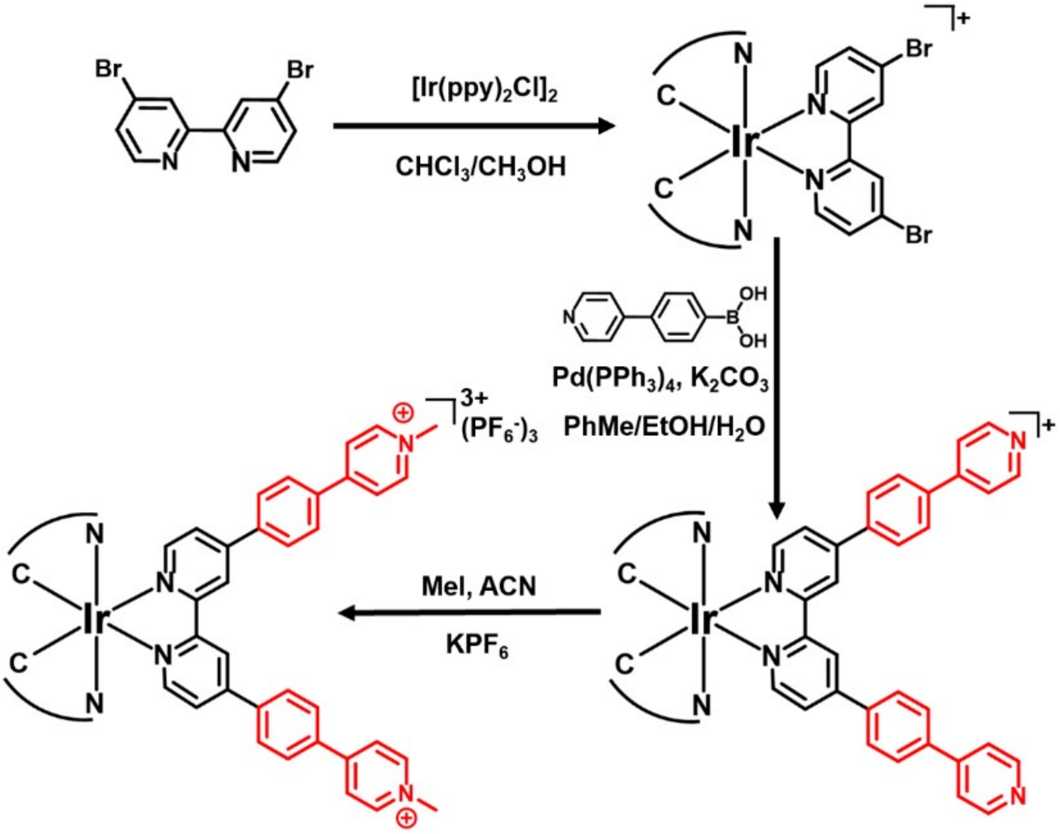

Scheme S1. Synthesis strategy for the preparation of **Ir1**-**Ir6**.

Synthesis of [(ppy)$_2$Ir(bpy-Br$_2$)]Cl: 4,4'-Dibromo-2,2'-bipyridyl (66 mg, 0.21 mmol, 2 eq.) and **[Ir(ppy)$_2$Cl]$_2$**[1] (107 mg, 0.1 mmol, 1 eq.) were dissolved in a mixture of methanol (10 mL) and chloroform (10 mL) under argon atmosphere. The solution was heated at reflux for 6 h. After cooling to room temperature, the solvent was removed under reduced pressure, and the crude product was purified by alumina flash chromatography (dichloromethane/methanol, 30:1, v/v) to obtain a yellow solid (130 mg, 80%). ^{1}H NMR (400 MHz, DMSO-d_6) δ = 9.35 (d, J = 2.1 Hz, 2H), 8.30 – 8.23 (m, 2H), 8.02 – 7.89 (m, 6H), 7.75 (dd, J = 5.8, 1.2 Hz, 2H), 7.66 (d, J = 5.9 Hz, 2H), 7.17 (ddd, J = 7.3, 5.7, 1.4 Hz, 2H), 7.02 (td, J = 7.5, 1.3 Hz, 2H), 6.90 (td, J = 7.3, 1.3 Hz, 2H), 6.15 (dd, J = 7.6, 1.1 Hz, 2H) ppm**;** ESI-MS m/z: [M]$^+$ calcd. for $C_{32}H_{22}Br_2IrN_4$, 812.9; found: 813.1.

Synthesis of [(ppy)$_2$Ir(bpy-MPP$_2$)]Cl: [(ppy)$_2$Ir(bpy-Br$_2$)]Cl (81 mg, 0.1 mmol, 1 eq.), (4-(Pyridin-4-yl)phenyl)boronic acid (80 mg, 0.4 mmol, 4 eq.), and potassium carbonate (42 mg, 0.3 mmol, 3 eq.) were dissolved in a mixture of toluene (25 mL), ethanol (5 mL) and distilled water (5 mL) under argon atmosphere. After 1 h, palladium-tetrakis(triphenylphosphine) (20 mg, 0.01 mmol, 0.1 eq.) was added and the solution was heated at 100 °C overnight. After cooling to room temperature, the solvent was removed under reduced pressure, and the crude product was purified by alumina flash chromatography (dichloromethane/methanol, 30:1, v/v) to obtain a yellow solid (120 mg, 75%). ^{1}H NMR (400 MHz, DMSO-d_6) δ = 9.48 (d, J = 2.1 Hz, 2H), 8.73 – 8.69 (m, 4H), 8.34 – 8.30 (m, 2H), 8.28 – 8.24 (m, 4H), 8.17 (dd, J = 5.9, 1.8 Hz, 2H), 8.12 – 8.07 (m, 4H), 8.00 – 7.93 (m, 6H), 7.88 – 7.84 (m, 4H), 7.80 (dt, J = 5.7, 1.1 Hz, 2H), 7.22 (ddd, J = 7.4, 5.9, 1.4 Hz, 2H), 7.07 (td, J = 7.5, 1.2 Hz, 2H), 6.96 (td, J = 7.4, 1.3 Hz, 2H), 6.26 (dd, J = 7.6, 1.3 Hz, 2H) ppm; ESI-MS m/z: [M]$^+$ calcd. for $C_{54}H_{38}IrN_6$, 963.2; found: 963.1.

Synthesis of [(ppy)$_2$Ir(bpy-MPP+$_2$)](PF$_6$)$_3$ (Ir1): [(ppy)$_2$Ir(bpy-MPP$_2$)]Cl (96 mg, 0.1 mmol, 1 eq.) was dissolved in acetonitrile (5 mL). Iodomethane (75 mg, 0.5 mmol, 5 eq.) was added and stirred upon heating at 90 °C overnight. After this time, distilled water was added to quench the reaction. The resulting yellow precipitate was then isolated by filtration, washed with water and diethyl ether, and dried under a vacuum. The crude product was purified by alumina flash chromatography (dichloromethane/methanol, 1:1, v/v) to obtain a yellow solid. The powder was dissolved into a small amount of DMF, and a saturated solution of potassium hexafluorophosphate in water/ethanol (4:1, v/v) was added. The precipitation is collected by filtration (45 mg, 46%). ^{1}H NMR (400 MHz, DMSO-d_6) δ = 9.49 (s, 2H), 9.08 (s, 4H), 8.64 (s, 4H), 8.35 (s, 10H), 8.21 (d, J = 5.8 Hz, 2H), 7.99 (d, J = 6.6 Hz, 6H), 7.80 (d, J = 5.7 Hz, 2H), 7.22 (t, J = 6.7 Hz, 2H), 7.08 (t, J = 7.6 Hz, 2H), 6.97 (t, J = 7.5 Hz, 2H), 6.26 (d, J = 7.5 Hz, 2H), 4.38 (s, 6H) ppm. ^{13}C NMR (101 MHz, DMSO-d_6) δ = 167.4, 156.5, 153.5, 151.1, 150.6, 149.6, 149.1, 146.2, 144.3, 139.4, 139.0, 135.7, 131.6, 130.8, 129.4, 129.3, 126.8, 125.7, 124.8, 124.5, 123.3, 122.9, 120.6, 47.7 ppm. HRMS m/z: [M]$^{3+}$ calcd. for $C_{56}H_{44}IrN_6$, 331.10801; found: 331.10782. Elemental analysis calcd (%) for $C_{56}H_{44}F_{18}IrN_6P_3 \bullet 2H_2O$, C: 45.94%, H: 3.30%, N: 5.74%, found : C: 45.87%, H:3.51%, N: 5.81%. HPLC purity: t_R = 3.610, 99.438%.

Synthesis of [(pytz)$_2$Ir(bpy-MPP+$_2$)](PF$_6$)$_3$ (Ir2): This complex was synthesized in a manner identical to that described for the preparation of **Ir1**, using pytz as the ligand. Yield: 32.5 mg, 32.3 µmol, 43.5%. ^{1}H NMR (400 MHz, DMSO-d_6) δ = 9.47 (d, J = 2.2 Hz, 2H), 9.10 – 9.07 (m, 4H), 8.67 – 8.64 (m, 4H), 8.36 (s, 8H), 8.25 (dd, J = 5.9, 1.8 Hz, 2H), 7.94 (d, J = 5.9 Hz, 2H), 7.87 – 7.79 (m, 4H), 7.73 – 7.68 (m, 4H), 7.01 (ddd, J = 7.5, 5.9, 1.8 Hz, 2H), 6.23 (d, J = 4.7 Hz, 2H), 4.38 (s, 6H) ppm. ^{13}C NMR (101 MHz, DMSO-d_6) δ = 156.7, 153.4, 153.1, 151.2, 150.2, 149.1, 146.2, 139.9, 138.9,

136.7, 135.7, 131.6, 130.8, 129.5, 129.4, 127.0, 124.8, 123.3, 121.6, 118.9, 47.7 ppm. HRMS m/z: $[M]^{3+}$ calcd. for $C_{52}H_{40}IrN_6S_2$, 335.07896; found: 335.07857. Elemental analysis calcd (%) for $C_{52}H_{40}F_{18}IrN_6P_3S_2$ • H_2O, C: 42.31%, H: 3.00%, N: 5.69%, S: 4.34%, found : C: 42.61%, H: 3.27%, N: 6.12%, S: 4.67%. HPLC purity: t_R = 5.702, 96.644%.

Synthesis of [(dbq)$_2$Ir(bpy-MPP+$_2$)](PF$_6$)$_3$ (Ir3): This complex was synthesized in a manner identical to that described for the preparation of **Ir1**, using dbq as the ligand. Yield: 39.7 mg, 34.7 µmol, 39.7%. ^{1}H NMR (400 MHz, DMSO-*d*$_6$) δ = 9.54 (d, *J* = 2.4 Hz, 2H), 9.20 (dd, *J* = 8.0, 1.4 Hz, 2H), 9.07 (d, *J* = 6.6 Hz, 3H), 8.92 (d, *J* = 3.1 Hz, 2H), 8.87 (d, *J* = 8.5 Hz, 2H), 8.67 – 8.60 (m, 4H), 8.35 (dtd, *J* = 16.3, 8.8, 8.2, 4.1 Hz, 12H), 8.16 – 8.08 (m, 3H), 7.99 (ddd, *J* = 11.5, 5.9, 2.8 Hz, 4H), 7.91 (t, *J* = 7.6 Hz, 2H), 7.37 (dd, *J* = 8.9, 6.6 Hz, 2H), 6.54 (dd, *J* = 7.3, 3.0 Hz, 2H), 4.37 (s, 6H) ppm. ^{13}C NMR (101 MHz, DMSO-*d*$_6$) δ = 156.9, 153.5, 151.7, 149.5, 149.2, 146.2, 143.5, 143.0, 138.9, 138.0, 135.8, 132.9, 132.6, 132.0, 131.5, 129.9, 129.4, 129.3, 129.0, 128.9, 127.0, 125.3, 124.8, 124.5, 123.3, 117.1, 47.7 ppm. HRMS m/z: $[M]^{3+}$ calcd. for $C_{66}H_{46}IrN_8$, 381.11527; found: 381.11497. Elemental analysis calcd (%) for $C_{66}H_{46}F_{18}IrN_8P_3$ • H_2O, C: 49.66%, H: 3.03%, N: 7.02%, found : C: 49.92%, H: 3.27%, N: 7.36%. HPLC purity: t_R = 5.618, 97.469%.

Synthesis of [(pbt)$_2$Ir(bpy-MPP+$_2$)](PF$_6$)$_3$ (Ir4): This complex was synthesized in a manner identical to that described for the preparation of **Ir1**, using pbt as the ligand. Yield: 35 mg, 28.8 µmol, 39.5%. ^{1}H NMR (400 MHz, DMSO-*d*$_6$) δ = 9.44 (d, *J* = 8.1 Hz, 2H), 9.07 (d, *J* = 6.4 Hz, 4H), 8.63 (d, *J* = 6.4 Hz, 4H), 8.38 – 8.24 (m, 12H), 8.15 (d, *J* = 5.8 Hz, 2H), 8.08 (d, *J* = 7.8 Hz, 2H), 7.44 (t, *J* = 7.8 Hz, 2H), 7.21 (dt, *J* = 16.6, 7.8 Hz, 4H), 6.99 (t, *J* = 7.6 Hz, 2H), 6.36 (d, *J* = 7.6 Hz, 2H), 6.31 (d, *J* = 8.5 Hz, 2H), 4.37 (s, 6H) ppm. ^{13}C NMR (101 MHz, DMSO-*d*$_6$) δ = 181.8, 162.8, 157.1, 153.5, 151.1, 149.5, 149.2, 146.2, 140.5, 138.6, 135.9, 133.2, 132.7, 131.8, 129.5, 129.3, 128.8, 127.6, 127.1, 126.5, 125.3, 124.8, 123.7, 123.2, 117.5, 47.7 ppm. HRMS m/z: $[M]^{3+}$ calcd. for $C_{60}H_{44}IrN_6S_2$, 368.42272; found: 368.42242. Elemental analysis calcd (%) for $C_{60}H_{44}F_{18}IrN_6P_3S_2$ • $3H_2O$, C: 45.20%, H: 3.16%, N: 5.27%, S: 4.02%, found : C: 45.04%, H: 3.00%, N: 5.10%, S: 3.74%. HPLC purity: t_R = 5.714, 96.096%.

Synthesis of [(mo-pbt)$_2$Ir(bpy-MPP+$_2$)](PF$_6$)$_3$ (Ir5): This complex was synthesized in a manner identical to that described for the preparation of **Ir1**, using mo-pbt as the ligand. Yield: 41 mg, 35.2 µmol, 38%. ^{1}H NMR (400 MHz, DMSO-*d*$_6$) δ = 9.45 (d, *J* = 2.2 Hz, 2H), 9.08 (d, *J* = 6.5 Hz, 4H), 8.63 (d, *J* = 6.7 Hz, 4H), 8.42 – 8.29 (m, 10H), 8.28 – 8.17 (m, 4H), 8.06 (d, *J* = 8.6 Hz, 2H), 7.39 (t, *J* = 7.7 Hz, 2H), 7.20 (td, *J* = 7.9, 7.3, 1.3 Hz, 2H), 6.82 (dd, *J* = 8.7, 2.5 Hz, 2H), 6.25 (d, *J* = 8.4 Hz, 2H), 5.76 (d, *J* = 2.4 Hz, 2H), 4.37 (s, 6H), 3.53 (s, 6H) ppm. ^{13}C NMR (101 MHz, DMSO-*d*$_6$) δ = 180.9, 162.3, 157.1, 153.5, 153.3, 151.2, 149.4, 149.2, 146.2, 138.6, 135.9, 133.4, 131.3, 129.7, 129.5, 129.3, 128.6, 127.1, 126.0, 125.0, 124.8, 123.2, 118.7, 117.0, 108.8, 55.2, 47.7 ppm. HRMS m/z: $[M]^{3+}$ calcd. for $C_{62}H_{48}IrN_6O_2S_2$, 388.42976;

found: 388.42938. Elemental analysis calcd (%) for $C_{62}H_{48}F_{18}IrN_6P_3S_2O_2 \bullet 2H_2O$, C: 45.51%, H: 3.20%, N: 5.14%, S: 3.92%; found : C: 45.24%, H: 3.47%, N: 5.24%, S: 3.65%. HPLC purity: t_R = 5.743, 96.187%.

Synthesis of [(dfppy)$_2$Ir(bpy-MPP+$_2$)](PF$_6$)$_3$ (Ir6): This complex was synthesized in a manner identical to that described for the preparation of **Ir1**, using dfppy as the ligand. Yield: 37.7 mg, 35.2 µmol, 37.7%. ^{1}H NMR (400 MHz, methanol-d_4) δ = 9.41 (d, *J* = 1.9 Hz, 2H), 8.96 (d, *J* = 6.5 Hz, 4H), 8.53 – 8.48 (m, 4H), 8.42 (d, *J* = 8.7 Hz, 2H), 8.35 (d, *J* = 8.4 Hz, 4H), 8.26 (d, *J* = 8.3 Hz, 4H), 8.17 (d, *J* = 5.8 Hz, 2H), 8.06 (dd, *J* = 5.9, 1.8 Hz, 2H), 8.00 (td, *J* = 8.0, 1.5 Hz, 2H), 7.88 (dd, *J* = 6.0, 1.5 Hz, 2H), 7.21 (ddd, *J* = 7.4, 5.9, 1.4 Hz, 2H), 6.74 (ddd, *J* = 11.9, 9.2, 2.3 Hz, 2H), 5.79 (dd, *J* = 8.4, 2.4 Hz, 2H), 4.45 (s, 6H) ppm. ^{13}C NMR (101 MHz, DMSO-d_6) δ = 163.3, 163.2, 162.5, 162.2, 162.0, 160.0, 156.3, 155.5, 153.4, 150.9, 150.3, 149.6, 146.3, 140.7, 138.8, 135.82, 129.5, 129.4, 128.1, 127.1, 125.2, 124.8, 123.7, 47.7 ppm. HRMS m/z: $[M]^{3+}$ calcd. for $C_{56}H_{40}F_4IrN_6$, 355.09545; found: 355.09515. Elemental analysis calcd (%) for $C_{56}H_{40}F_{22}IrN_6P_3 \bullet 2CH_3CH_2OH$, C: 45.26%, H: 3.29%, N: 5.28%, , found: C: 45.11%, H:3.68%, N: 5.28%. HPLC purity: t_R = 5.737, 99.351%.

Partition-coefficient (Log *P*) measurement

The partition coefficient of complexes was determined by the "shake-flask" method. Water and octanol were mixed and thoroughly shaken to equilibrium, and the two resulting layers were separated. The indicated complexes were dissolved in the octanol phase, which was previously saturated with water to give a 3 mL solution. The same volume of water phase previously saturated with octanol was added into the solution. The mixture was shaken vigorously at room temperature overnight and then stood for 24 h. The concentration of **Ir1-Ir6** was determined by UV-vis spectroscopy using extinction coefficients of the complexes in octanol saturated with water. The evaluation was replicated three times.

Singlet oxygen quantum yield measurement

The singlet oxygen quantum yield of the compounds was determined in methanol with Methylene Blue (Φ = 0.52[2]) as the reference according to a reported procedure. The quantum yield was calculated by the following equation:

$$\frac{k}{k^s} = \frac{\Phi_{ab}\Phi_\Delta}{\Phi_{ab}^s\Phi_\Delta^s}$$

k is the slope of the curve that the data points fit, Φ_{ab} is the light absorption efficiency of excitation light, Φ_{Δ} is the singlet oxygen yield. The parameter superscript s stands for the standard (MB).

Two-photon absorption cross-section measurement

The two-photon absorption (TPA) cross-section was measured using a previously reported method.[2-3] **Ir1-Ir6** in methanol at a 0.2 mM concentration was placed in a fluorometric quartz cuvette at 25 °C. The TPA spectra of **Ir1-Ir6** were determined from 700-800 nm. And the cross-section values are calculated at each wavelength according to the equation as follows:

$$\delta_S = \delta_R \cdot \frac{\Phi_R C_R I_S n_S}{\Phi_S C_S I_R n_R}$$

Where *δ* is the two-photon absorption cross-section, *Φ* is the quantum yield, *C* is the concentration, *I* is the integrated emission intensity at each wavelength, and *n* is the refractive index. Subscript' S' stands for samples, 'R' stands for the reference, rhodamine B.

Stability in FBS and culture media

For UV-Vis experiment, **Ir6** (10 µM) was added to the DMEM (10% FBS). The solution was incubated for 0 h, 24 h, and 48 h at 37 °C with shaking (~300 rpm), respectively. The absorption spectra of these mixtures were tested.

For HPLC-UV experiment, **Ir6** (10 µM) was added to the DMEM (10% FBS). The solution was incubated for 0 h, 24 h, and 48 h at 37 °C with shaking (~300 rpm), respectively. After that, 2 mL of acetonitrile was added, and the mixture was centrifuged for 45 min at 1000 g at 4 °C. The suspension was evaporated, and the residue was resuspended in 200 µL of acetonitrile and analyzed by HPLC-UV. A C8 reverse phase column was used with a flow rate of 0.5 mL/min. The runs were performed with a linear gradient of A (acetonitrile, Sigma-Aldrich HPLC grade) in B (distilled water). The absorption spectra of these mixtures were tested. A diazepam solution was used as an internal reference.

Identification of type of ROS

The ESR measurements were carried out with a Bruker Model A300 spectrometer at 25 °C. The variety of reactive oxygen species was identified using an electron spin resonance assay with 2,2,6,6-tetramethylpiperidine (TEMPO) as a $^1O_2$ scavenger and 5,5-dimethyl-1-pyrroline-*N*-oxide (DMPO) as a superoxide radical ($^{\bullet}O_2^-$) trapper. The electron spin resonance spectrometer was operated with 1 G field modulation and a 100 G scan range with 20 mW microwave power. TEMPO and DMPO spin-trapping adduct to detect the $^1O_2$ and $^{\bullet}O_2^-$ produced by **Ir6** and MPP+ under irradiation or in the dark. Briefly, solutions of the sample (2 μM) were irradiated by a 720 nm tow-photon laser (20 mW/cm^2) for 10 second under normoxic conditions. Afterward, the electron spin resonance spectrum was recorded.

Exosome extraction and Ir6 encapsulation

The exosomes (EXO) derived from A375 cancer cells and B16-F10 were prepared according to the standard ultracentrifugation protocol with slight modifications.[4] The cancer cells were cultured in 15 cm^2 dishes. After that, the cell medium was refreshed by a serum-free medium and continued for 48 h. Then, the medium was collected and centrifugated with 300 g, 10 min to remove dead cells, 2000 g, 10 min to remove large debris, 12000 g, and 30 min to remove other cellular contaminants. Finally, upon ultracentrifugation with a centrifugal force of 100,000–120,000 g, 90 min, the desired EXOs are isolated. To load **Ir6** into the exosomes, the **Ir6** solution (50 μM) and the EXO (0.5 mg) were mixed well in 250 μL PBS in a 0.4 cm cuvette (Bio-Rad). Electroporation was carried out at 200 V and 100 μF on an X-Porator H1 electroporation system (Etta Biotech, China). After electroporation, the mixture was incubated at 37 °C for 30 min to recover the membrane of the electroporated exosomes. The as-prepared **Ir6@EXO** were harvested and washed with 8000 g for 5 min to remove the precipitate.

Two-photon cellular imaging

The A375 cells were seeded in 35 mm^2 confocal microscopy dishes and incubated overnight. The cells were then incubated with **Ir6** (10 μM) for 6 h. The dishes were washed with PBS three times. The images were captured on a Zeiss LSM 880 NLO confocal microscope (CLSM) (63 × oil immersion objective) and analyzed using Axio Vision 4.2 software (Carl Zeiss) imaged in a Zeiss LSM 880 NLO CLSM. $\lambda_{two\text{-}photon,\ ex}$ = 720 nm, λ_{em} = 520-600 nm.

Localization studied by confocal laser scanning microscopy

A375 cells were seeded on 35 mm^2 confocal microscopy dishes and cultured for 24 h. Then cells were incubated with **Ir6** at 37 °C for 6 h. The medium was refreshed, and the cells were further incubated with Mito-Tracker™ Deep Red (50 nM), Lyso-Tracker™ Deep Red (50 nM), and ER-Tracker™ Red (50 nM) for 30 min. After that, the cells were washed three times with phosphate-buffered saline, and microscopy images were recorded with a CLSM. The fluorescence images Mito-Tracker™ Deep Red, Lyso-Tracker™ Deep Red and ER-Tracker™ Red (λ_{ex} = 633 nm, λ_{em} = 650-710 nm) were recorded using CLSM. The fluorescence images Ir6 (λ_{ex} = 405 nm, λ_{em} = 520-580 nm) were recorded using CLSM. The final concentration of DMSO in the working solution for biological assays is 0.1%.

Localization studied by ICP-MS

A375 cells were seeded on 10 cm^2 dishes and cultured for 24 h. **Ir6** was incubated with A375 cells for 6 h at 37 °C. After this, all cells were collected carefully and washed with phosphate-buffered saline three times. The cells were then divided into equal parts and counted. The cytometer, nuclei, lysosomes, and mitochondria were extracted using commercial cell organelle extraction kits following the manufacturer's protocol. The obtained samples were digested with 60% nitric acid at room temperature, and ICP-MS measured the iridium content. The final concentration of DMSO in the working solution for biological assays is 0.1%.

In vitro cytotoxicity

The cytotoxicity of **Ir6** or MPP+ toward different cell lines was investigated using the classic MTT assays. The cells were seeded in 96-well plates (1×10^4 cells/well) with DMEM medium or RPMI 1640 medium containing 10% fetal bovine serum for 24 h. The medium was replaced and added to each well with different concentrations of **Ir6** or MPP+. After 6 h, the cells were refreshed with medium containing 10% fetal bovine serum and irradiated by two-photon irradiation (720 nm, 40 mW, 120 s) or incubated under dark conditions for 24 h. MTT solution (20 µL, 5 mg/mL) was set in each well for 4 h. A microplate reader measured the optical density at 595 nm of each well. Data were reported as the means ± standard deviation (n = 3). The final concentration of DMSO in the working solution for biological assays is 0.1%.

Reactive oxygen species generation during in vitro photodynamic and Fenton treatment

Reactive oxygen species production was studied using a molecular probe as a 2',7′-dichlorofluorescein diacetate (DCFH-DA). A375 cells were seeded on 35 mm^2 confocal dishes for confocal laser scanning microscopy and 6-well plates for utilization of flow cytometry. After being incubated for 6 h, the cells were irradiated by two-photon irradiation (720 nm, 40 mW, 120 s) or incubated under dark conditions. Then, the cells were stained with DCFH-DA (10 μM) for 30 minutes according to the manufacturer's protocol. Following this procedure, fluorescence images (λ_{ex} = 488 nm, λ_{em} = 500-520 nm) were recorded using CLSM or flow cytometry. The Dihydroethidium (DHE) as a $^{\bullet}O_2^-$ probe and the fluorescence images (λ_{ex} = 563 nm, λ_{em} = 580-630 nm) were recorded using CLSM. The hydrogen peroxide assay kit as an H_2O_2 probe and the fluorescence images (λ_{ex} = 488 nm, λ_{em} = 540-570 nm) were recorded using CLSM. The HPF as a $^{\bullet}OH$ probe and the fluorescence images (λ_{ex} = 488 nm, λ_{em} = 520-570 nm) were recorded using CLSM. The final concentration of DMSO in the working solution for biological assays is 0.1%.

Evaluation of mitochondrial oxidative phosphorylation and glycolysis processes

The A375 cells were seeded in 96-well plates (Seahorse Bioscience) with a DMEM medium containing 10 % fetal bovine serum for 24 hours. The medium was replaced and added to each well with different concentrations of **Ir6** or MPP+. After 6 h, the cells were washed three times with DMEM and irradiated by two-photon irradiation (720 nm, 40 mW, 120 s) or incubated under dark conditions for 6 h. After that, XF assay medium containing 25 mM glucose, 1 mM sodium pyruvate, and 2 mM glutamine was added to the wells along with complexes to maintain a stimulating environment, and cells were equilibrated in a non-CO_2 incubator for 1 h at 37 °C. The OCR and ECAR were then determined by the Seahorse Extracellular Flux analyzer (Agilent Technologies) according to the protocols. The final concentration of DMSO in the working solution for biological assays is 0.1%.

Evaluation of intracellular Fe^{2+}

CLSM evaluated the content of ferrous ions in cells. The A375 cells were seeded into the 35 mm^2 confocal dishes. When the cell density reached about 65%, 10 μM **Ir6** and 100 μM MPP+ were mixed in a fresh culture medium and added to dishes separately. After 6 h incubation, the cells were washed twice with PBS and incubated with ferrous ion

fluorescent probe FerroOrange (1 μM) for 30 min, followed by CLSM observation. The final concentration of DMSO in the working solution for biological assays is 0.1%.

Western Blot analysis

A375 cells were incubated with **Ir6** and MPP+ without or with two-photon laser irradiation (730 nm, 40 mW, 120 s). After another 6 h incubation, cells were washed with cool PBS three times and digested with trypsin (0.25%).[5] The cells were lysed by RIPA (containing protease and phosphatase inhibitor) for 30 min at 4 °C and centrifuged at 12000 rpm for 10 min at 4 °C to obtain supernatant. After the quantitative measurement of protein concentration by the BCA protein assay kit, equal amounts of protein were added to each lane of SDS-PAGE gel for electrophoresis and then transferred onto polyvinylidene fluoride (PVDF) membranes. After blocking by primary resistance diluent and specific primary antibodies incubated at 4 °C overnight, membranes were incubated by goat anti-rabbit HRP secondary antibodies for 1 h at room temperature. The signal was developed by SuperSignal West Femto ECL (Thermo Science™ Emm™) and visualized by Omega Lum C Imaging System (Aplegen, USA). The final concentration of DMSO in the working solution for biological assays is 0.1%.

Cell-survival assay and autophagy analysis

For live/dead cell detecting, A375 cells were seeded in a 96-well plate with a density of 1 x 10^4 cells per well and incubated for 24 h. The cells were incubated with **Ir6** or MPP+ for 6 h and then exposed to irradiation (720 nm, 40 mW, 120 s). Calcein-AM (λ_{ex} = 488 nm, λ_{em} = 500-520 nm) and ethidium (λ_{ex} = 563 nm, λ_{em} = 590-630 nm) were used to stain the cells for another 30 min. Cell imaging was performed using a Zeiss LSM 880 NLO confocal microscope.

For bio-transmission electron microscope, A375 cells were treated with Control, MPP+, and **Ir6** in the dark or under irradiation and fixed overnight at 4 ºC in phosphate buffer (pH 7.4) containing 2.5% glutaraldehyde, respectively. The cells were then treated with osmium tetroxide, stained with uranyl acetate and lead citrate, and visualized with a transmission electron microscope (JEOL JEM-1400, Tokyo, Japan). Images were photographed and processed by the Eversmart Jazz program (Scitex). The final concentration of DMSO in the working solution for biological assays is 0.1%.

Lipid peroxidation.

The generation of lipid peroxidation was investigated using the specific lipid peroxide (LPO) probe C11-BODIPY (λ_{ex} = 488 nm, λ_{em} = 500-550 nm). The cells were seeded on 35 mm confocal dishes. The **Ir6** and MPP+ were added to the relevant wells and incubated in the dark for 6 h. Then, the dishes were kept in the dark or under irradiation (720 nm, 40 mW, 120 s). After 4 h, CLSM was used to detect the cells after corresponding treatments. The final concentration of DMSO in the working solution for biological assays is 0.1%.

Immunofluorescence staining of calreticulin in cells.

Confocal laser scanning microscopy: After co-incubation with the **Ir6** or MPP+ for 6 h, the dishes were exposed under irradiation (720 nm, 40 mW, 120 s) or in the dark. Cells were incubated for an additional 4 h. After that, the cells were fixed by 4% paraformaldehyde, penetrated by methanol, and stained with calreticulin (D3E6) XP® Rabbit mAb (Alexa Fluor® 488 Conjugate) overnight, and then Hoechst (λ_{ex} = 405 nm, λ_{em} = 430-460 nm) for 15 min after being washed with PBS. After washing in PBS again, the slides were imaged using the confocal microscope. Flow cytometry: The dosing treatment in this experiment is consistent with the confocal experiment. The excitation wavelength was 563 nm. Emission was collected in cells at 620 ± 20 nm. The final concentration of DMSO in the working solution for biological assays is 0.1%.

Immunofluorescence staining of extracellular HMGB1 in cells.

The cells were treated the same way as for the experiment on calreticulin with the omission of the part of penetration by methanol. The extracellular HMGB1 in cells were stained with HMGB1 Antibody and Anti-rabbit IgG (H+L), F(ab')2 Fragment (Alexa Fluor® 555 Conjugate). The excitation wavelength was 563 nm. Emission was collected in cells at 620 ± 20 nm. The final concentration of DMSO in the working solution for biological assays is 0.1%.

Extracellular HMGB1 was detected by ELISA kit.

After co-incubation with the **Ir6** or MPP+ for 6 h, the dishes were exposed under irradiation (720 nm, 40 mW, 120 s) or in the dark. Cells were incubated for an additional 4 h. The supernatant as samples was centrifuged at 10000 rpm at 4 °C for 3 minutes. The samples were then transferred to the specific 96-well plate of the HMGB1 Kit. Follow-up experiments were carried out according to the instructions. The relative contents of HMGB1 in each group under each

culture condition were expressed by the intensity ratio with the control group and were corrected by cell viability. The data is reported as the mean value ± standard deviation (n = 3). The final concentration of DMSO in the working solution for biological assays is 0.1%.

Extracellular ATP detection assay in cells and MCTS

The ATP Bioluminescence Detection Kit (Promega) recorded the detection of extracellular ATP. The supernatant of treated cells was transferred to penetrated by methanol. After co-incubation with the **Ir6** or MPP+ for 6 h, the dishes were exposed under irradiation (720 nm, 40 mW, 120 s) or in the dark. Cells were incubated for an additional 4 h. Upon completion, 100 µL supernatant was mixed with 100 µL Reagents for each assay in opaque-walled plates. TECAN Infinite M200 PRO multifunctional reader measured the Chemiluminescence Signal of ATP. Cell viability under each culture condition corrected the actual values of ATP content in each group. The data were reported as mean ± standard deviation (SD) (n = 3). The final concentration of DMSO in the working solution for biological assays is 0.1%.

Immunofluorescence staining of HSP70 in cells

Confocal laser scanning microscopy: After co-incubation with MPP+ and **Ir6** for 6 h, the dishes were exposed under irradiation (720 nm, 40 mW, 120 s) or in the dark. Cells were incubated for an additional 4 h. After that, the cells were fixed by 4% paraformaldehyde and stained with Anti-HSP70 Rabbit mAb and Anti-Rabbit IgG (H+L), F(ab')2 Fragment (Alexa Fluor® 555 Conjugate), and then Hoechst for 15 min after being washed with PBS. After washing in PBS again, the slides were imaged using a confocal microscope. The excitation wavelength was 563 nm. Emission was collected in cells at 620 ± 20 nm. The final concentration of DMSO in the working solution for biological assays is 0.1%.

Cellular uptake assay between different cell lines

A375 and HLF cells were co-incubated on 35 mm^2 plates and allowed to adhere overnight. **Ir6** or **Ir6@EXO-A** were added to the cells and incubated for 6 h. After this time, the cells were washed three times with PBS and then recorded with a CLMS. The final concentration of DMSO in the working solution for biological assays is 0.1%.

One- and two-photon excited cellular images of 3D multi-cellular spheroids

Three-dimensional multi-cellular spheroids (3D MCSs) were generated using a liquid overlay method.[6] **Ir6** or **Ir6@EXO-A** were incubated at 37 °C for 6 h. Microscopy images were recorded with a confocal laser scanning microscope (Zeiss SLS 880 NLO) upon excitation at 720 nm. The final concentration of DMSO in the working solution for biological assays is 0.1%.

In vivo / in vitro fluorescence imaging and biodistribution

A375 tumor-bearing mice were intravenously injected with **Ir6@EXO-A** (5 mg/kg). The in vivo fluorescence imaging was measured at different time points (1, 2, 4, 8, 12, 24, 48 h) with IVIS Lumina LT (λ_{ex} = 430 nm, λ_{em} = 520-580 nm). Besides, the mice were sacrificed after 24 h intravenously injected. All major organs and the tumor were collected, and performed ex vivo imaging.

In vivo particle excretion and blood circulation time

A375 tumor-bearing mice were intravenously injected with **Ir6@EXO-A** (5 mg/kg). Feces and urine were collected at different times (4, 8, 12, 24, 48, 72, and 96 h). These materials were weighed, homogenized, and dissolved in nitric acid. ICP-MS determined the iridium concentration. A375 tumor-bearing mice were intravenously injected with **Ir6@EXO-A** (5 mg/kg). After various time intervals (1, 2, 4, 8, 12, 24, 48, 72, 96 h), 10 µL of the blood of a mouse was collected through the orbit and dissolved in nitric acid. ICP-MS determined the iridium concentration.

Animal and tumor models

BALB/c nude mice (5–6 weeks) were purchased from Beijing Vital River Laboratory Animal Technology Co., Ltd. This study was conducted using Animal Care and Institutional Ethical Guidelines in China and protocols approved by the Sun Yat-Sen University Animal Care and Use Committee (certificate number: SYSU-IACUC-2022-000380). This study used a sub-tumor method to establish an animal model of subcutaneous injection of A375 cells. Each nude mouse was subcutaneously injected with 1×10^7 A375 cells suspension with 150 µL culture medium/Matrigel (1:1, v:v). About 7 days after injection, the tumor volume was allowed to grow to ~50 mm^3. The mice were randomly divided into 4 groups (n = 5 per group): (a) Control group, (b) Control + L group, (c) **Ir6@EXO-A** group, and (d) **Ir6@EXO-A** + L group. 200µL (5 mg/kg) of **Ir6@EXO-A,** or saline were i.v. injected. After 24 h, the laser groups were exposed to a two-photon (720

nm, 50 mW, 300 s). Tumor volume (V) was calculated by measuring the length (L) and width (W) and calculated as $V = W^2 \times L / 2$. After the treatment, the heart, liver, spleen, lung, kidney, brain, and tumors were stained with H&E.

***In vivo* immunity experiment.**

C57BL/6J mice aged 4–6 weeks were procured from Beijing Vital River Laboratory Animal Technology Co., Ltd. This study was performed with the Institutional Animal Care and Use Committee (IACUC) of Sun Yat-Sen University (Approval No: SYSU-IACUC-2023-000337). After 7 days of adaptive feeding, the mice were applied for the experiment. The C57BL/6J female mice were randomly divided into 3 parts, and each part was divided into 4 groups (Control, Control + L, **Ir6@EXO-B**, **Ir6@EXO-B** + L) with 5 mice. In part 1, the 4 groups (Control, Control + L, **Ir6@EXO-B**, **Ir6@EXO-B** + L) were subcutaneously injected into the right flanks of C57BL/6J mice with 2×10^5 B16-F10 cells suspension with 150 µL culture medium/Matrigel (1:1, v:v). When the tumor volume grew to 50 mm^3, 200 µL (5 mg/kg) of **Ir6@EXO-B** or saline were i.v. injected. After 24 h, the laser groups were exposed to a two-photon laser (720 nm, 50 mW, 300 s). After 5 to 7 days of feeding, the mice were euthanized. The lymph nodes selected for this procedure were the inguinal lymph nodes located next to the tumor. 500 µL of MACS buffer (consisting of 1 × PBS with 2 mM EDTA and 0.5% BSA) was added to the extracted lymph nodes, and the lymph nodes were ground. Another 500 µL of MACS buffer was added to create a suspension, which was then transferred into a centrifuge tube to obtain the cells. The cells were then resuspended in PBS for staining (PerCP-Cy5.5-CD11c, FITC-CD80, PE-CD86) at 4 °C for 30 minutes. Following the staining process, 1 × PBS was added to halt the staining, and the supernatant was removed at 500 g for 5 minutes to isolate the cells. The collected cells were once again resuspended in 1 × PBS and then analyzed using flow cytometry.

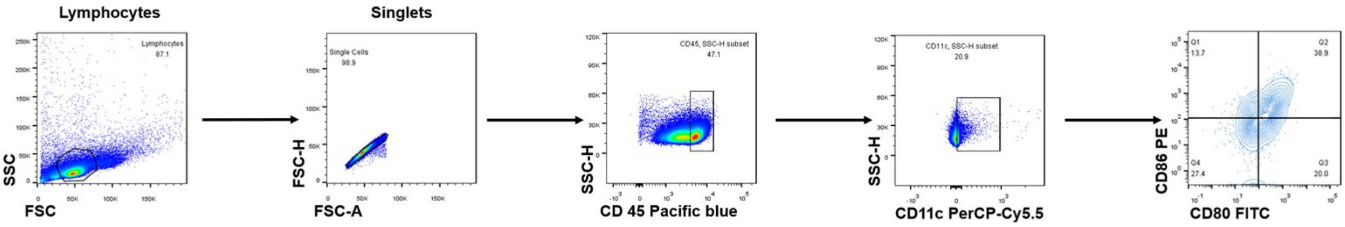

Scheme S2. Representative gating strategy for flow cytometry assay of matured DCs in tumor-draining lymph nodes of mice.

In part 2, a bilateral tumor-bearing mouse model was referenced from the manuscript and slightly modified. [6] Specifically, the 4 groups (Control, Control + L, **Ir6@EXO-B**, **Ir6@EXO-B** + L) were subcutaneously injected into the right flanks of C57BL/6J mice with 2 × 10^5 B16-F10 cells suspension with 150 μL medium (day -7). When the tumor volume grew to 50 mm^3, 200 μL (5 mg/kg) of **Ir6@EXO-B** or saline were i.v. injected (day -2). After 24 h (day -1), the laser groups were exposed to a two-photon laser (720 nm, 50 mW, 300 s). On day 0, 2×10^5 B16-F10 cells were suspended and subcutaneously injected into the left flanks of mice in each group. On days 10-12, lymphocytes from the tumors and spleens.

For tumor analysis, once the mice were euthanized, the obtained tumor was cut into small pieces using surgical scissors. The tumor fragments were rinsed with RPMI 1640 and then transferred to a 50 mL centrifuge tube. They were incubated with 10 mL of tissue digestion fluid (comprising collagenase I and DNase I) and shaken at 37 °C with a speed of 100 rpm for 20 minutes. The resulting tumor tissue suspension, after incubation, was transferred into a 70 μm filter, lightly ground, and supplemented with RPMI 1640 to a final volume of 30 mL. Following centrifugation to discard the supernatant. Subsequently, MACS solution was added, and the mixture was centrifuged at 500 g for 5 minutes. Once the tumor cells were resuspended in MACS solution, the cell density was adjusted to 2-5 × 10^7 cells/mL. For $CD8^+$ T cells and effector memory T cells experiment, 4 μL of each antibody (Pacific blue-CD45, PE-CD3, APC-CD4, SB600 (Q-Dot)-CD8, PerCP-Cy5.5-CD44, APC-CY7-CD62L) was added. For Treg cells, 4 μL of each antibody (Pacific blue-CD45, PE-Cy7-CD3, PerCP-Cy5.5-CD4, APC-CD25, PE-FOXP3) was added. The staining took place at 4 °C for 30 minutes. To halt the staining process, 1 × PBS was added, and the supernatant was removed through centrifugation (500 g, 5 minutes). The cells were then resuspended in 1 × PBS and analyzed using flow cytometry.

For spleen analysis, after euthanasia, the mice were disinfected with 75% alcohol, dissected, and their spleens were carefully removed from the super-static table. 5 mL of PBS was added, and the spleen was ground and passed through a 70 μm filter. The resulting mouse spleen cells were transferred to a 15 mL centrifuge tube and centrifuged at 500 g for 5 minutes to discard the supernatant. Next, 3 mL of red blood cell lysis solution was added at room temperature for 5 minutes. Following this, 8 mL of 1 × PBS was used to halt the process, and the mixture was centrifuged to obtain the cells (500 g, 5 minutes). The cells were then rewashed with 5 mL of 1 × PBS (500 g, 5 minutes). The collected splenic cells were suspended in 1 mL of PBS for flow staining. For $CD8^+$ T cells and effector memory T cells experiment, using a combination of antibodies (Pacific blue-CD45, PE-CD3, APC-CD4, SB600 (Q-Dot)-CD8, PerCP-Cy5.5-CD44, APC-CY7-CD62L). For Treg cells, a combination of antibodies (Pacific blue-CD45, PE-Cy7-CD3, PerCP-Cy5.5-

CD4, APC-CD25, PE-FOXP3) is used. They were stained at 4 °C for 30 minutes, and then 1 × PBS was added to halt the staining process. The mixture was centrifuged (500 g, 5 minutes) to discard the supernatant, and the cells were resuspended using 1 × PBS and subsequently analyzed via flow cytometry.

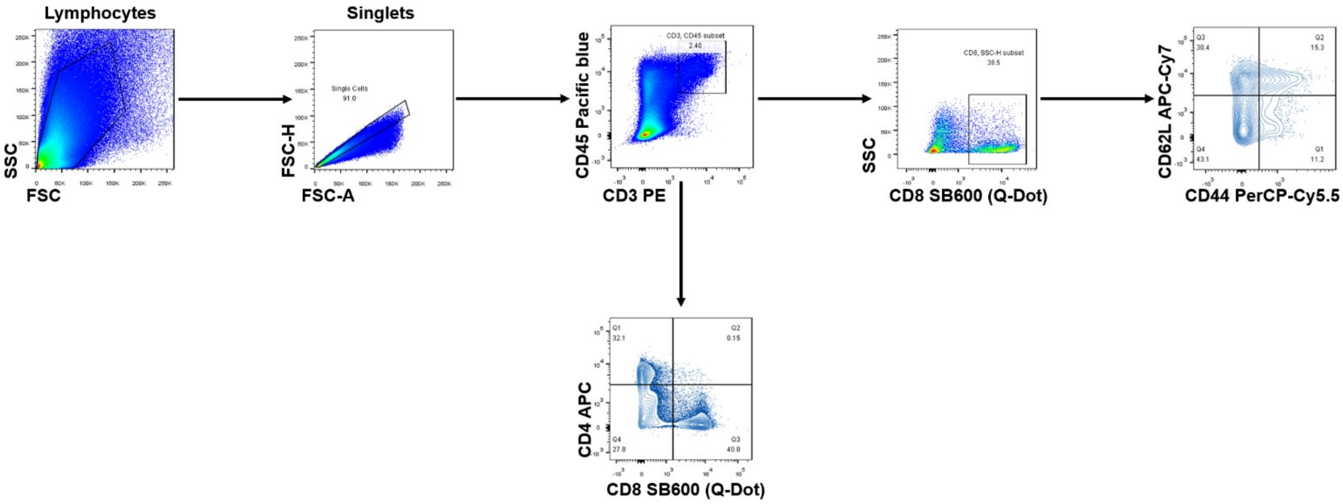

Scheme S3. Representative gating strategy for flow cytometry assay of CD8$^+$ T cells (CD45$^+$ CD3$^+$ CD8$^+$ CD4$^+$) and effector memory T cells (CD3$^+$ CD8$^+$ CD44$^+$ CD62L$^-$) in primary and distant tumors.

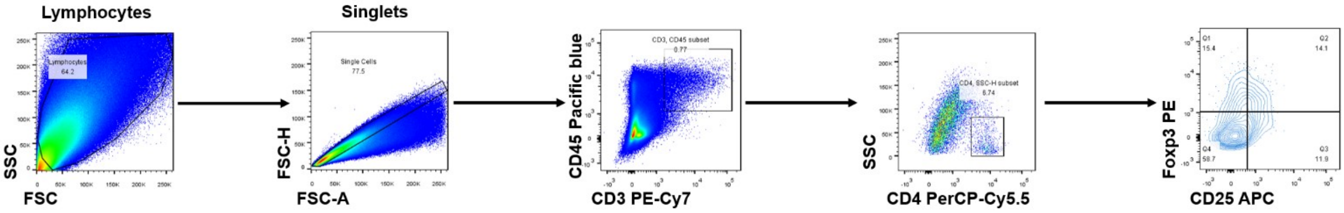

Scheme S4. Representative gating strategy for flow cytometry assay of Treg cells in both primary and distant tumors.

In part 3, the 4 groups (Control group, Control + L, **Ir6@EXO-B**, **Ir6@EXO-B** + L) were subcutaneously injected into the right flanks of C57BL/6J mice with 2 × 10^5 B16-F10 cells suspension with 150 µL medium (day -7). When the tumor volume grew to 50 mm^3, 200 µL (5 mg/kg) of **Ir6@EXO-B** or saline were i.v—Injected (day -2). After 24 h (day -1), the laser groups were exposed to a two-photon laser (720 nm, 50 mW, 300 s). On day 0, 2×10^5 B16-F10 cells were

suspended and subcutaneously injected into the left flanks of mice in each group. Tumor volume (primary and distant) was calculated by measuring the length (L) and width (W) and calculated as $V = W^2 \times L / 2$. After the treatment, the heart, liver, spleen, lung, kidney, brain, intestines, and primary and distant tumors were stained with H&E.

Ethics statement

This study was performed with the approval of the Experimental Animal Manage Committee of Sun Yat-Sen University (Approval Nos: SYSU-IACUC-2022-000380, SYSU-IACUC-2023-000337).

Notes and references

1. J. Shen, T. W. Rees, Z. Zhou, S. Yang, L. Ji and H. Chao, *Biomaterials*, 2020, **251**, 120079-120093.
2. B. P. Sullivan, D. J. Salmon and T. J. Meyer, *Inorg. Chem.*, 1978, **17**, 3334-3341.
3. S. Kuang, L. Sun, X. Zhang, X. Liao, T. W. Rees, L. Zeng, Y. Chen, X. Zhang, L. Ji and H. Chao, *Angew. Chem. Int. Ed.*, 2020, **59**, 20697-20703.
4. T. Feng, J. Karges, X. Liao, L. Ji and H. Chao, *Coord. Chem. Rev.*, 2022, **454**, 214325.
5. K. Xiong, C. Ouyang, J. Liu, J. Karges, X. Lin, X. Chen, Y. Chen, J. Wan, L. Ji and H. Chao, *Angew. Chem. Int. Ed.*, 2022, **61**, e202204866.
6. H. Liang, X. Wu, G. Zhao, K. Feng, K. Ni and X. Sun, *J. Am. Chem. Soc.*, 2021, **143**, 15812-15823.

Results and discussion

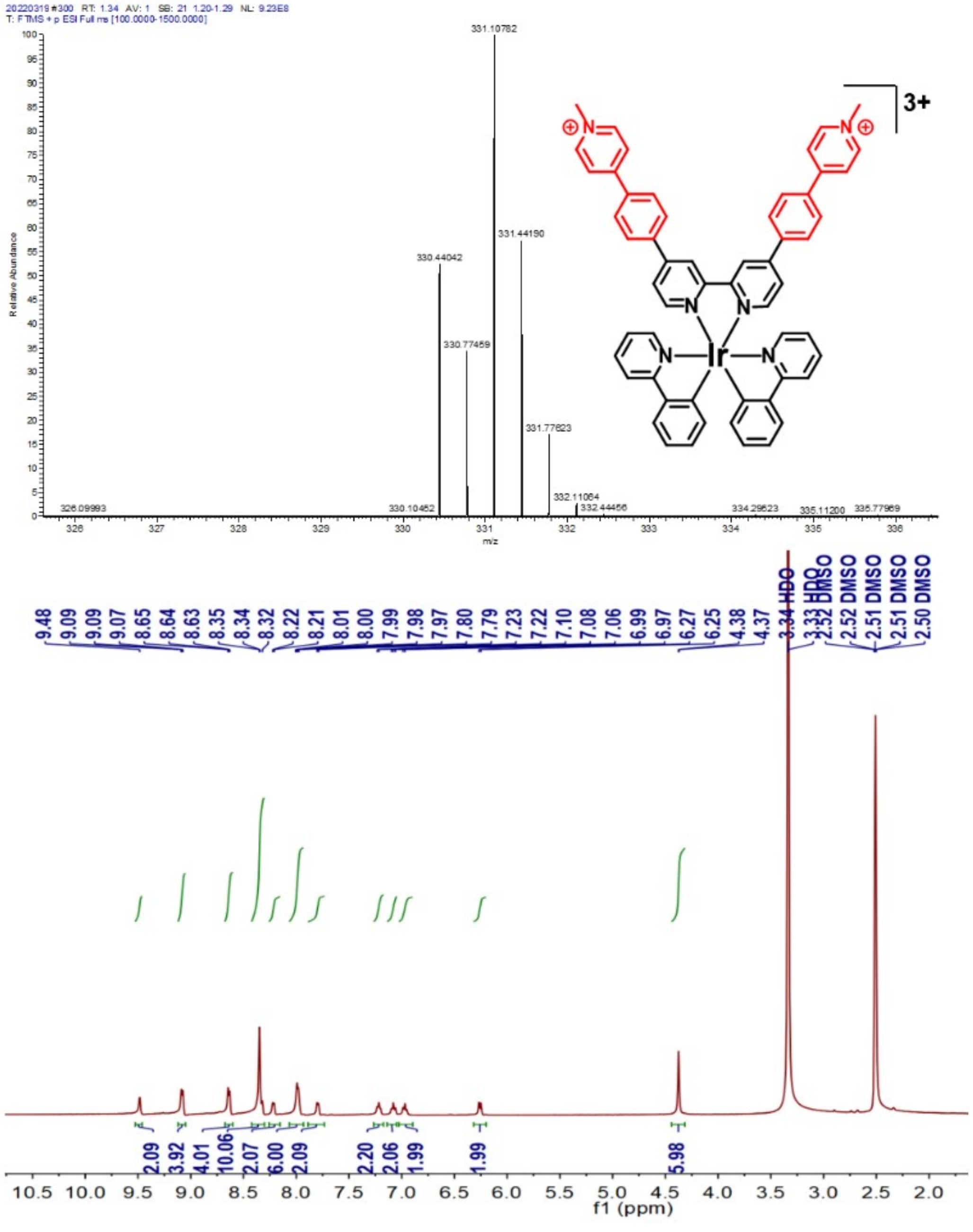

Figure S1. HRMS spectrum and ^{1}H NMR spectrum of **Ir1**.

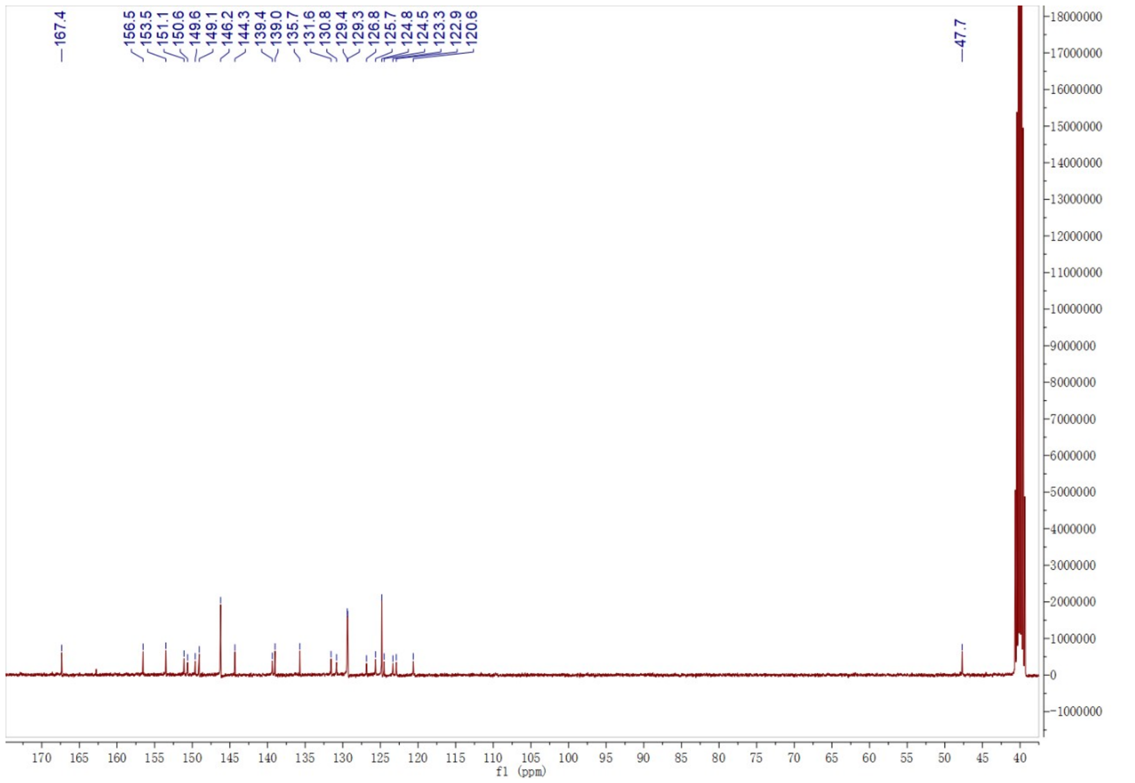

Figure S2. ^{13}C NMR spectrum of **Ir1** in DMSO-d_6**.**

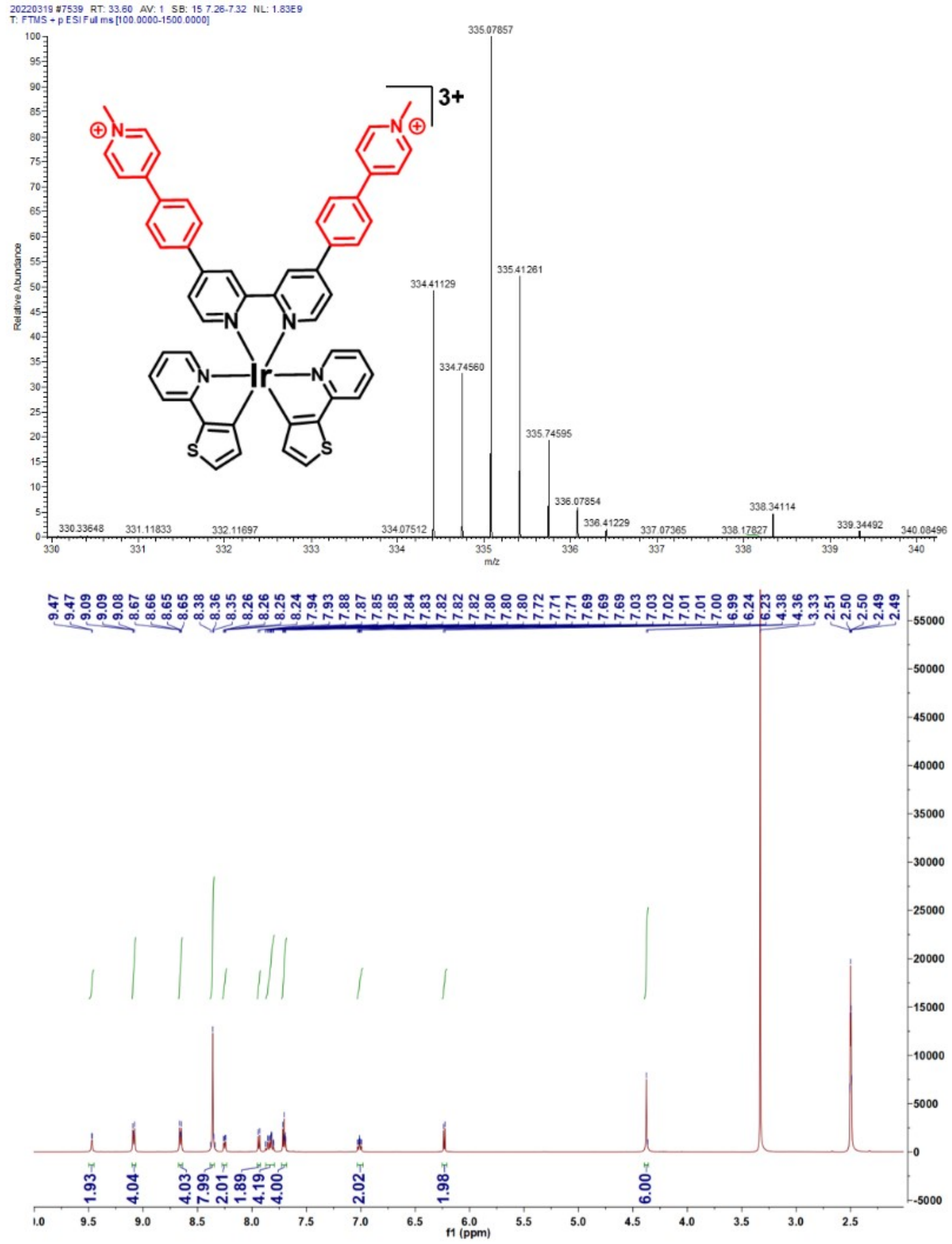

Figure S3. HRMS spectrum and ^{1}H NMR spectrum of **Ir2.**

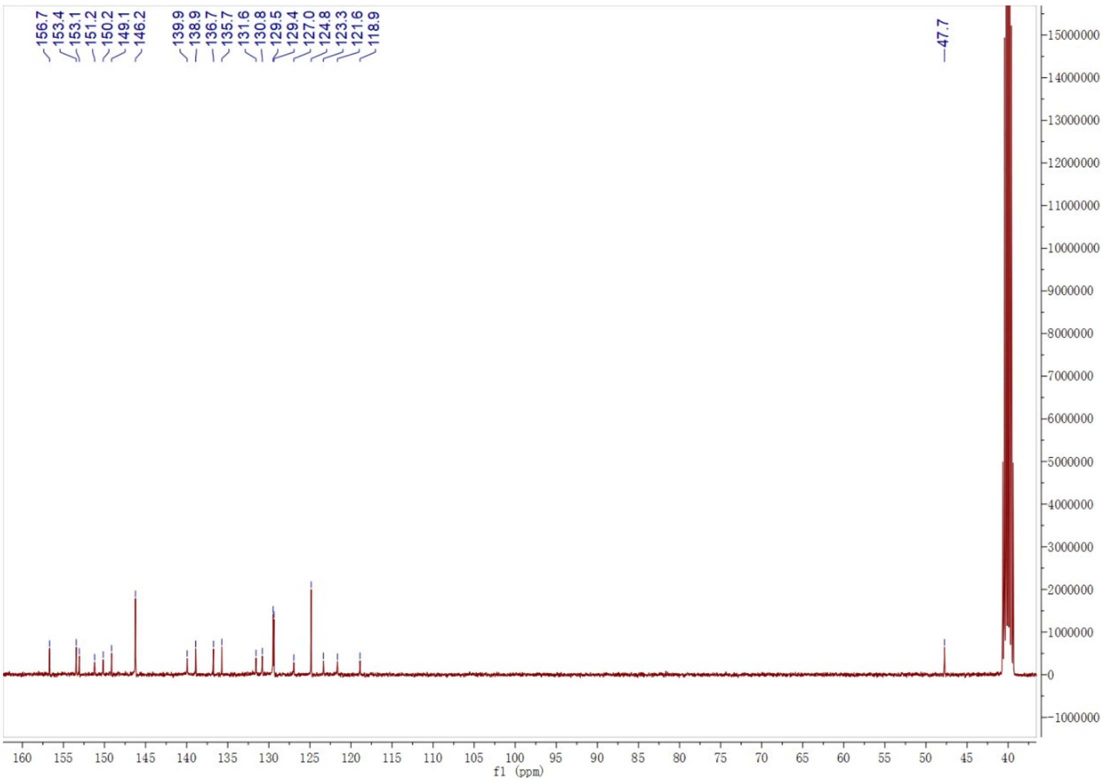

Figure S4. ^{13}C NMR spectrum of **Ir2** in DMSO-d_6.

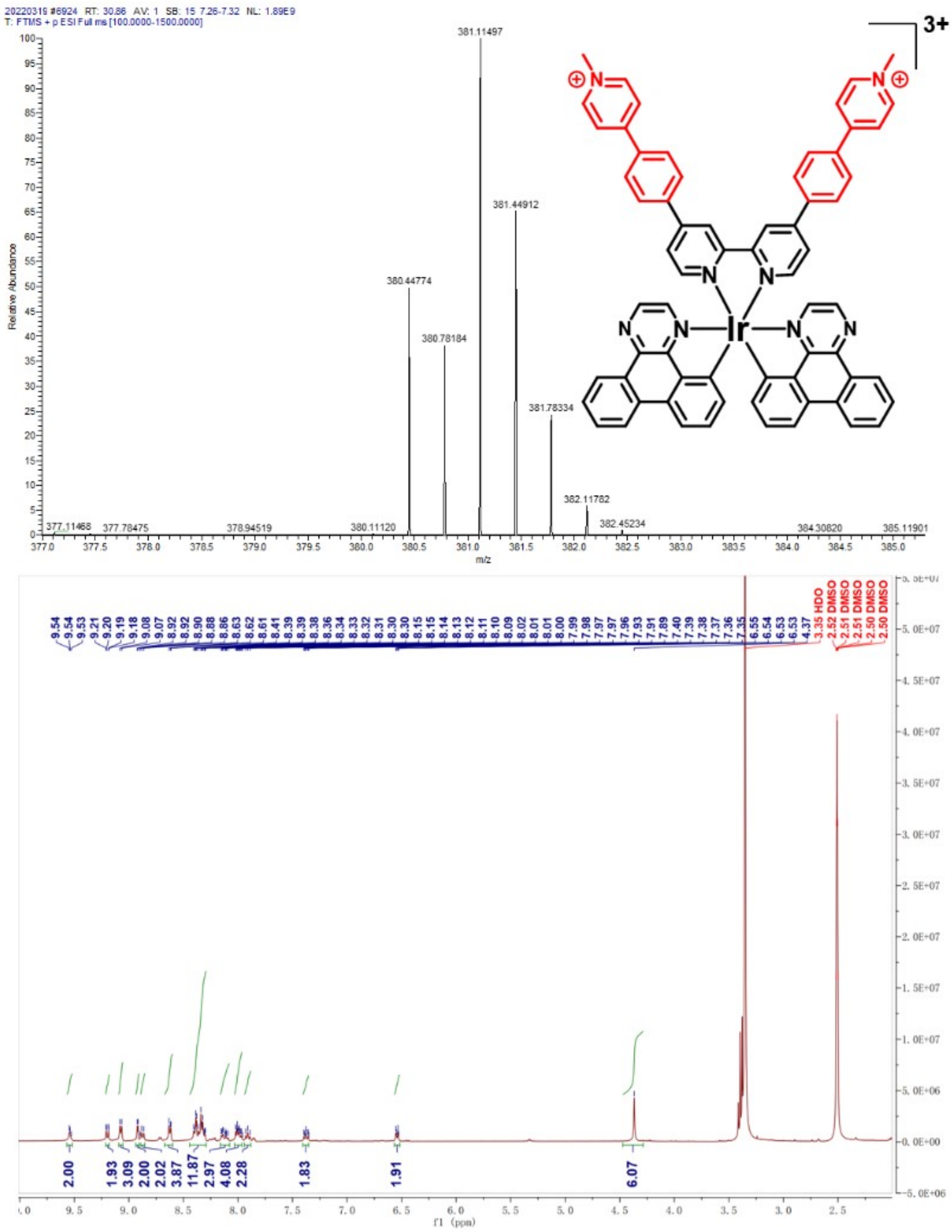

Figure S5. HRMS spectrum and ^{1}H NMR spectrum of **Ir3.**

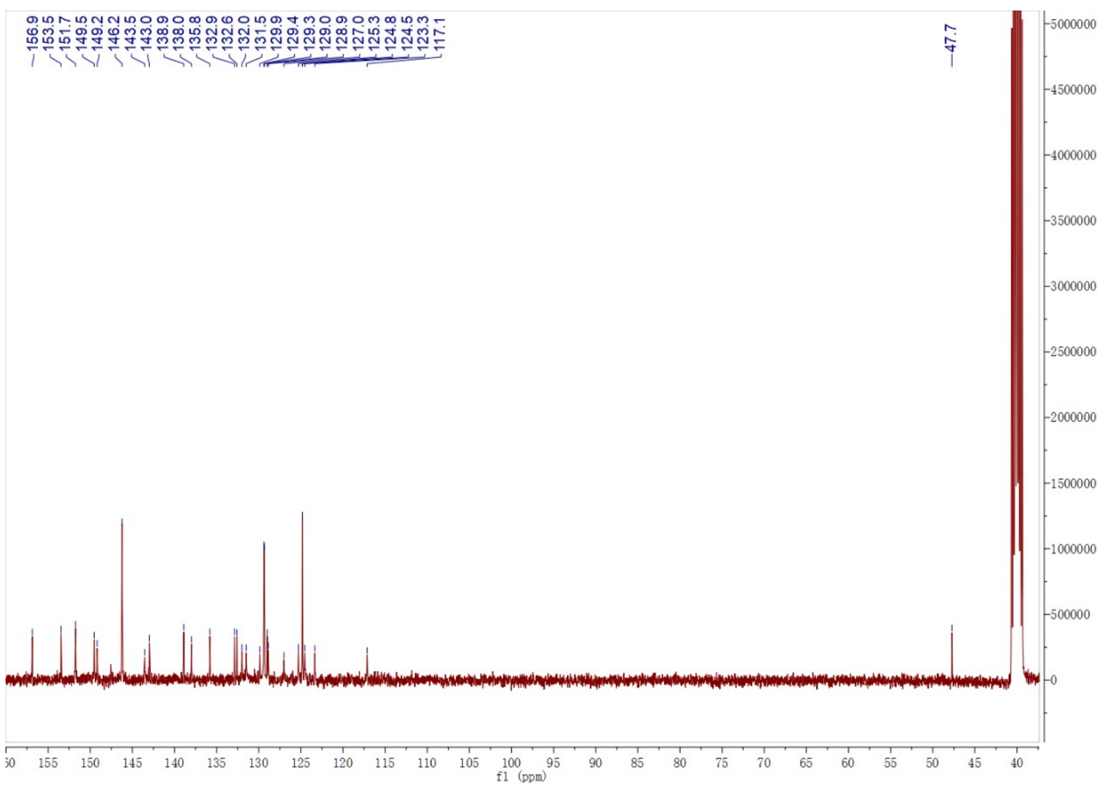

Figure S6. ^{13}C NMR spectrum of **Ir3** in DMSO-d_6.

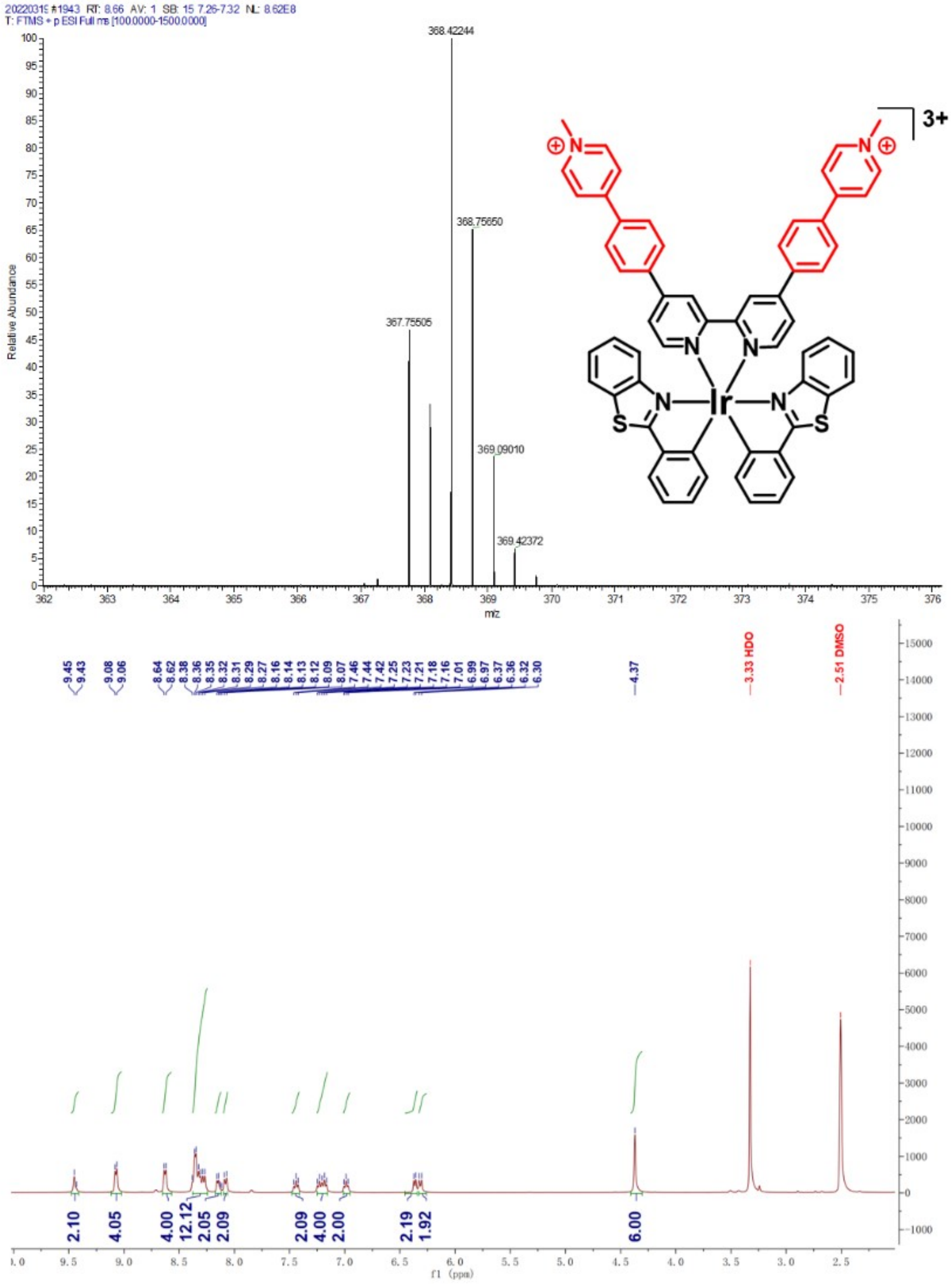

Figure S7. HRMS spectrum and ^{1}H NMR spectrum of **Ir4.**

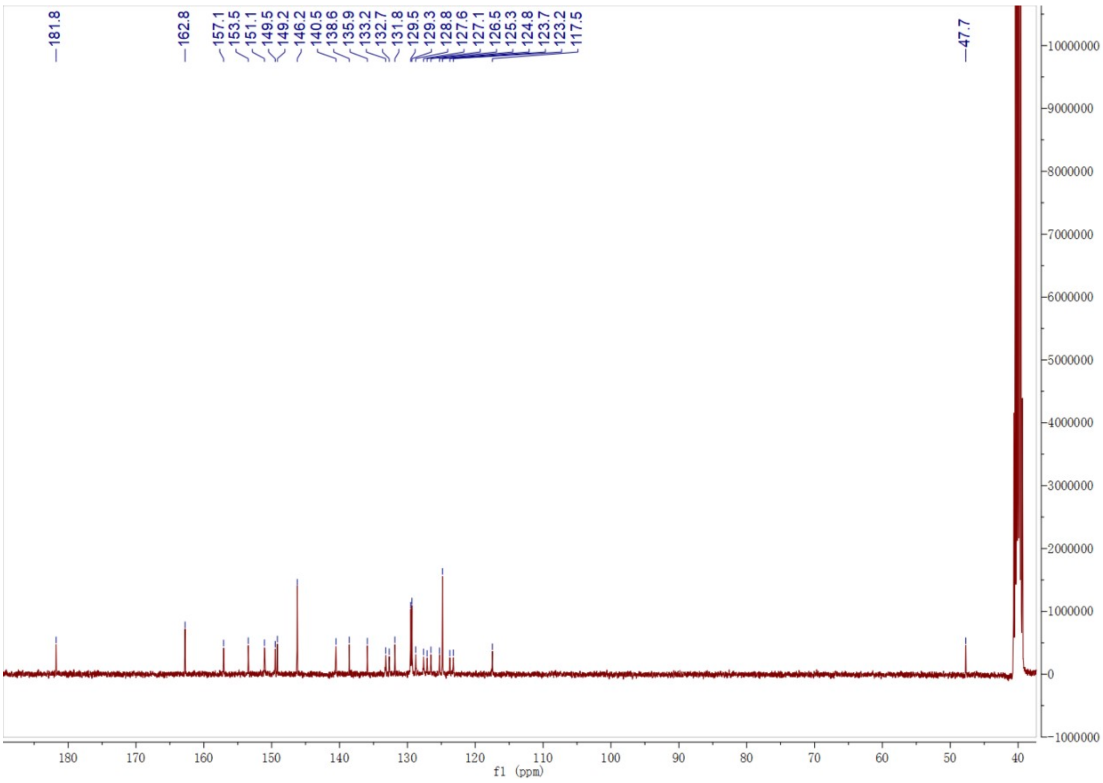

Figure S8. ^{13}C NMR spectrum of **Ir4** in DMSO-d_6**.**

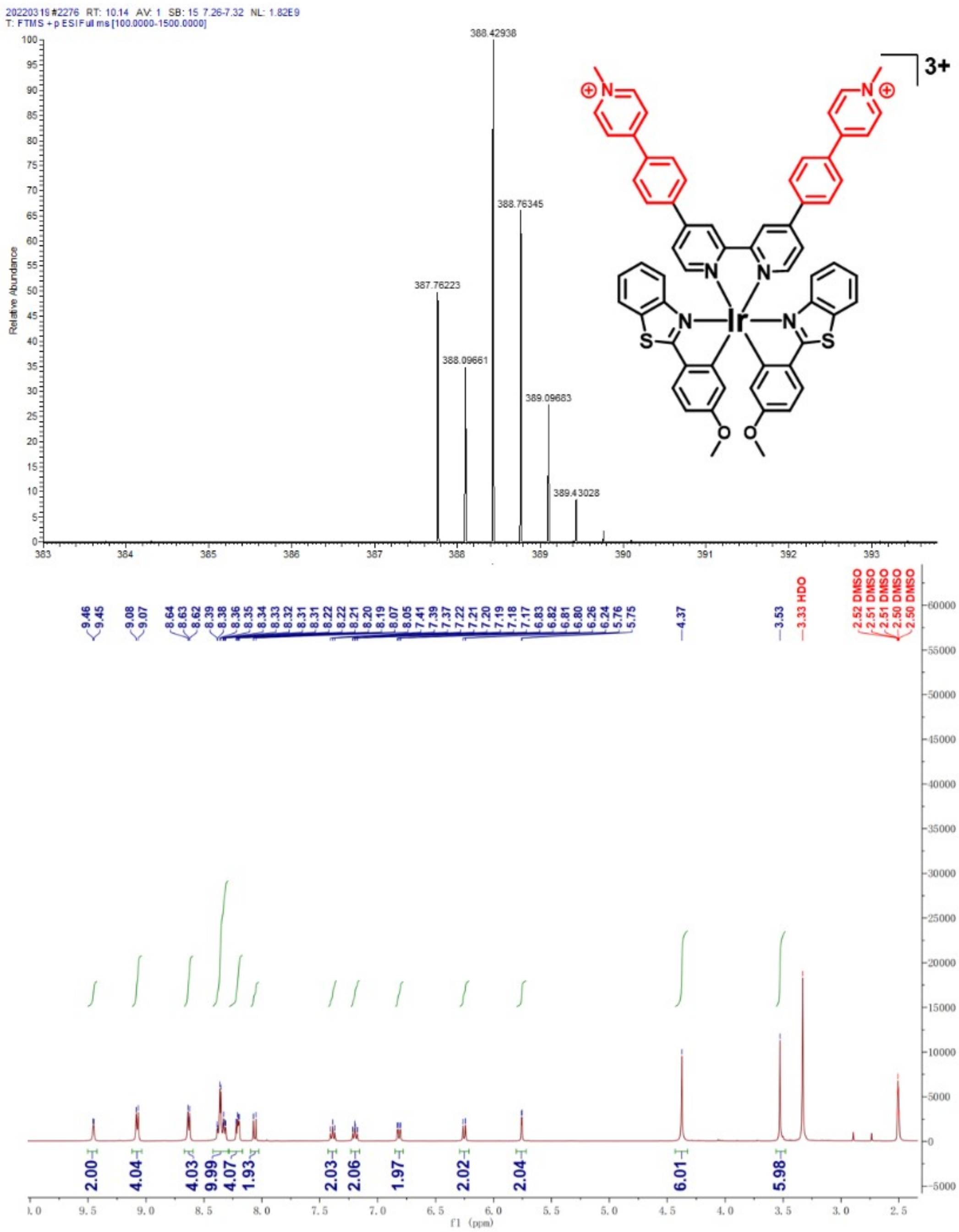

Figure S9. HRMS spectrum and ^{1}H NMR spectrum of **Ir5**.

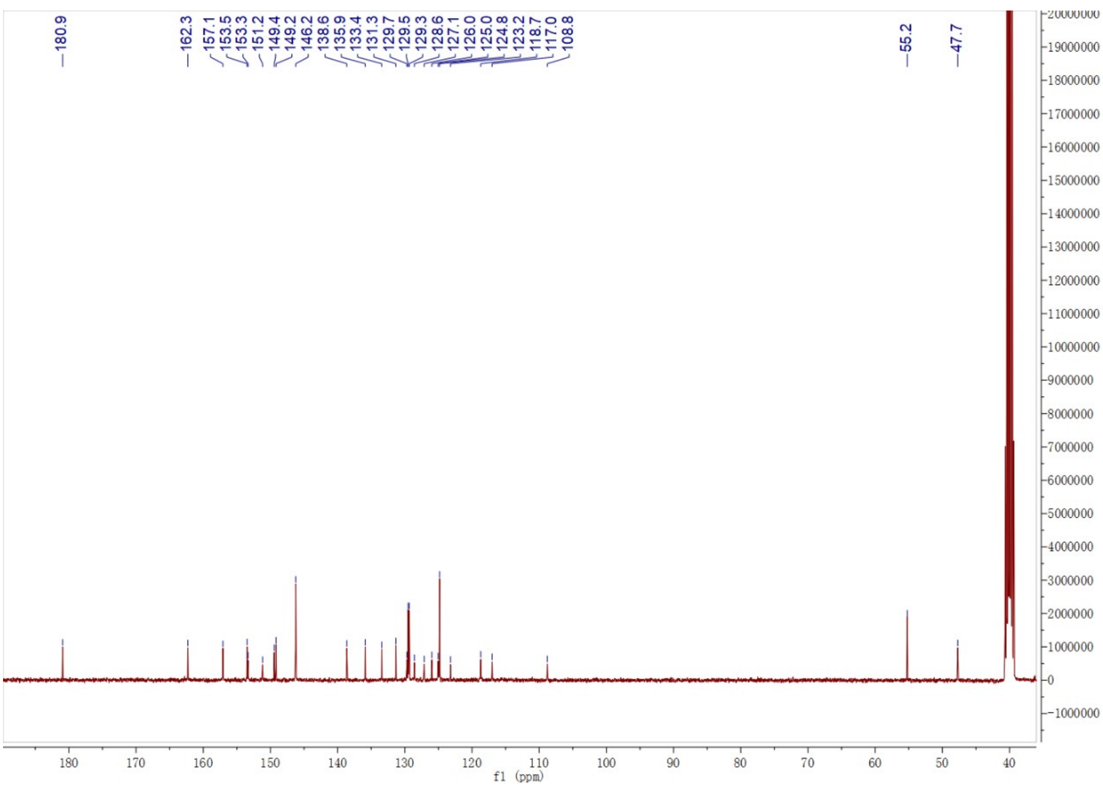

Figure S10. ^{13}C NMR spectrum of **Ir5** in DMSO-d_6.

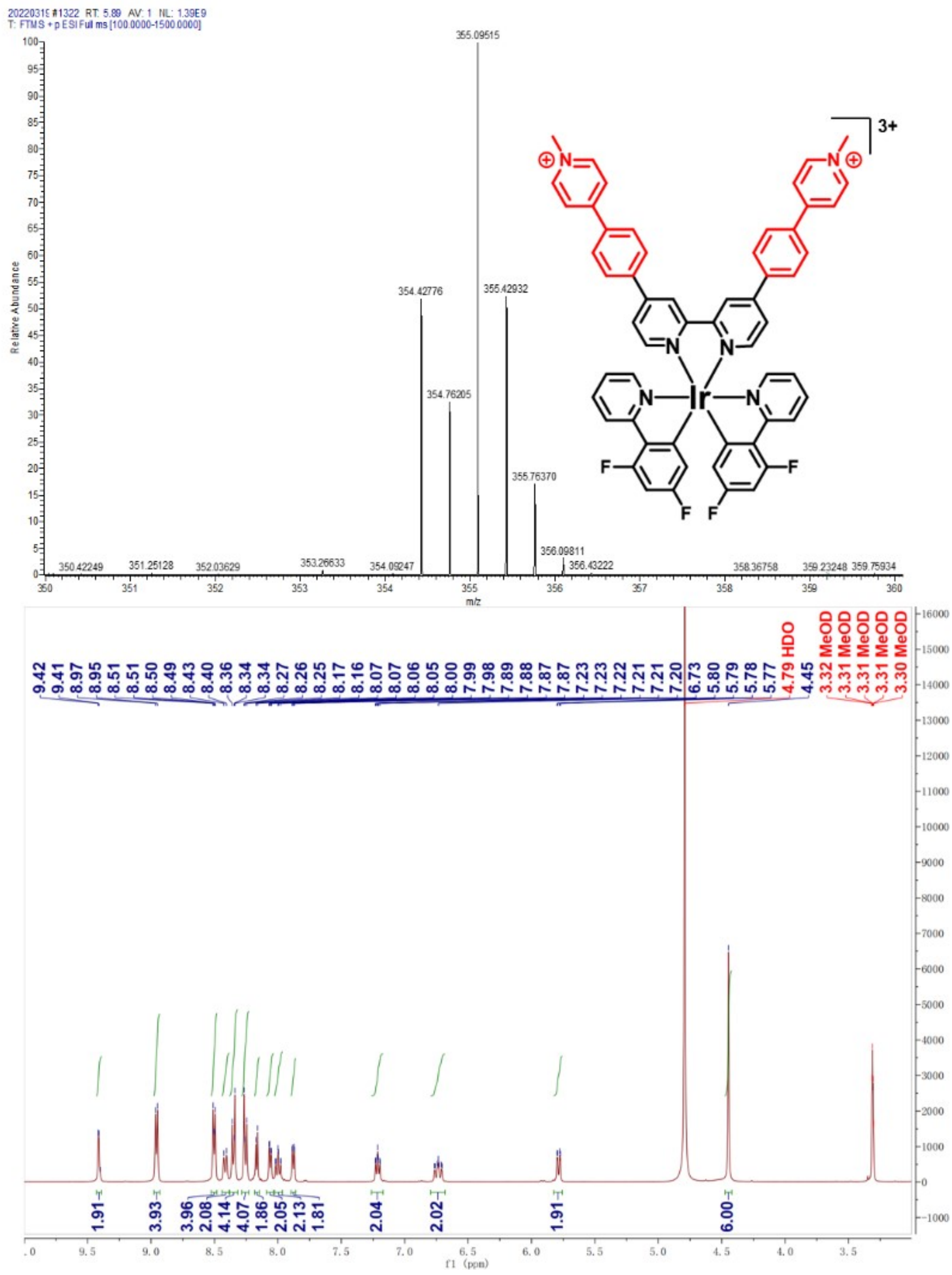

Figure S11. HRMS spectrum and ^{1}H NMR spectrum of **Ir6**.

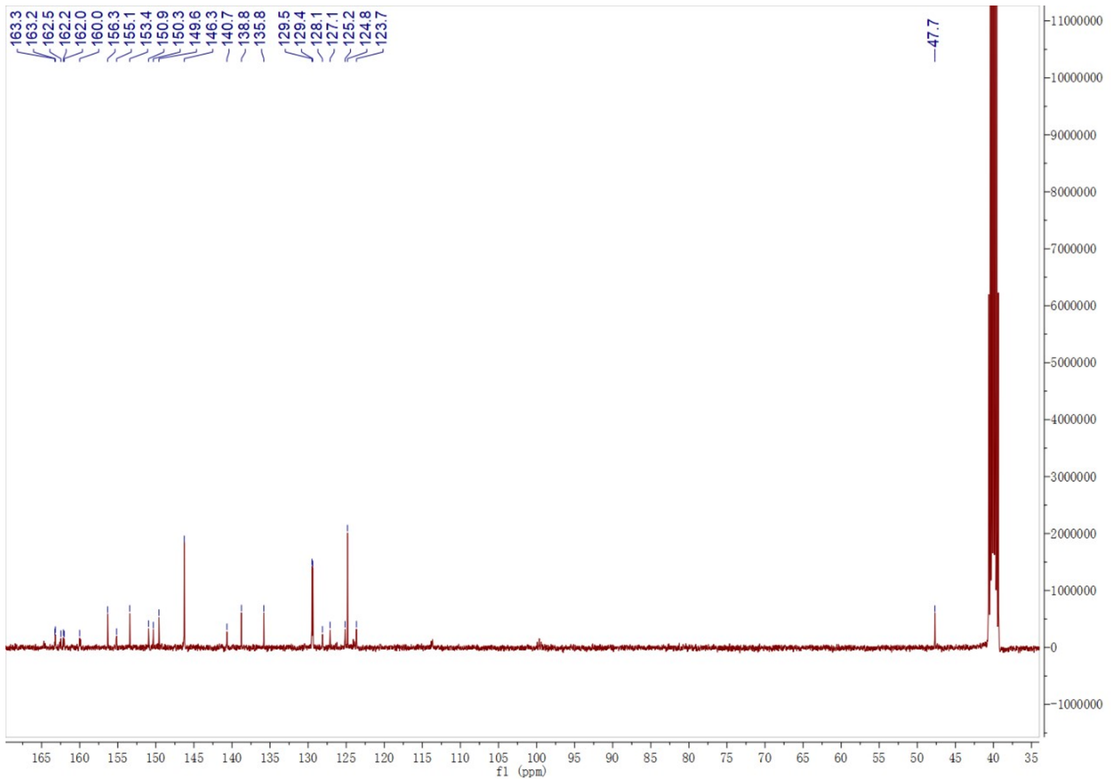

Figure S12. ^{13}C NMR spectrum of **Ir6** in DMSO-d_6.

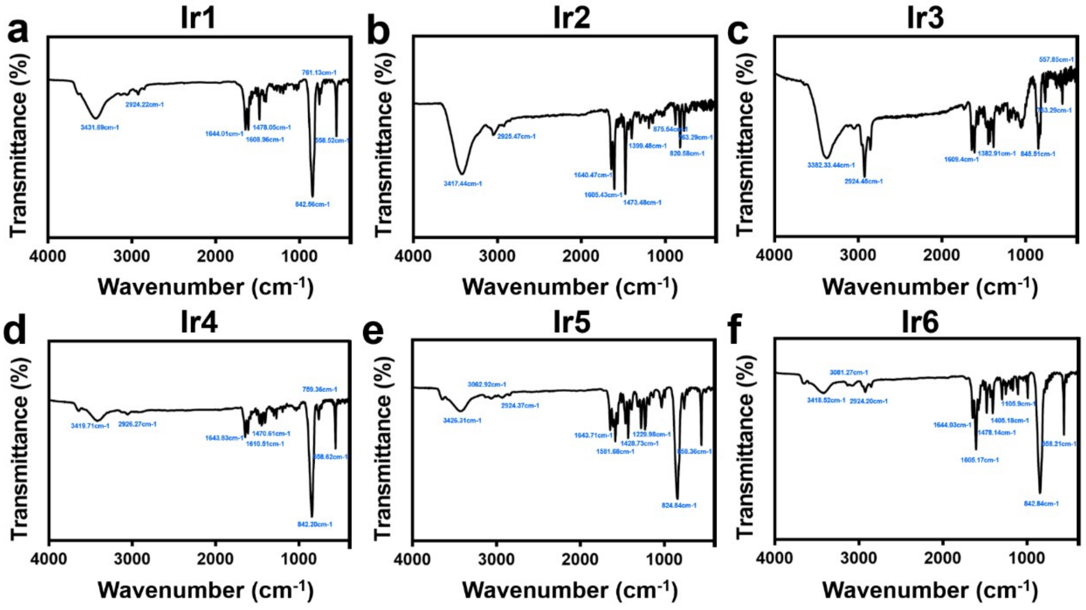

Figure S13. FTIR spectra of **Ir1-Ir6**.

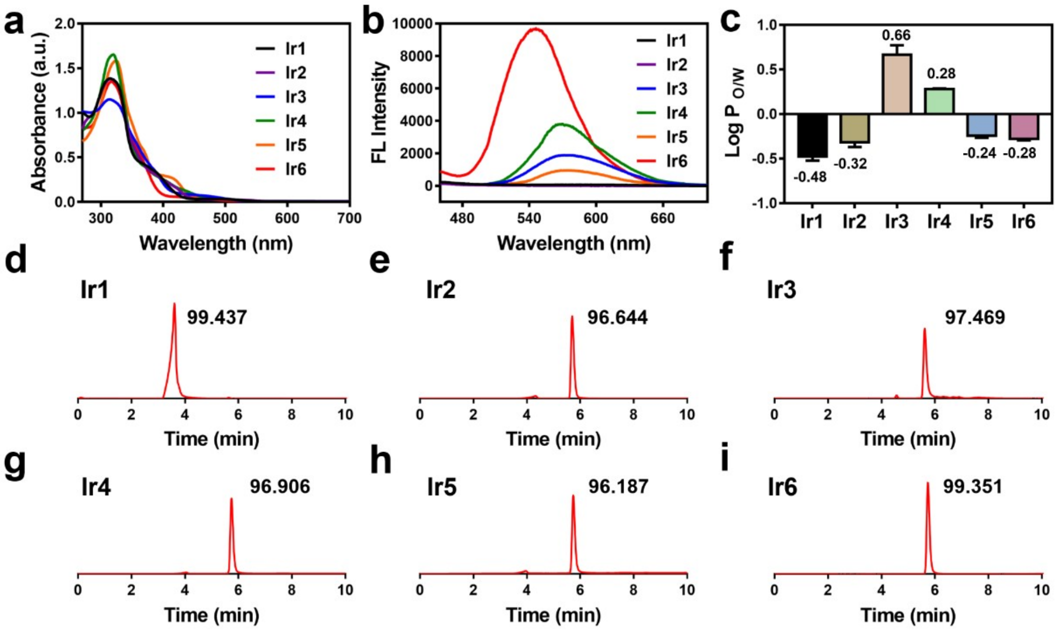

Figure S14. (a) UV-Vis absorption spectra of **Ir1-Ir6** in dichloromethane. (b) Photoluminescence spectra of **Ir1-Ir6** in dichloromethane upon excitation at 405 nm. (c) Octanol/water partition coefficients of **Ir1-Ir6.** (d)-(i) The purity of **Ir1-Ir6** were assessed by HPLC.

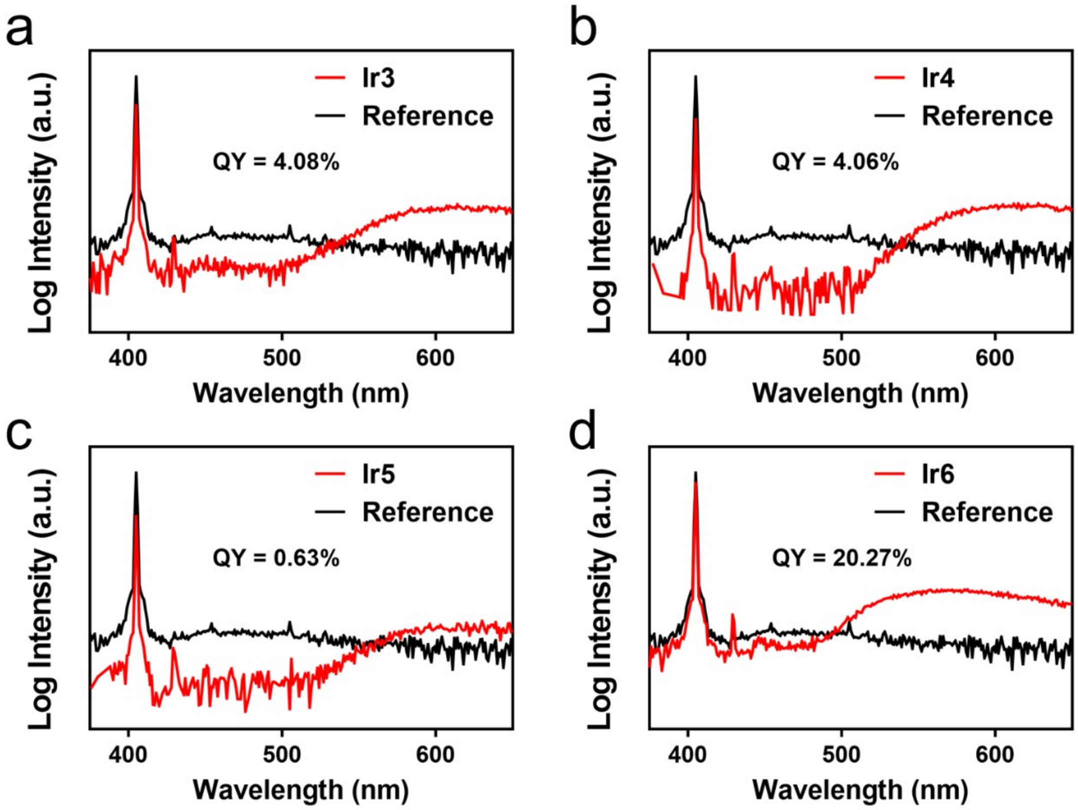

Figure S15. Fluorescence quantum yield determination of **Ir3-Ir6** in DMSO at room temperature, Reference: DMSO.

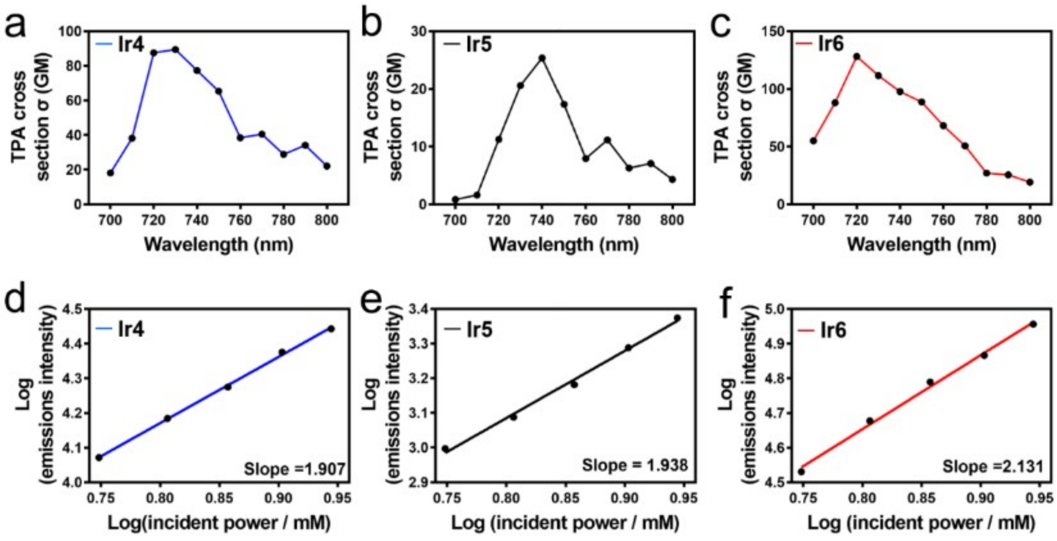

Figure S16. (a-c) Two-photon absorption cross-section spectra of **Ir4-Ir6** in methanol. (d-f) Power dependence relative to the two-photon induced phosphorescence intensity of **Ir4-Ir6** in methanol.

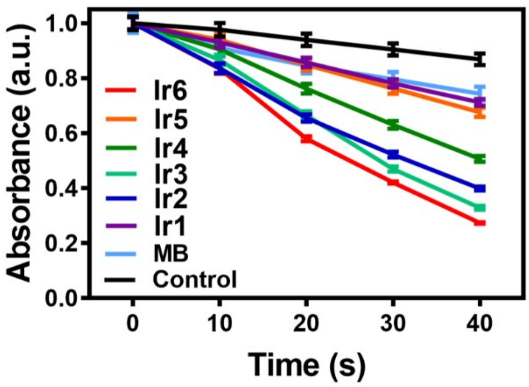

Figure S17. Change of the absorption of the $^1O_2$-specific probe 9,10-Bis(bromomethyl) anthracene (30 μM) at 379 nm upon incubation with **Ir1-Ir6** or methylene blue (MB) as a reference and exposure to irradiation at 405 nm for various time points.

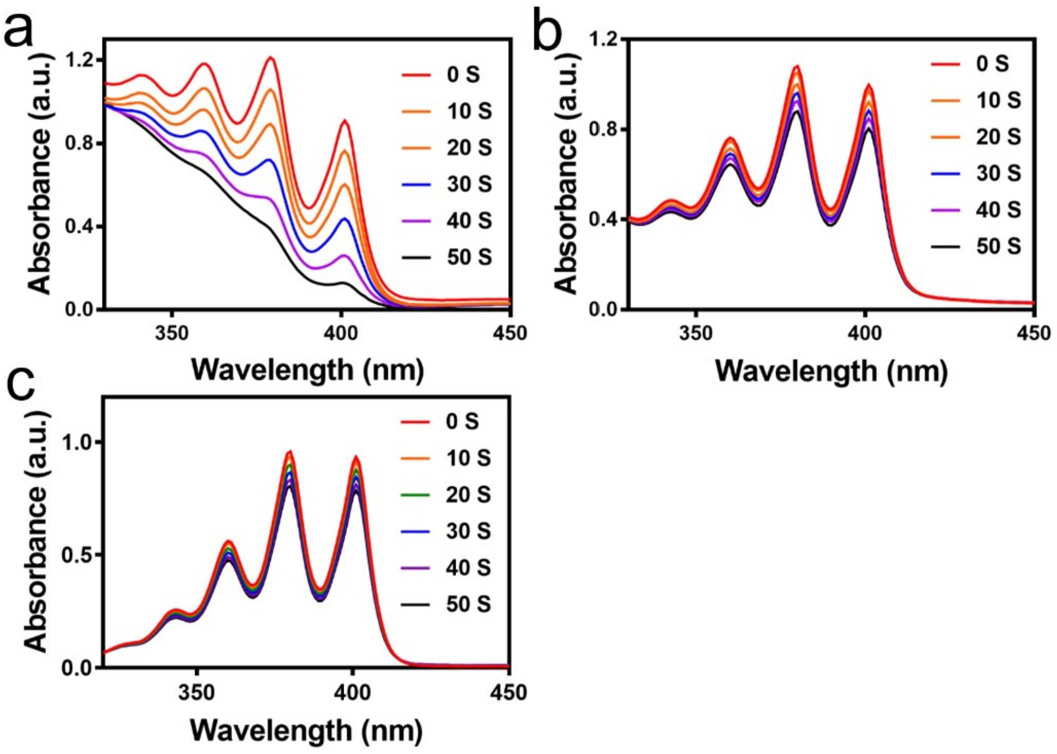

Figure S18. Change in absorption of the $^1O_2$-specific probe 9,10-Bis(bromomethyl) anthracene (30 µM) upon incubation with (a) **Ir6**, (b) MPP+, or (c) without any additions and exposure to two-photon irradiation at 720 nm for various time points.

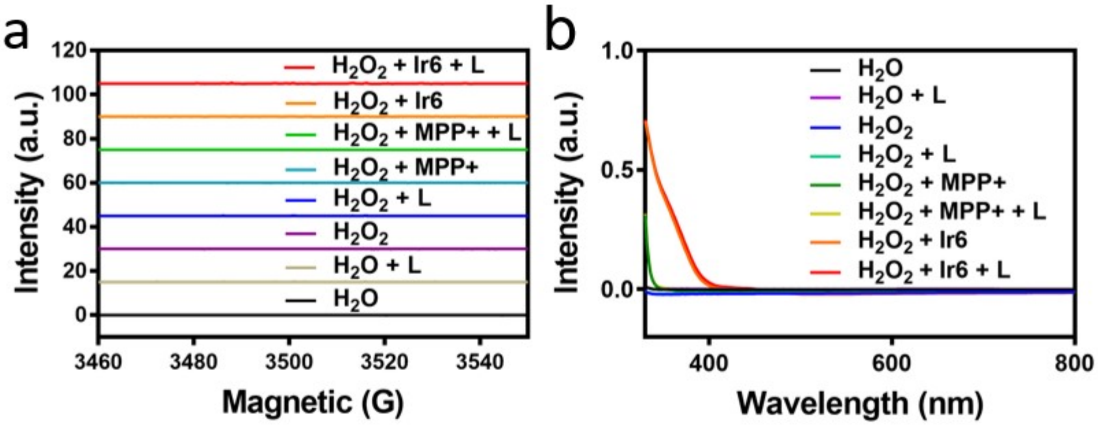

Figure S19. (a) Electron spin resonance spectra of **Ir6** and MPP+ upon incubation with the •OH-specific scavenger 5,5-dimethyl-1-pyrroline-N-oxide in the dark or upon light irradiation (L). (b) Change in the absorption of the 3, 3', 5, 5'-Tetramethylbenzidine upon incubation with **Ir6** or MPP+ in the dark or upon light irradiation (L) for •OH detected.

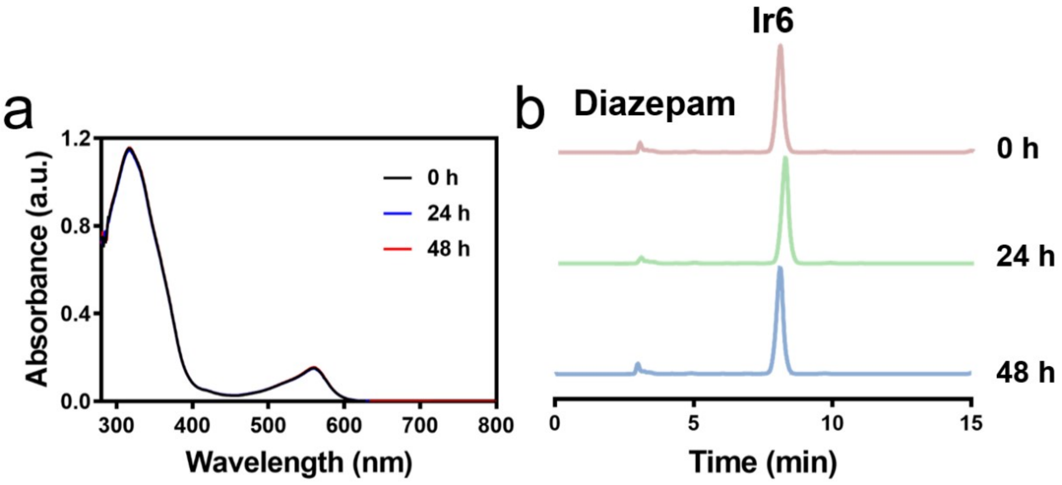

Figure S20. (a) Absorption spectra of **Ir6** upon incubation in fetal bovine serum for various time points. (b) HPLC analysis of **Ir6** incubated in DMEM with 10% FBS for different time points. Diazepam as an internal standard.

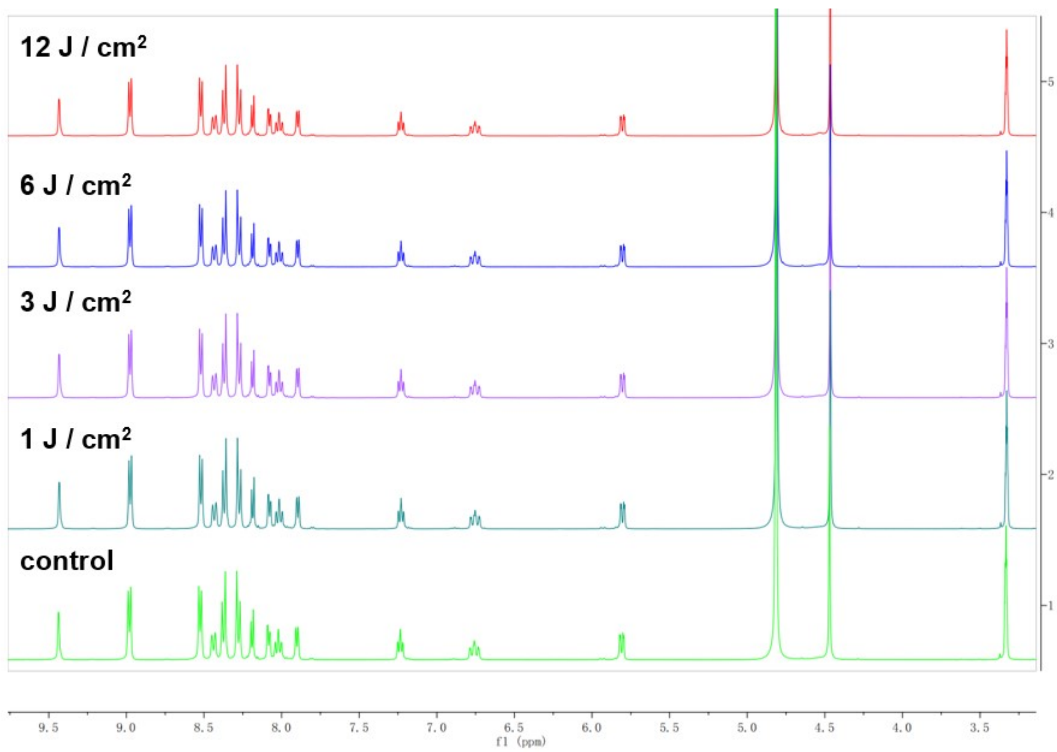

Figure S21. ^{1}H NMR spectra of **Ir6** in methanol upon continuous 720 nm two-photon irradiation with various light doses (1 J/cm^2, 3 J/cm^2, 6 J/cm^2, 12 J/cm^2) in methanol-d_4.

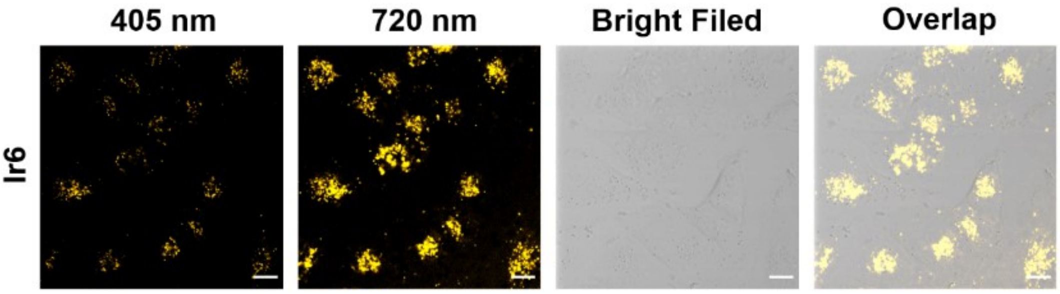

Figure S22. One- and two-photon excited confocal laser scanning microscopy images of A375 cells incubated with **Ir6** (10 μM). Scale bar = 20 μm.

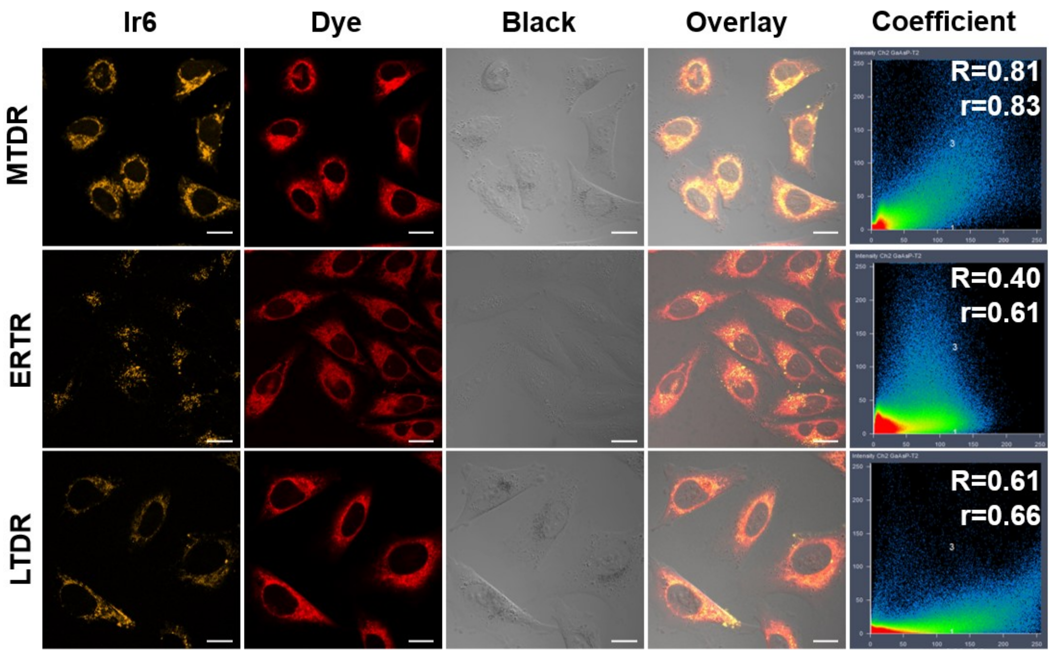

Figure S23. Confocal laser scanning microscopy images of A375 cells incubated with **Ir6** (10 µM) and the Mito-Tracker™ Deep Red (MTDR), ER-Tracker™ Red (ERTR), and the Lyso-Tracker™ Deep Red (LTDR). R: Pearson Correlation Coefficient, r: Manders' Colocalization Coefficient, Scale bar = 20 µm.

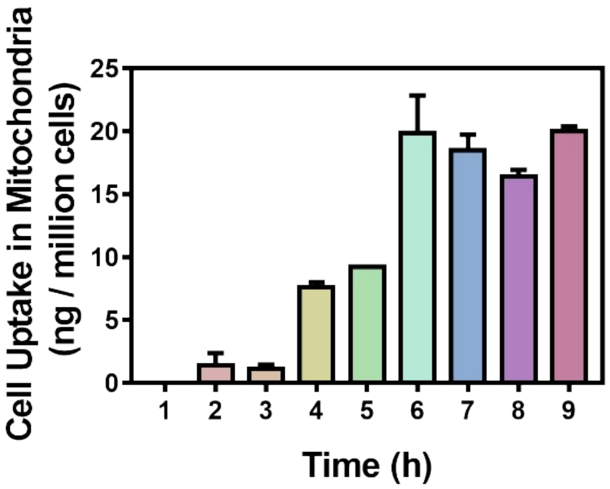

Figure S24. Time-dependent accumulation of **Ir6** in the mitochondria.

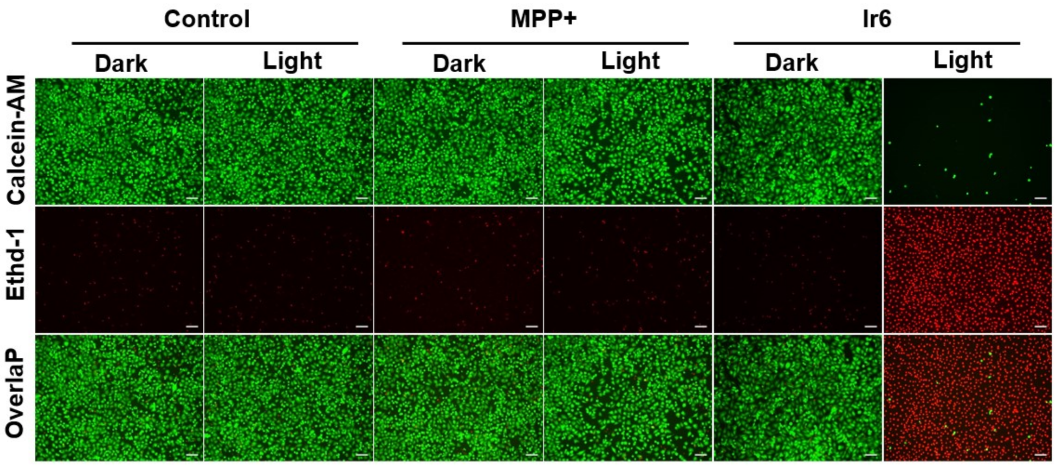

Figure S25. Fluorescent microscopy images of A375 cells treated with MPP+ (100 μM) or **Ir6** (10 μM) in the dark or upon two-photon irradiation (720 nm, 40 mW, 120 s) and incubated with the cell live/dead stain Calcein-AM/EthD-1. Scale bar = 100 μm.

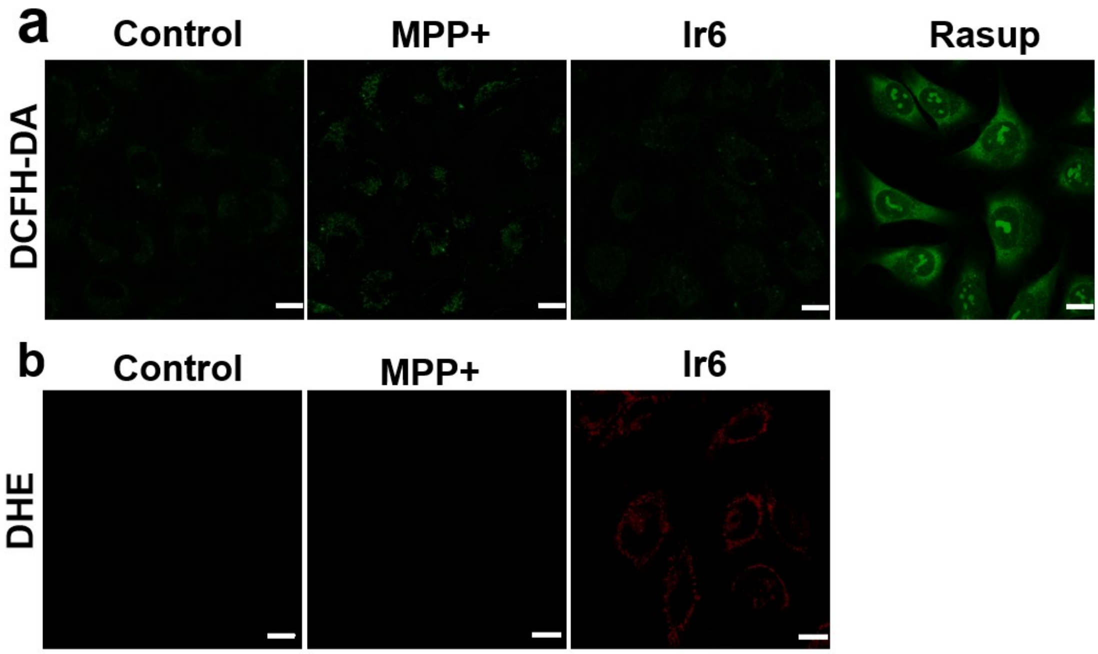

Figure S26. Fluorescence microscopy images of A375 cells incubated with the (a) ROS-specific probe 2,7-dichlorofluorescein diacetate (DCFH-DA). Rosup (10 μM) was used as a positive control. (b) $^{\bullet}O_2^-$-specific probe Dihydroethidium (DHE) and treated with **Ir6** or MPP+ in the dark. Scale bar = 20 μm.

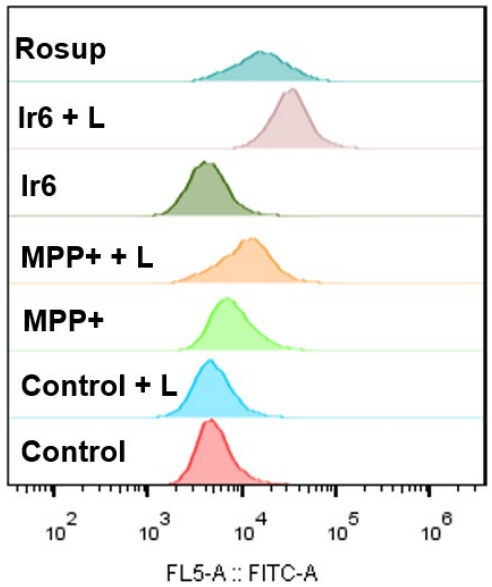

Figure S27. Flow cytometry plots of A375 cells incubated with the ROS-specific probe 2,7-dichlorofluorescein diacetate (DCFH-DA) and treated with **Ir6** (10 μM) or MPP+ (100 μM) in the dark or upon two-photon irradiation (L). L = two-photon laser irradiation at 720 nm, 40 mW, 120 s. Rosup (10 μM) was used as a positive control.

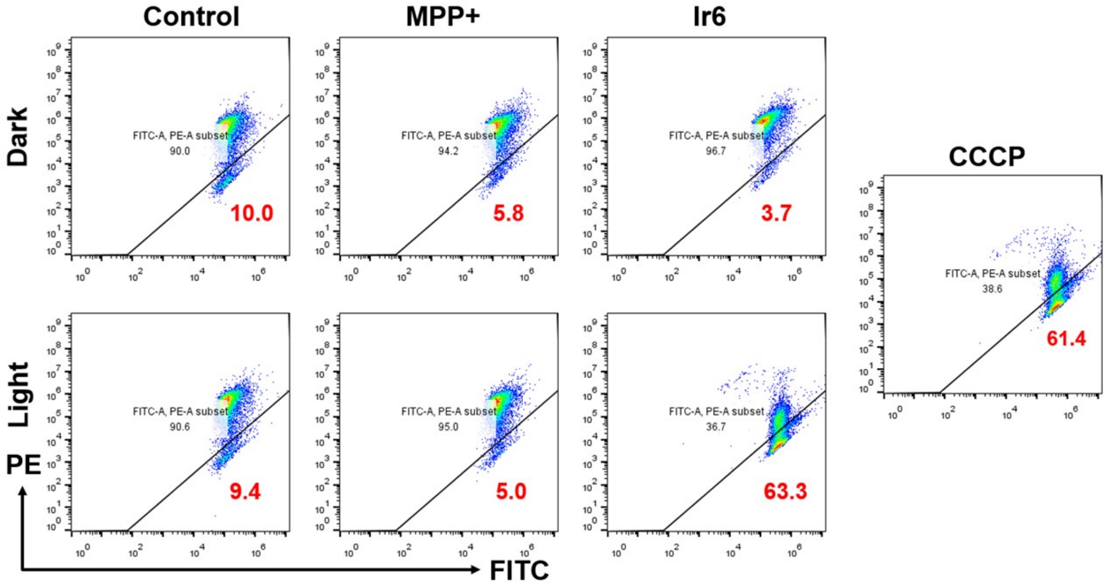

Figure S28. Flow cytometry plots of A375 cells incubated with the mitochondrial membrane potential-specific probe JC-1 and treated with **Ir6** (10 μM) or MPP+ (100 μM) in the dark or upon two-photon irradiation (720 nm, 40 mW, 120 s). Carbonyl cyanide m-chlorophenyl hydrazone (CCCP 20 μM) was used as a positive control.

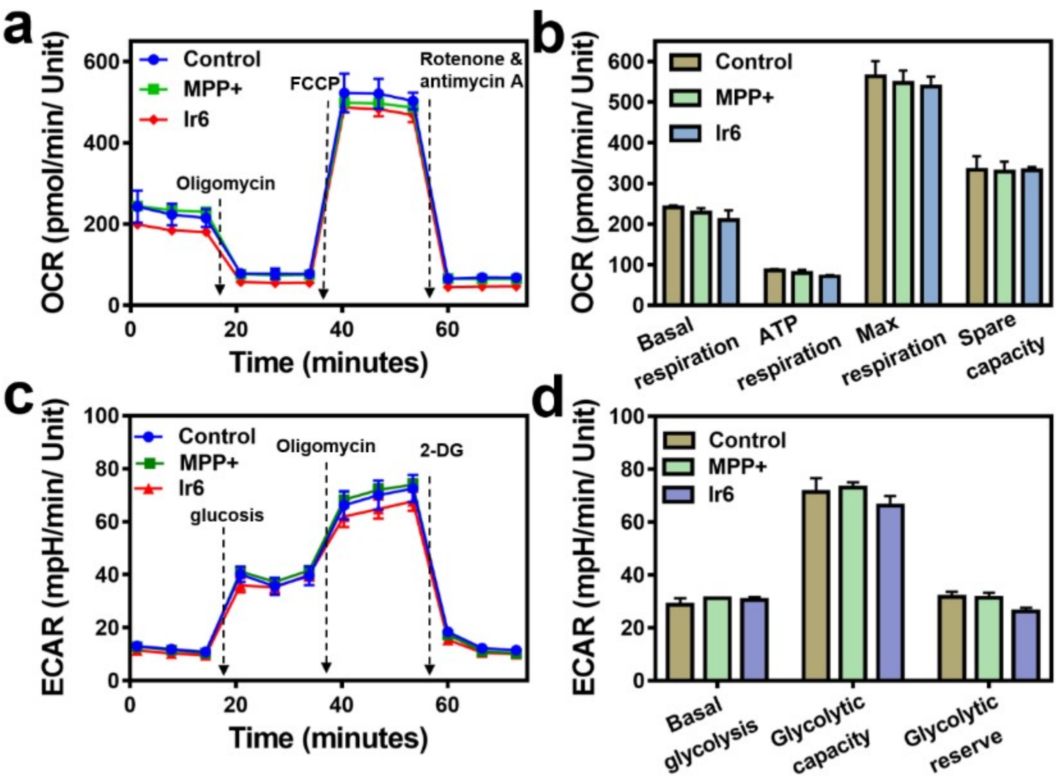

Figure S29. (a) Kinetic profile of the oxygen consumption rate (OCR) of A375 cells treated with **Ir6** and MPP+ in the dark. (b) Quantifying the oxygen consumption rate (OCR) of A375 cells treated with **Ir6** and MPP+ in the dark. (c) Kinetic profile of the extracellular acidification rate (ECAR) profile of A375 cells treated with **Ir6** and MPP+ in the dark. (d) Quantifying the extracellular acidification rate (ECAR) of A375 cells treated with **Ir6** and MPP+ in the dark. Concentration: **Ir6** (10 µM), MPP+ (100 µM). Error bars = standard deviation, n = 3.

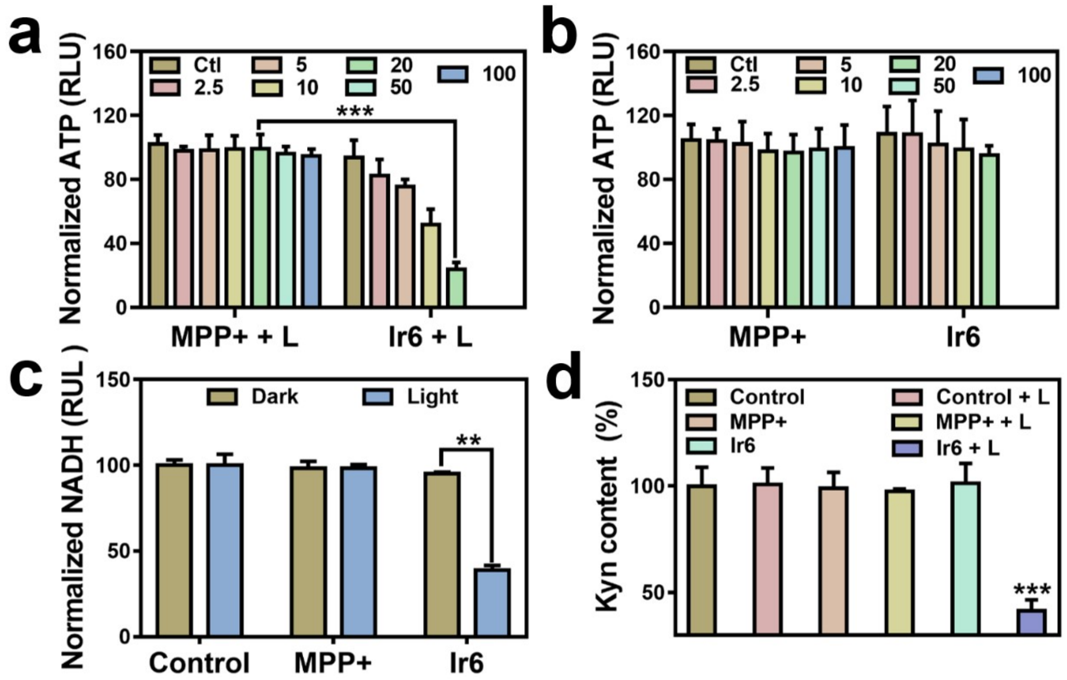

Figure S30. (a) Determination of the intracellular ATP levels of A375 cells treated with **Ir6** (10 μM) and MPP+ (100 μM) and two-photon irradiation (L). (b) Determination of the intracellular ATP levels of A375 cells treated with **Ir6** (10 μM) and MPP+ (100 μM) in the dark. (c) Determination of the intracellular nicotinamide adenine dinucleotide hydrogen (NADH) levels of A375 cells treated with **Ir6** (10 μM) and MPP+ (100 μM) and in the dark or upon two-photon irradiation (L). (d) Determination of the intracellular kynurenine (Kyn) levels of A375 cells treated with **Ir6** (10 μM) and MPP+ (100 μM) and in the dark or upon two-photon irradiation (L). L = two-photon laser irradiation at 720 nm, 40 mW, 120 s. Error bars = standard deviation. n = 3, **p<0.01, ***p<0.001.

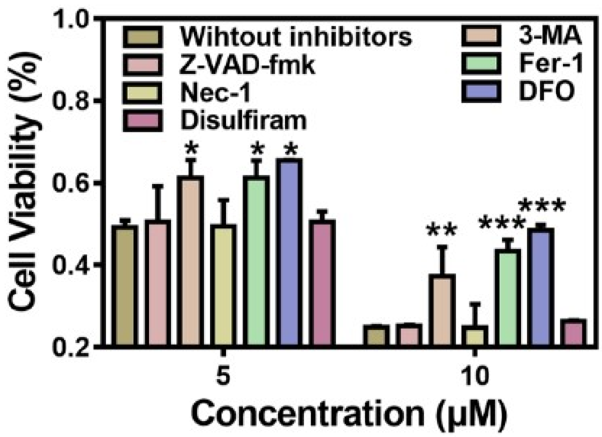

Figure S31. Cell viabilities of A375 cells that were pre-incubated with apoptosis (z-VAD-fmk, 20 μM), necrosis (necrostatin-1, Nec-1, 50 μM), autophagy (3-methyladenine, 3-MA, 100 μM), ferroptosis (ferrostatin-1, Fer-1, 50 μM and deferoxamine, DFO, 100 μM), and pyroptosis (Disulfiram, 4 μM) or without inhibitors and treated with **Ir6** upon two-photon irradiation (720 nm, 40 mW, 120 s). Error bars = standard deviation, n = 3. *p<0.05, **p<0.01, ***p<0.001.

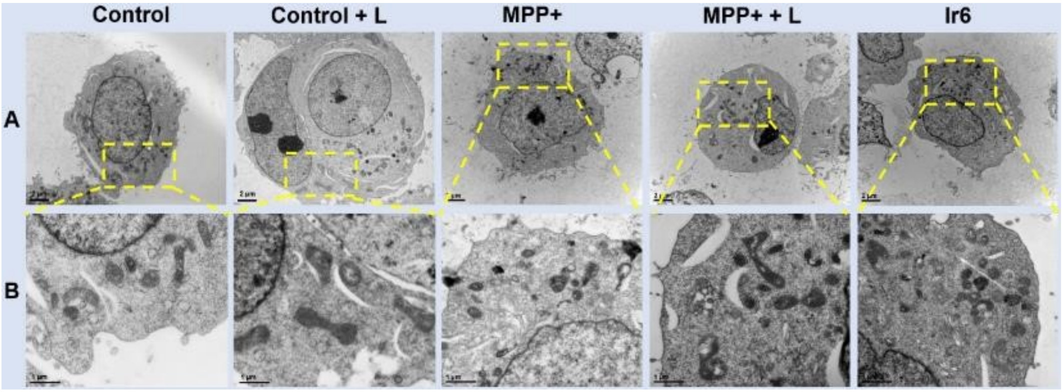

Figure S32. (A) Representative TEM images of the autophagosomes of A375 cells. Incubation with control, control + L, MPP+, MPP+ + L and **Ir6**. Scale bar=2 µm. (B) Partially enlarged view from A. Scale bar = 1 µm. Concentration: **Ir6** (10 µM), MPP+ (100 µM). L = two-photon laser irradiation at 720 nm, 40 mW, 120 s.

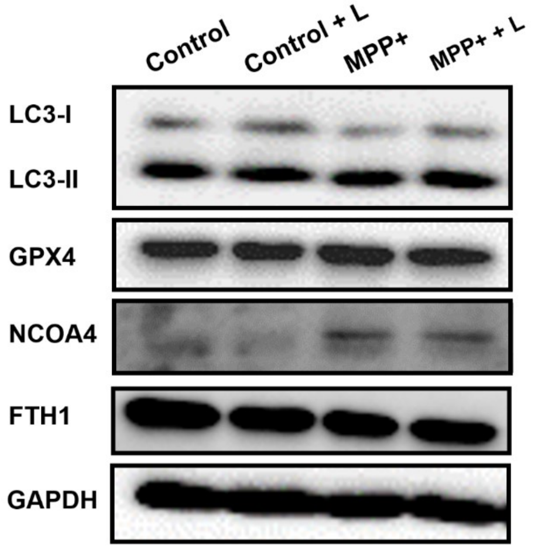

Figure S33. Western Blot analysis of LC3-I, LC3-II, GPX4, NCOA4, and FTH1 expression levels in A375 cells upon treatment with MPP+ and irradiation (L). Concentration: MPP+ (100 µM). L = two-photon laser irradiation at 720 nm, 40 mW, 120 s.

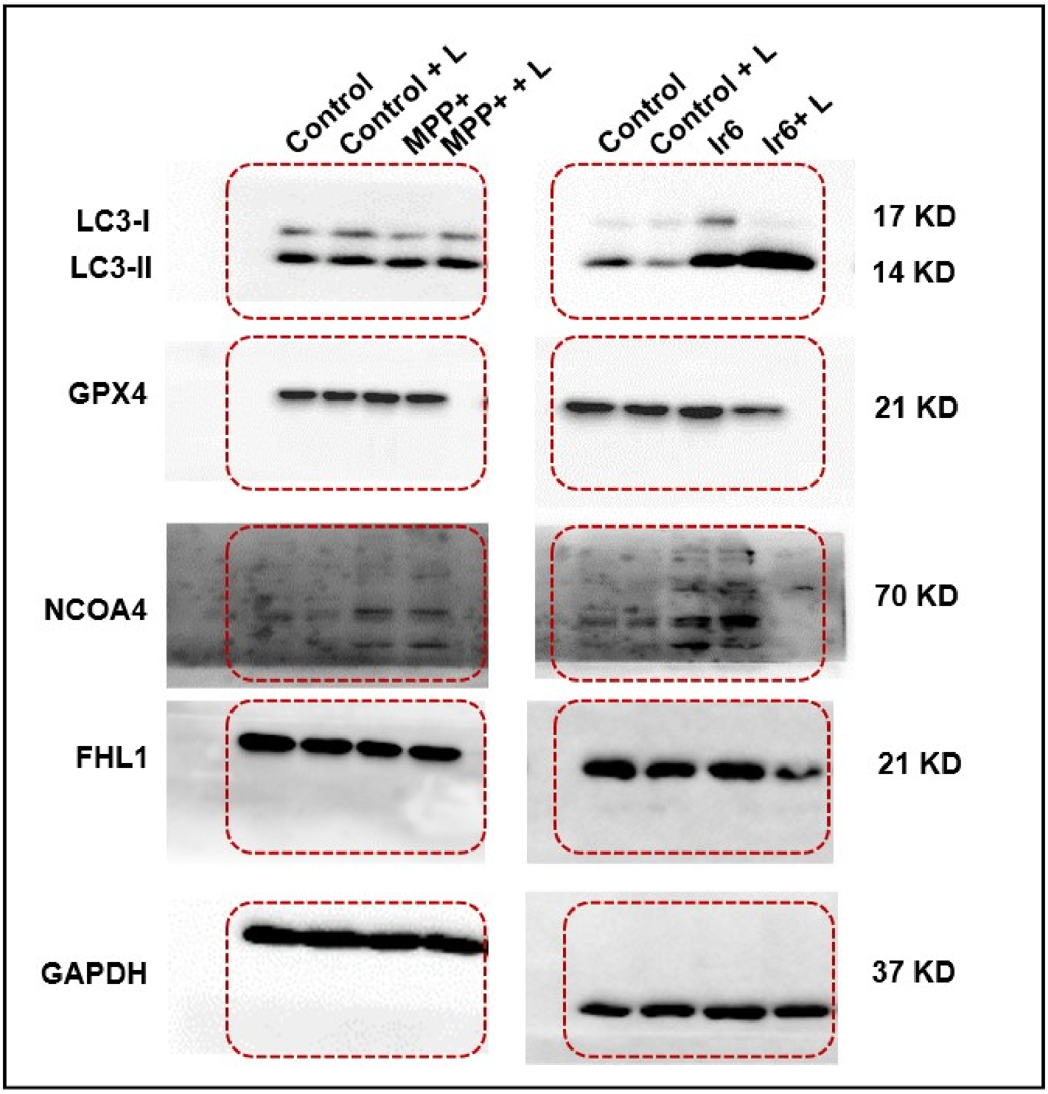

Figure S34. The uncropped Western Blot for **Figure 3b** and **Figure S33.**

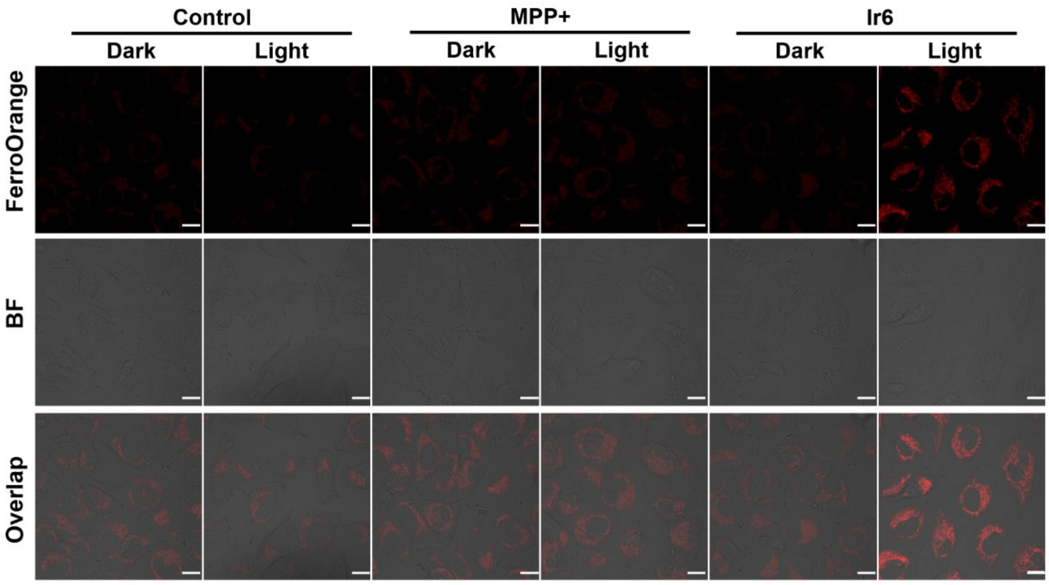

Figure S35. Confocal laser scanning microscopy images of A375 cells incubated with the Fe(II)-specific fluorescence probe FerroOrange and treated with MPP+ or **Ir6** in the dark or upon irradiation. Concentration: **Ir6** (10 μM), MPP+ (100 μM). Light = two-photon laser irradiation at 720 nm, 40 mW, 120 s. Scale bars = 20 μm.

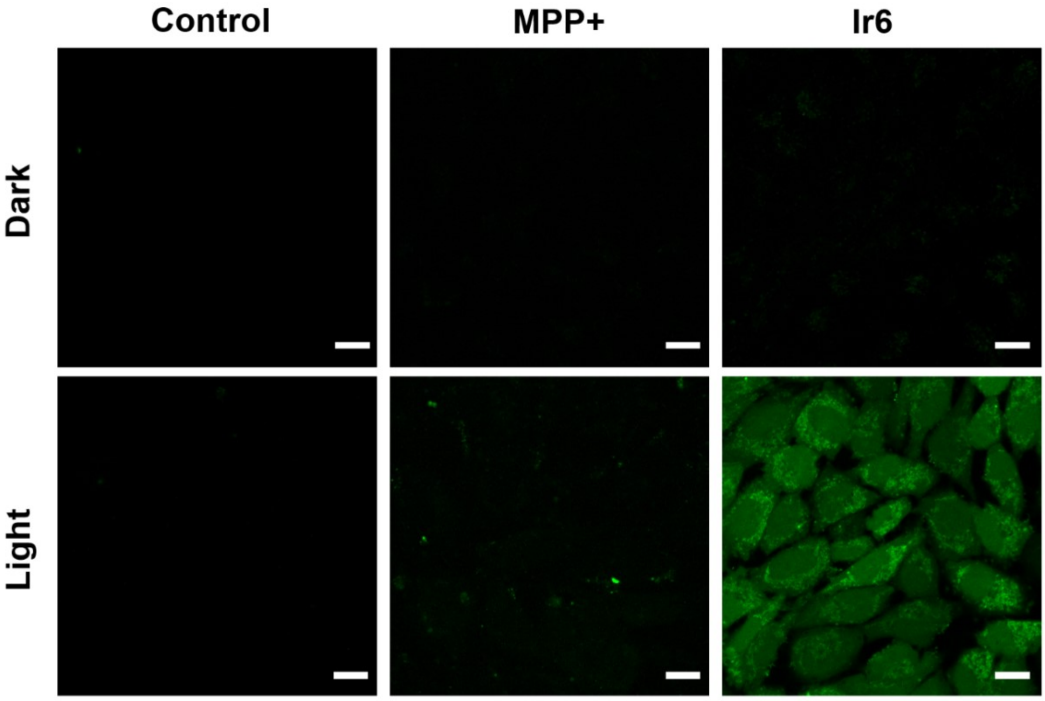

Figure S36. Fluorescence microscopy images of A375 cells incubated with the H_2O_2-specific probe and treated with **Ir6** or MPP+ in the dark or upon irradiation (L). L = two-photon laser irradiation at 720 nm, 40 mW, 120 s. Concentration: **Ir6** (10 µM), MPP+ (100 µM). Scale bars = 20 µm.

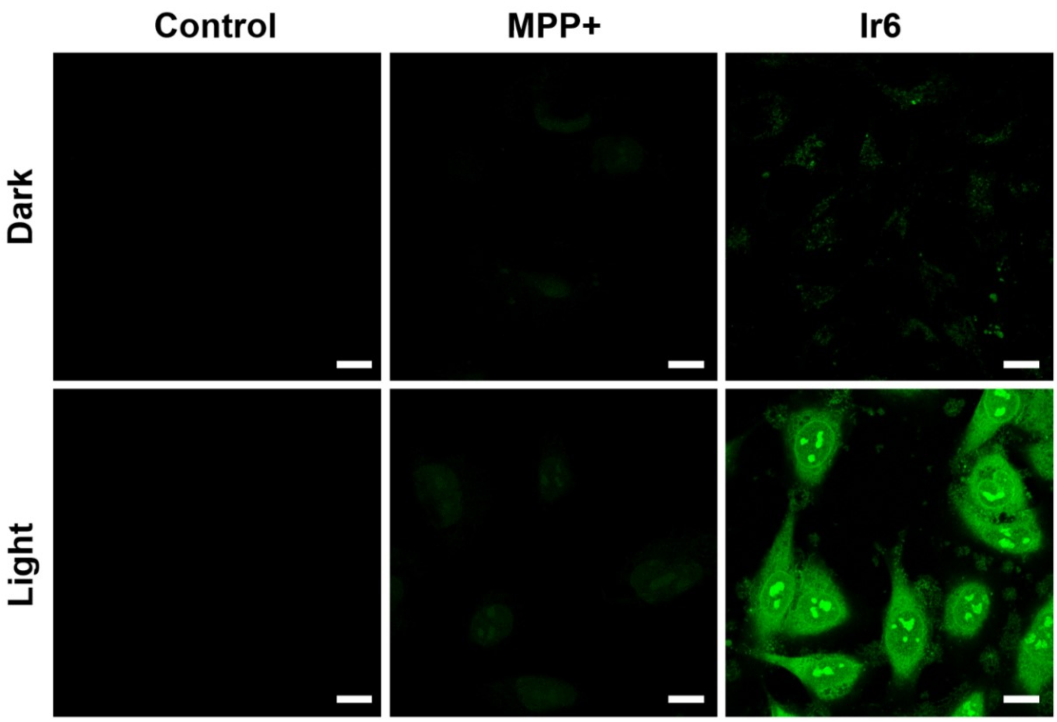

Figure S37. Fluorescence microscopy images of A375 cells incubated with the •OH-specific probe and treated with **Ir6** or MPP+ in the dark or upon irradiation (L). L = two-photon laser irradiation at 720 nm, 40 mW, 120 s. Concentration: **Ir6** (10 μM), MPP+ (100 μM). Scale bars = 20 μm.

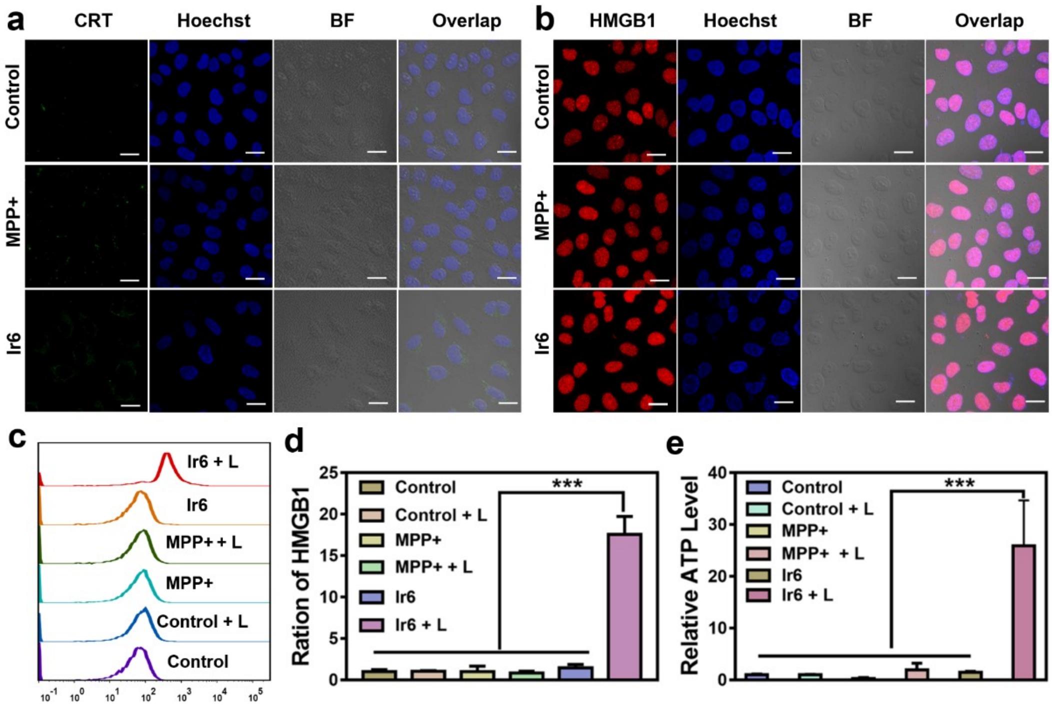

Figure S38. Monitoring of immunogenic cell death-specific hallmarks in A375 cells upon treatment with **Ir6** in the dark or exposure to irradiation (L). (a) Immunofluorescence CLSM of A375 cells incubated with the calreticulin-specific antibody and treated with MPP+ or **Ir6** in the dark. Scale bar = 20 µm. (b) Immunofluorescence CLSM of A375 cells incubated with the human nuclear high mobility group box 1 protein-specific antibody and treated with MPP+ or **Ir6** in the dark. Scale bar = 20 µm. (c) Flow cytometry plots of the translocation of calreticulin in A375 upon treatment with **Ir6** in the dark or exposure to irradiation (L). (d) Enzyme-linked immunosorbent assay analysis of the human nuclear high mobility group box 1 protein migration upon treatment with **Ir6** in the dark or exposure to irradiation (L). (e) Extracellular release of ATP upon treatment with **Ir6** in the dark or exposure to irradiation (L). L = two-photon laser irradiation at 720 nm, 40 mW, 120 s. Concentration: **Ir6** (10 µM), MPP+ (100 µM). Error bars = standard deviation, n = 3. ***p<0.001.

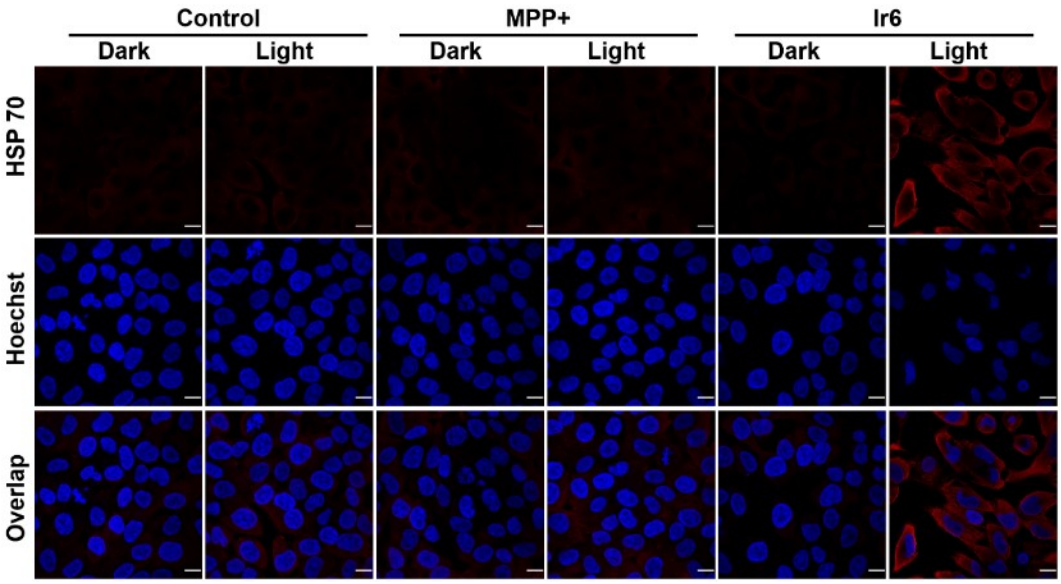

Figure S39. Immunofluorescence CLSM of A375 cells incubated with the 70 kilodaltons heat shock protein-specific antibody and treated with MPP+ or **Ir6** in the dark or upon irradiation (L). L = two-photon laser irradiation at 720 nm, 40 mW, 120 s. Concentration: **Ir6** (10 μM), MPP+ (100 μM). Scale bars = 20 μm.

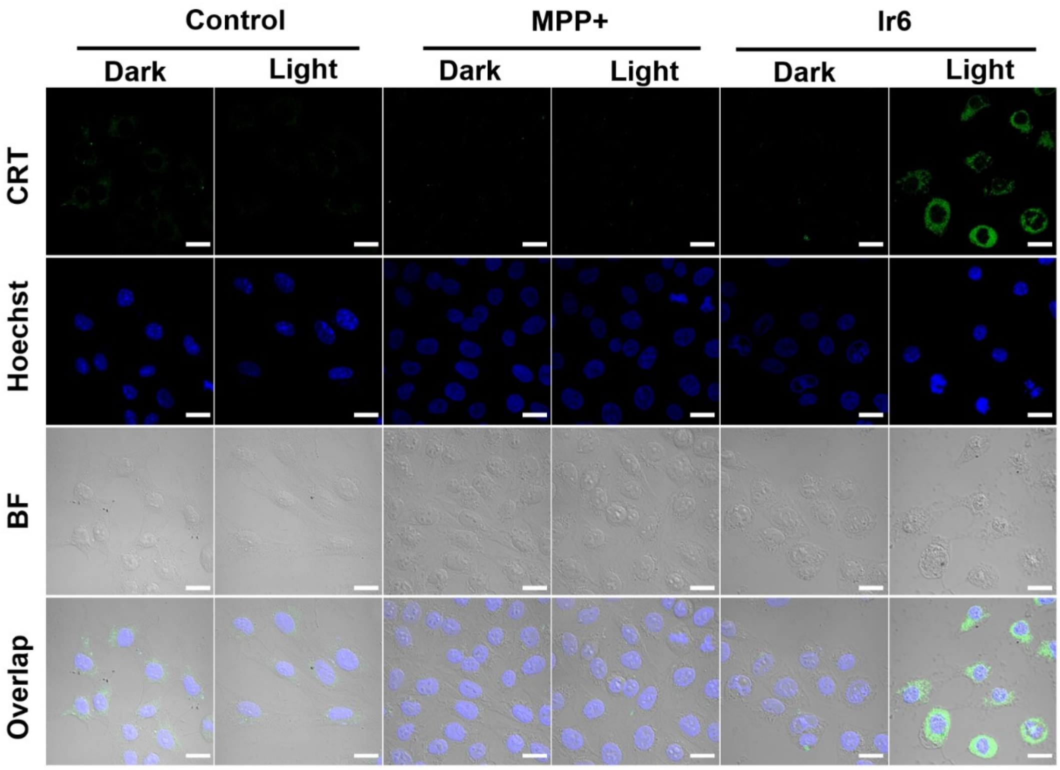

Figure S40. Immunofluorescence CLSM of B16-F10 cells incubated with the calreticulin-specific antibody and treated with MPP+ or **Ir6** in the dark or upon irradiation (Light). Light = two-photon laser irradiation at 720 nm, 40 mW, 120 s. Concentration: **Ir6** (10 μM), MPP+ (100 μM). Scale bar = 20 μm.

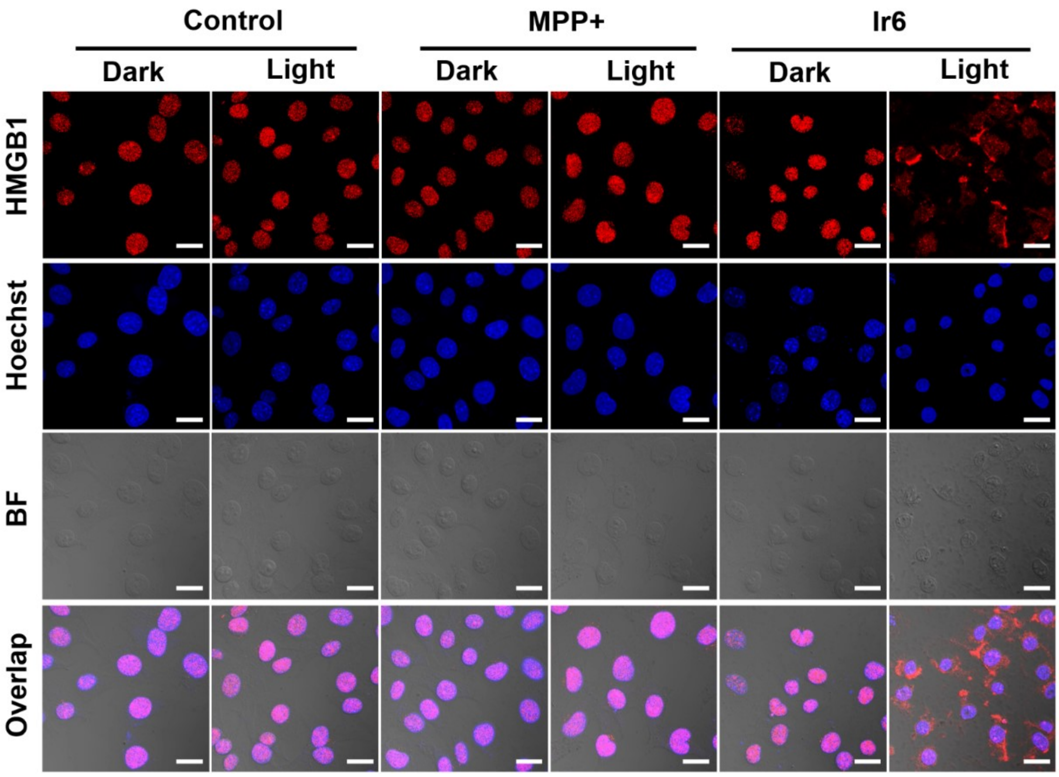

Figure S41. Immunofluorescence CLSM of B16-F10 cells incubated with the human nuclear high mobility group box 1 protein-specific antibody and treated with MPP+ or **Ir6** in the dark or upon irradiation (Light). Light = two-photon laser irradiation at 720 nm, 40 mW, 120 s. Concentration: **Ir6** (10 μM), MPP+ (100 μM). Scale bar = 20 μm.

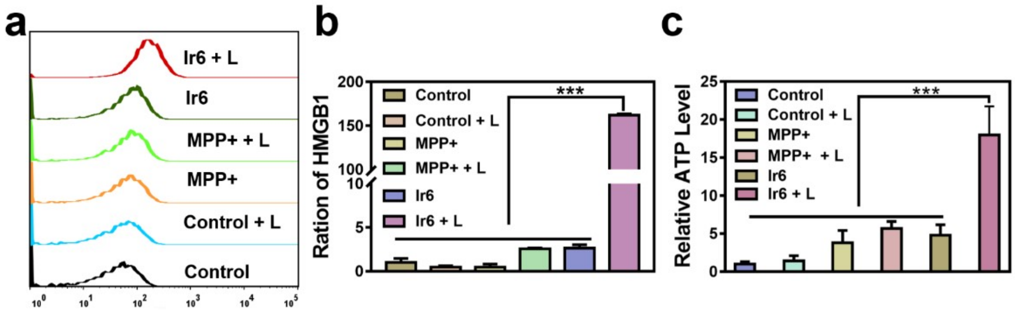

Figure S42. (a) Flow cytometry plots the translocation of Calreticulin in B16-F10 cells upon treatment with **Ir6** in the dark or exposure to irraidation (L). (b) Enzyme-linked immunosorbent assay analysis of B16-F10 cells of the migration of the human nuclear high mobility group box 1 protein upon treatment with **Ir6** in the dark or exposure to irraidation (L). (c) Extracellular release of ATP of B16-F10 cells upon treatment with **Ir6** in the dark or exposure to irraidation (L). L = two-photon laser irradiation at 720 nm, 40 mW, 120 s. Concentration: **Ir6** (10 μM), MPP+ (100 μM). Error bars = standard deviation, n = 3. ***p<0.001.

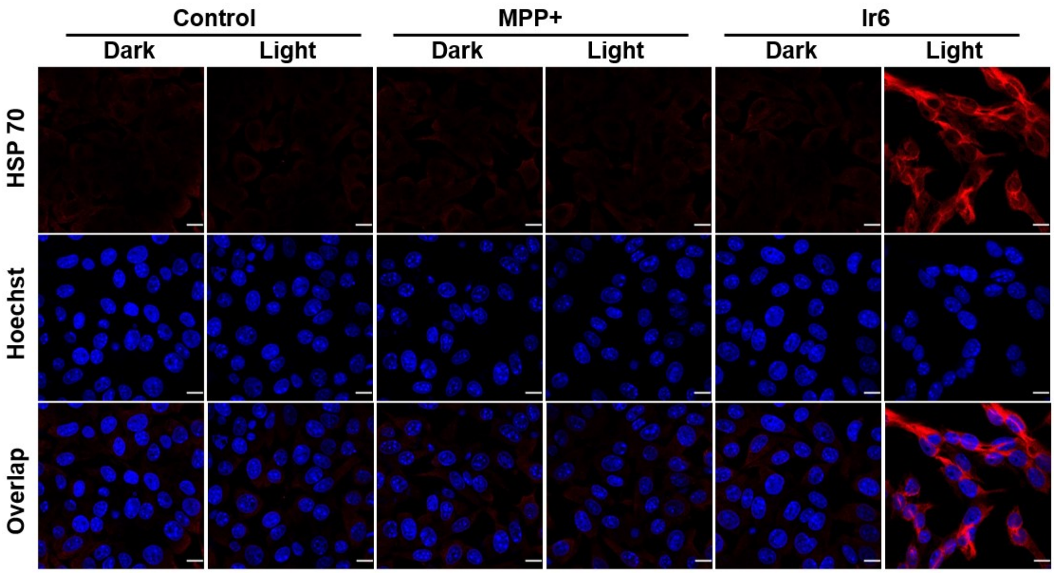

Figure S43. Immunofluorescence CLSM of B16-F10 cells incubated with the 70 kilodaltons heat shock protein-specific antibody and treated with MPP+ or **Ir6** in the dark or upon irradiation (Light). Light = two-photon laser irradiation at 720 nm, 40 mW, 120 s. Concentration: **Ir6** (10 μM), MPP+ (100 μM). Scale bars = 20 μm.

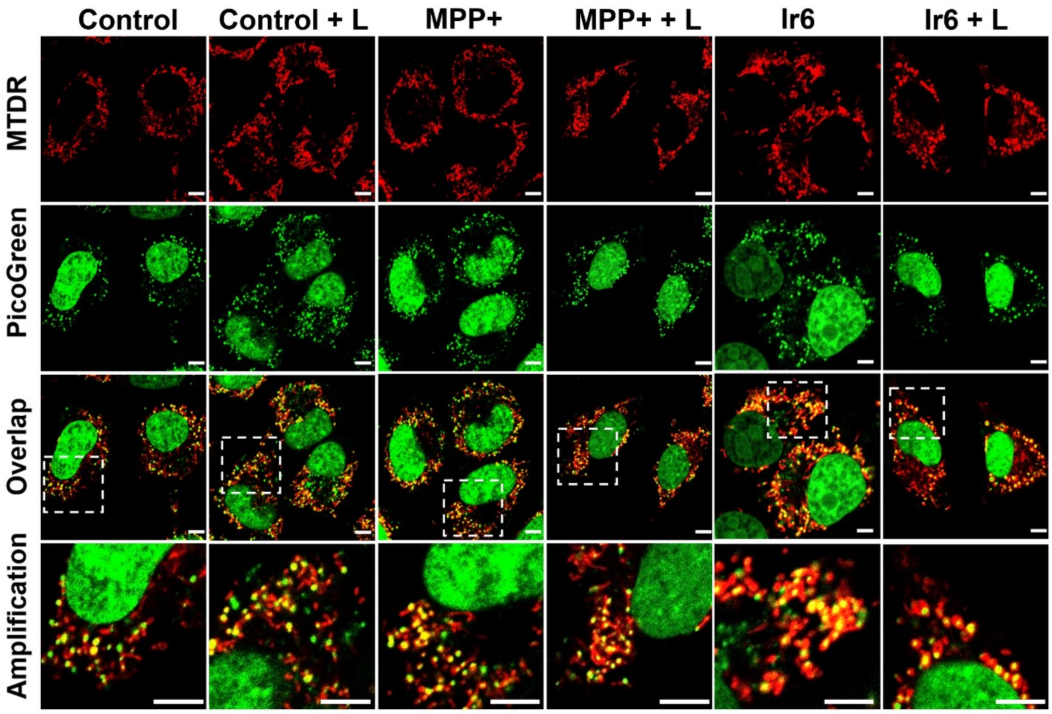

Figure S44. A375 cells were treated with MPP+ or **Ir6** in the dark or upon irradiation (L). L = two-photon laser irradiation at 720 nm, 40 mW, 120 s. Concentration: **Ir6** (10 μM), MPP+ (100 μM). Scale bars = 5 μm.

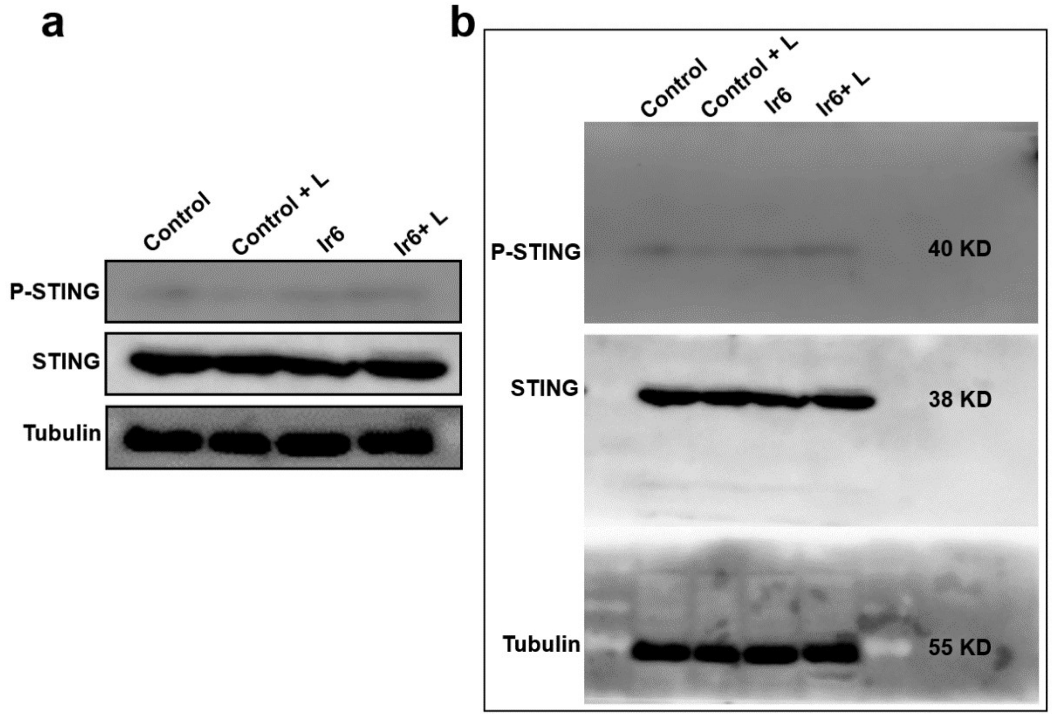

Figure S45. (a) Western Blot analysis of the expression level of P-STING in A375 cells upon treatment with **Ir6** and irradiation (L). (b) The uncropped Western Blot for **Figure S45a**. Concentration: **Ir6** (10 μM). L = two-photon laser irradiation at 720 nm, 40 mW, 120 s.

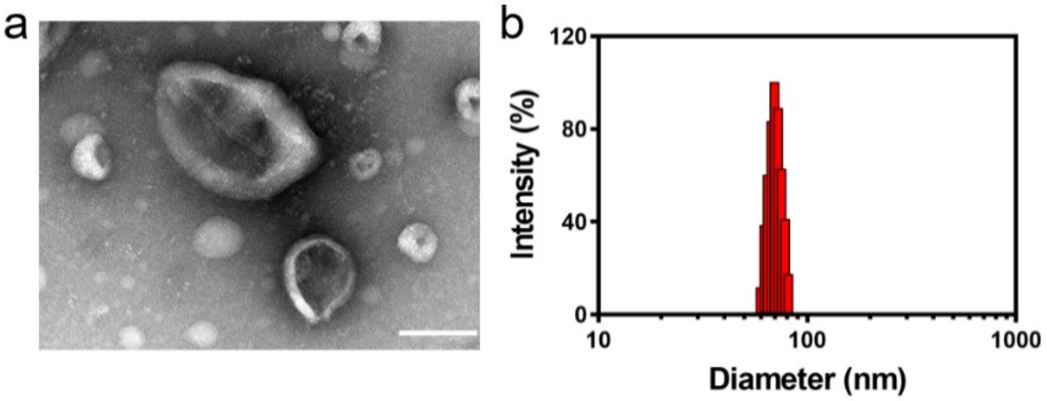

Figure S46. (a) Transmission electron microscopy image and (b) Dynamic light scattering measurement of EXOs. Scale bar = 50 nm.

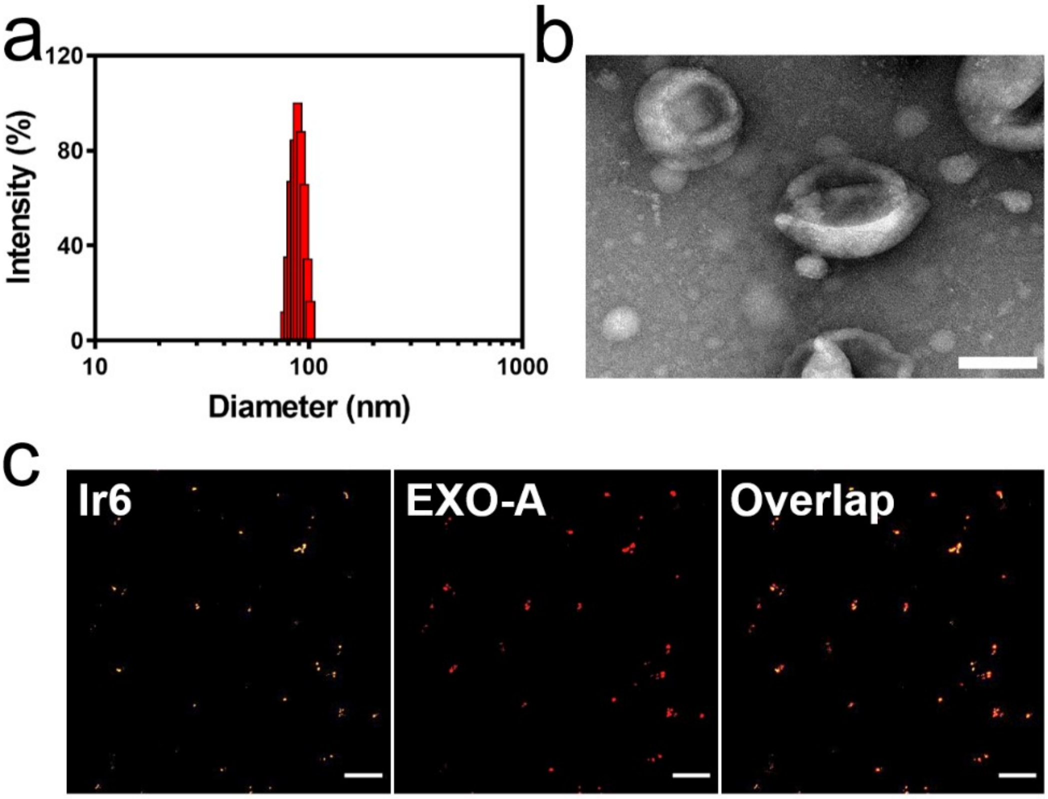

Figure S47. (a) Dynamic light scattering measurement of **Ir6@EXO-A**. (b) Transmission electron microscopy TEM image of **Ir6@EXO-A**. Scale bar = 50 nm. (c) CLMS images upon incubation of **Ir6@EXO-A** with the membrane fluorescent probe 1,1'-dioctadecyl-3,3,3',3'-tetramethylindocarbocyanine perchlorate. Scale bar = 10 µm.

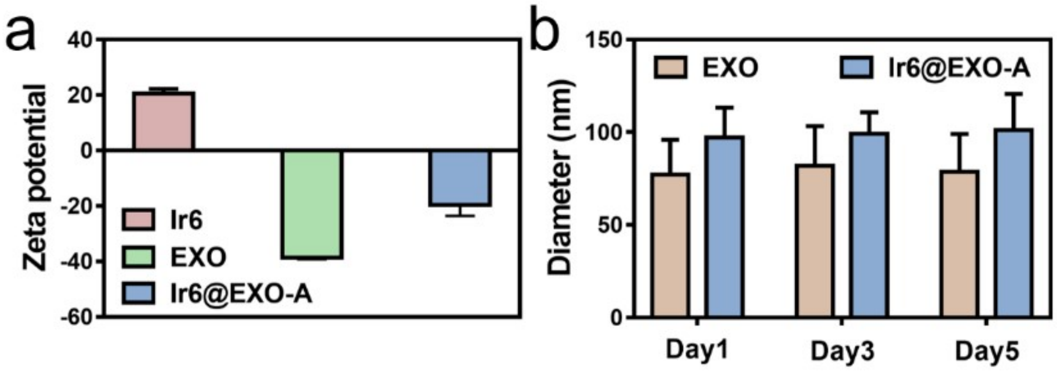

Figure S48. (a) Zeta potential of **Ir6**, EXO, and **Ir6@EXO-A**. (b) Hydrodynamic diameter of EXO and **Ir6@EXO-A** upon incubation in water for various time points, n=3.

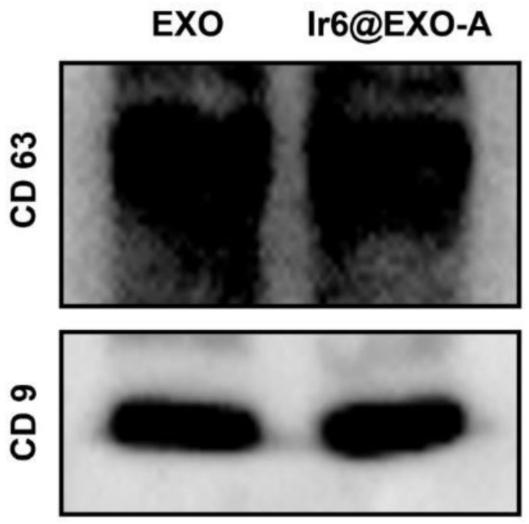

Figure S49. Western Blot analysis of the marker proteins CD63 and CD9 of EXO and **Ir6@EXO-A.**

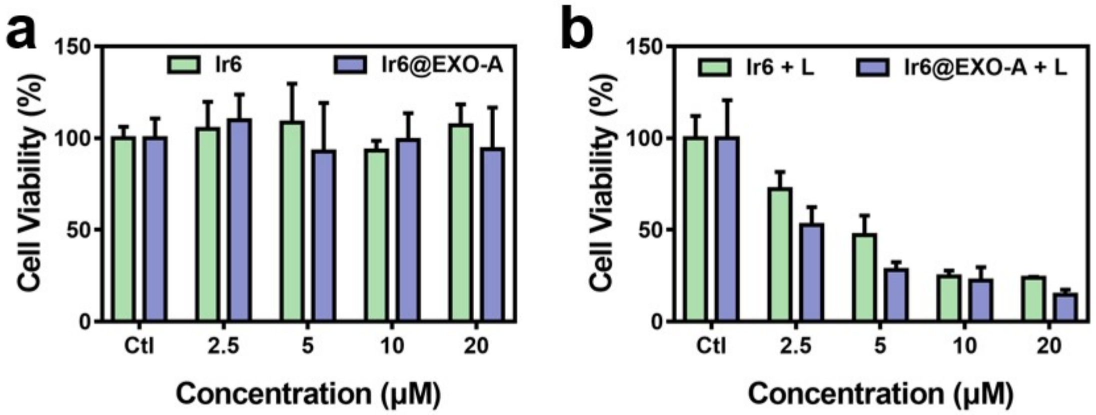

Figure S50. Cytotoxicity of **Ir6** and **Ir6@EXO-A** in the dark or upon two-photon irradiation (720 nm, 40 mW, 120 s).

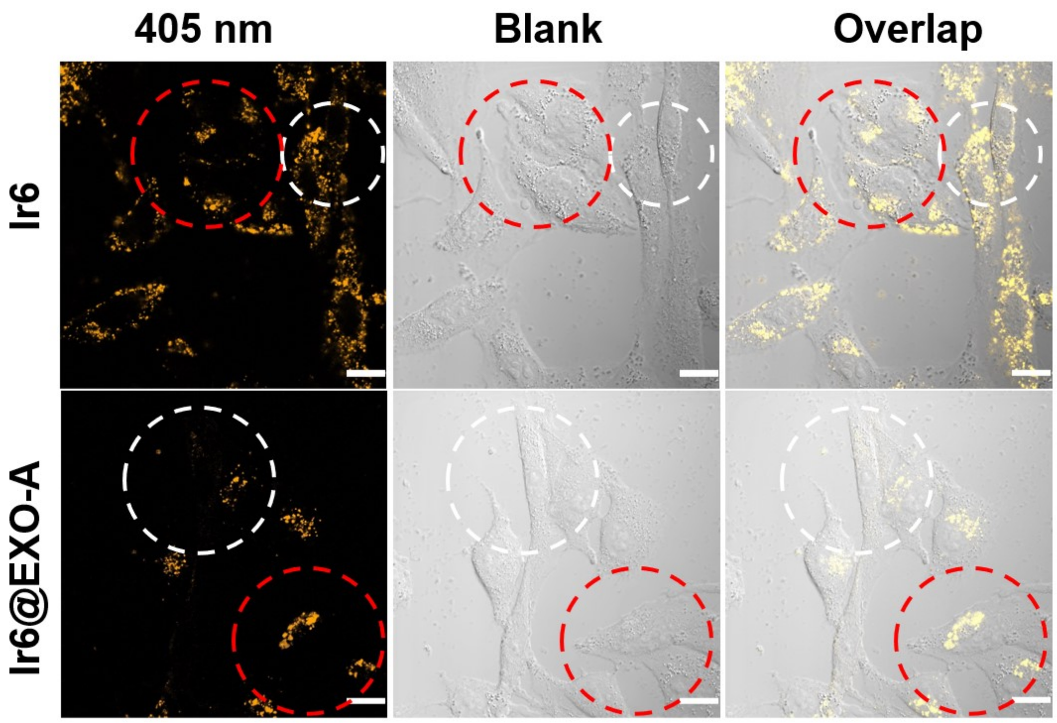

Figure S51. CLMS images in the mixture of HLF and A375 cells incubated with **Ir6** or **Ir6@EXO-A** at 37 °C for 6 h. White cycle: HLF cells, red cycle: A375 cells. Scale bar = 20 µm.

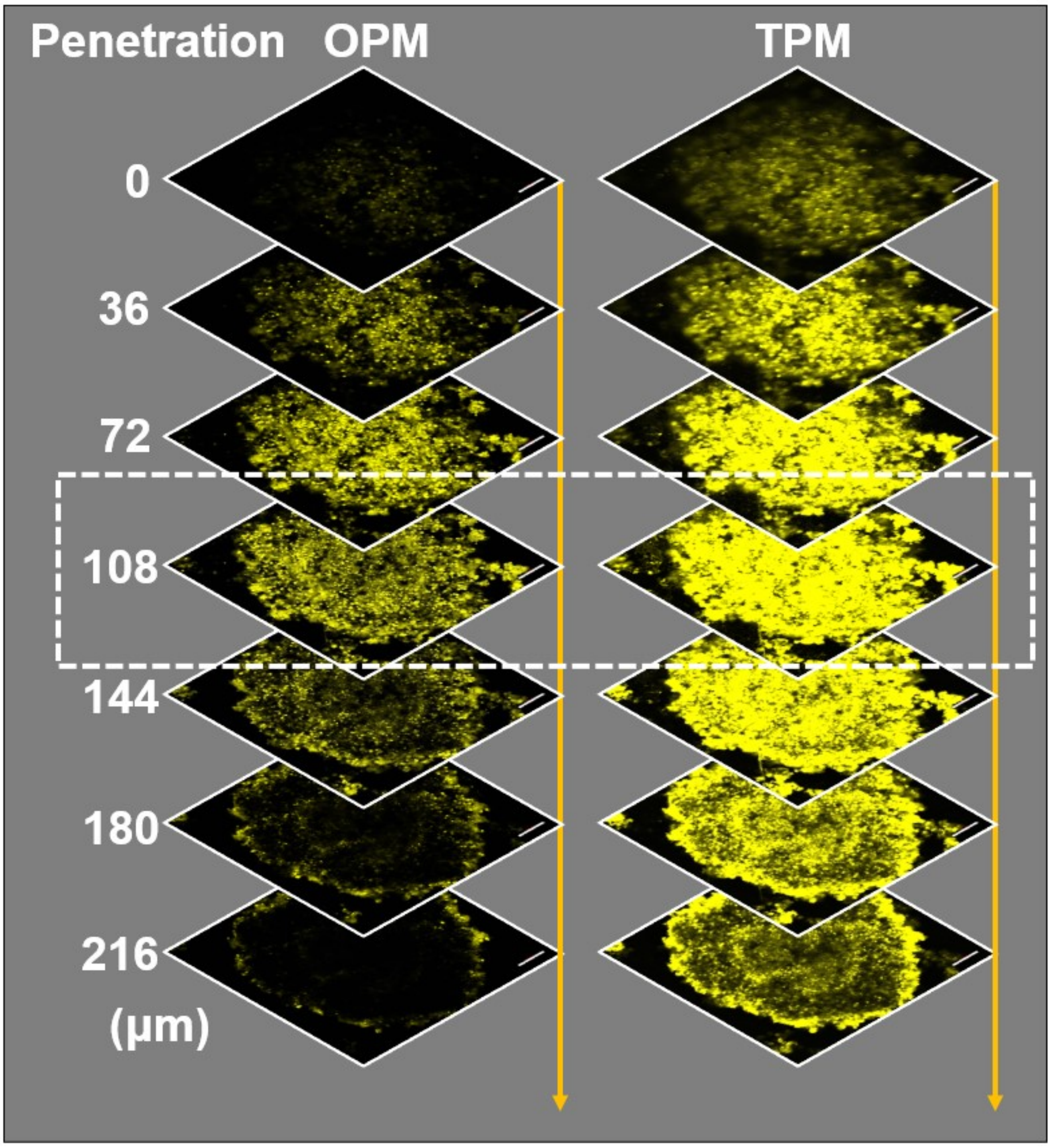

Figure S52. Axially recorded one-photon and two-photon excited CLSM images of **Ir6@EXO-A** in an intact 3D multi-cellular spheroid.

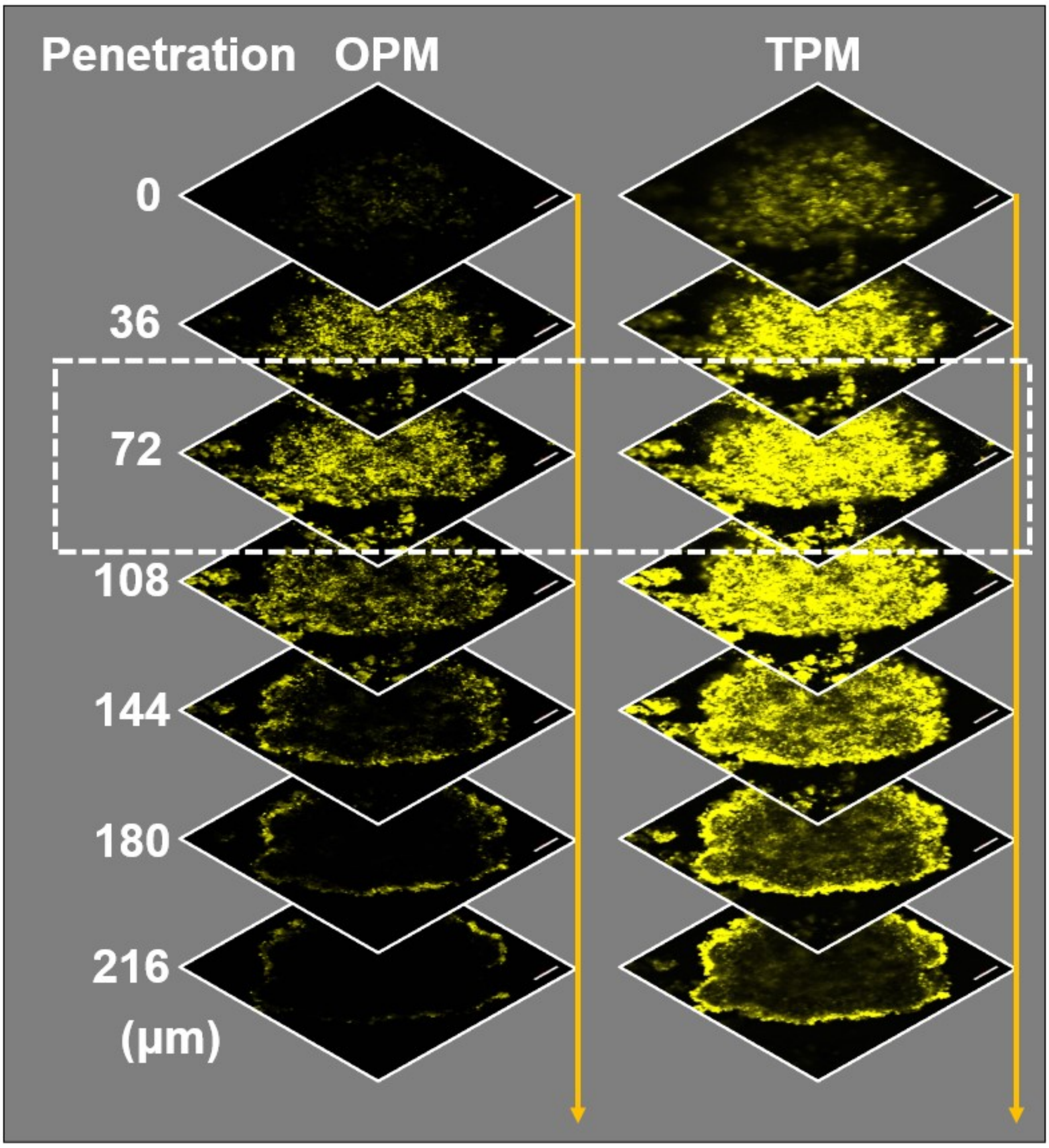

Figure S53. Axially recorded one-photon and two-photon excited CLSM images of **Ir6** in an intact 3D multi-cellular spheroid.

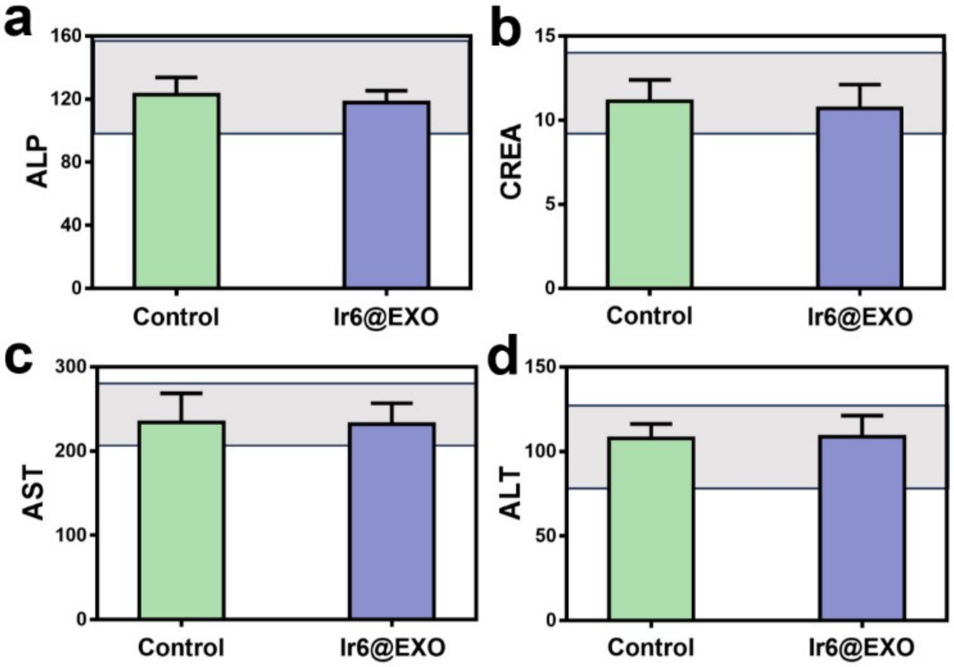

Figure S54. Determination of the biochemical markers, including (a) alkaline phosphatase (ALP), (b) creatinine (CREA), (c) aspartate aminotransferase (AST), (d) alanine aminotransferase (ALT) 7 days after intravenous injection of **Ir6@EXO-A** or control (saline) into healthy BALB/c nude mice. The gray band corresponds to the acceptable normal range. Error bars = standard deviation, n = 3.

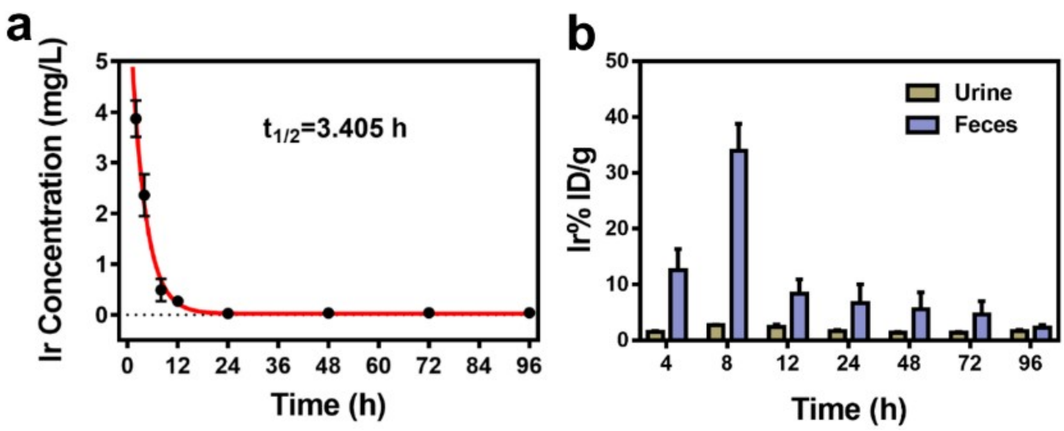

Figure S55. (a) Determination of the blood circulation lifetime upon time-dependent monitoring of the metal concentration in the blood by inductively coupled plasma mass spectrometry after injection of **Ir6@EXO-A** into the tail vein of healthy BALB/c nude mice. (b) Determination of the excretion upon time-dependent monitoring of the metal concentration in the urine and feces by inductively coupled plasma mass spectrometry after injection of **Ir6@EXO-A** into the tail vein of healthy BALB/c nude mice. Error bars = standard deviation, n = 3.

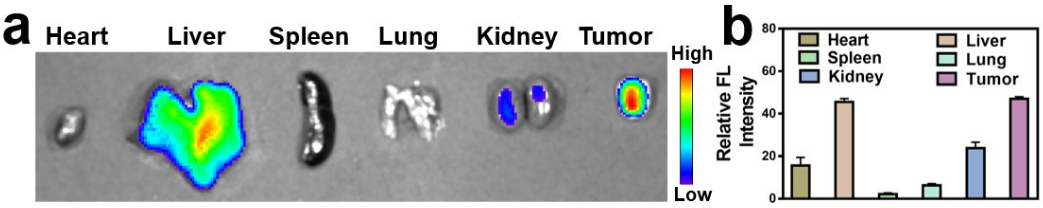

Figure S56. (a) Luminescence photographs of the organs of an A375 tumor-bearing mouse model after injection of **Ir6@EXO-A** into the tail vein in an animal imaging system. (b) Determination of the time-dependent biodistribution from (a). Error bars = standard deviation, n = 3.

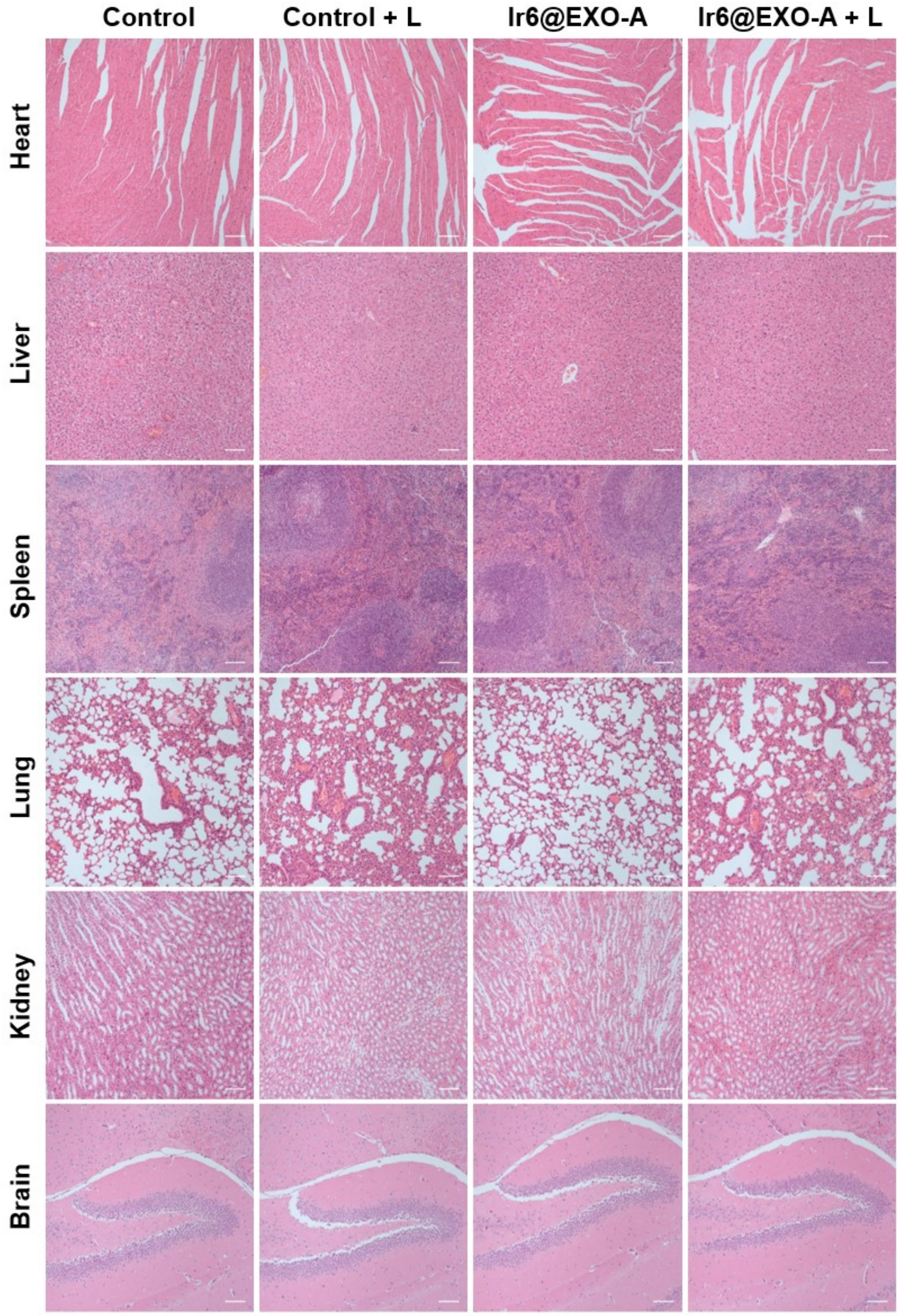

Figure S57. Hematoxylin and eosin stain (H&E) of the major organs (heart, liver, spleen, lung, kidney, and brain) of an A375 tumor-bearing mouse model 16 days after treatment. I: control, II: control + L, III: **Ir6@EXO-A**, IV: **Ir6@EXO-A** + L. L = two-photon laser irradiation at 720 nm, 50 mW, 300 s. Scale bar = 20 µm.

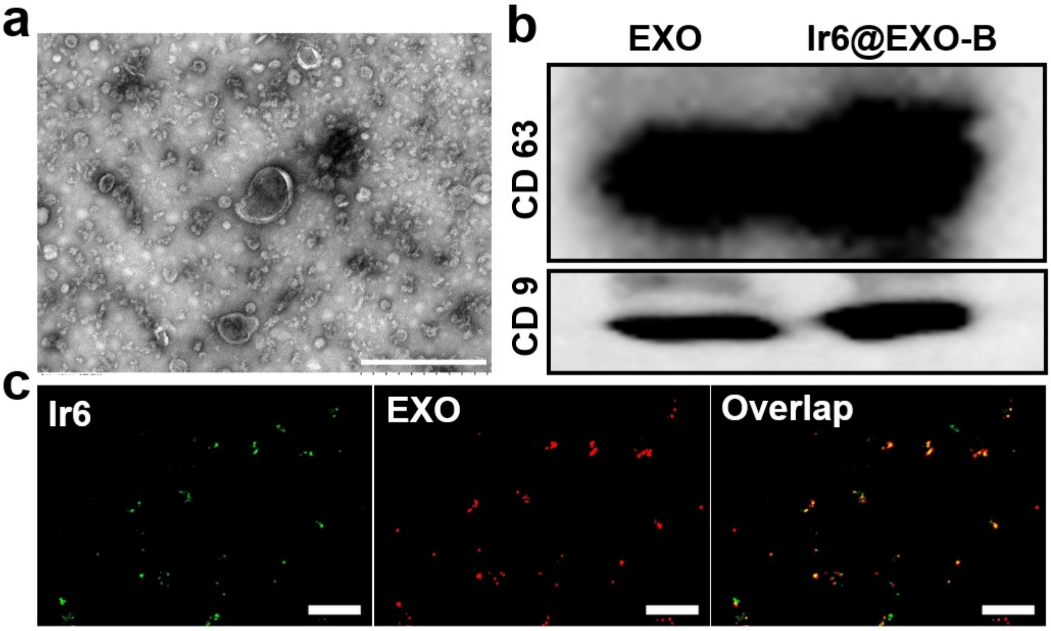

Figure S58. Characterization of **Ir6@EXO-B**. (a) Transmission electron microscopy image of **Ir6@EXO-B**. Scale bar = 500 nm. (b) Western Blot analysis of the marker proteins CD63 and CD9 of EXO and **Ir6@EXO-B**. (c) CLMS images upon incubation of **Ir6@EXO-B** with the membrane fluorescent probe 1,1'-dioctadecyl-3,3,3',3'-tetramethylindocarbocyanine perchlorate. Scale bar = 10 μm.

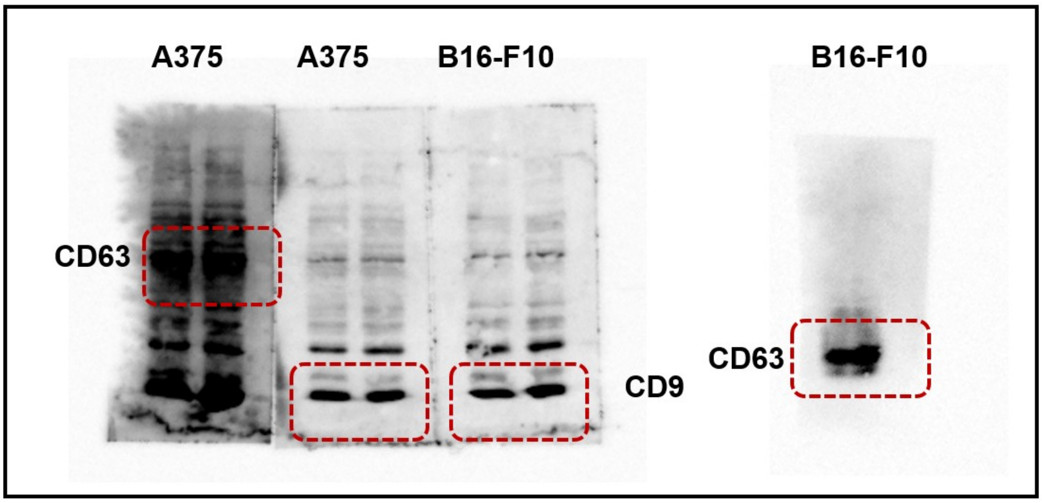

Figure S59. The uncropped Western Blot for **Figure S49** and **Figure S58b.**

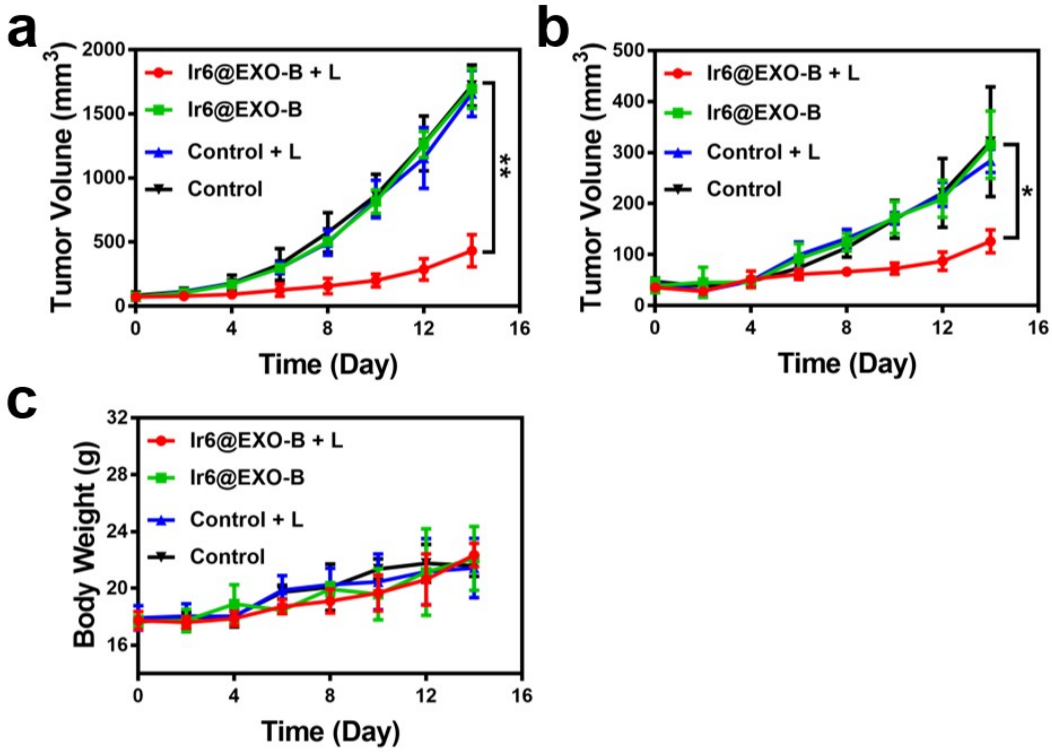

Figure S60. (a) Tumor growth inhibition curves of the primary tumor in a B16-F10 tumor-bearing mouse model during the treatment. (b) Tumor growth inhibition curves of the secondary tumor in a B16-F10 tumor-bearing mouse model during the treatment. (c) Changes in body weight of the animal model. L = two-photon laser irradiation at 720 nm, 50 mW, 300 s. Error bars = standard deviation, n = 5. *p<0.5, **p<0.01.

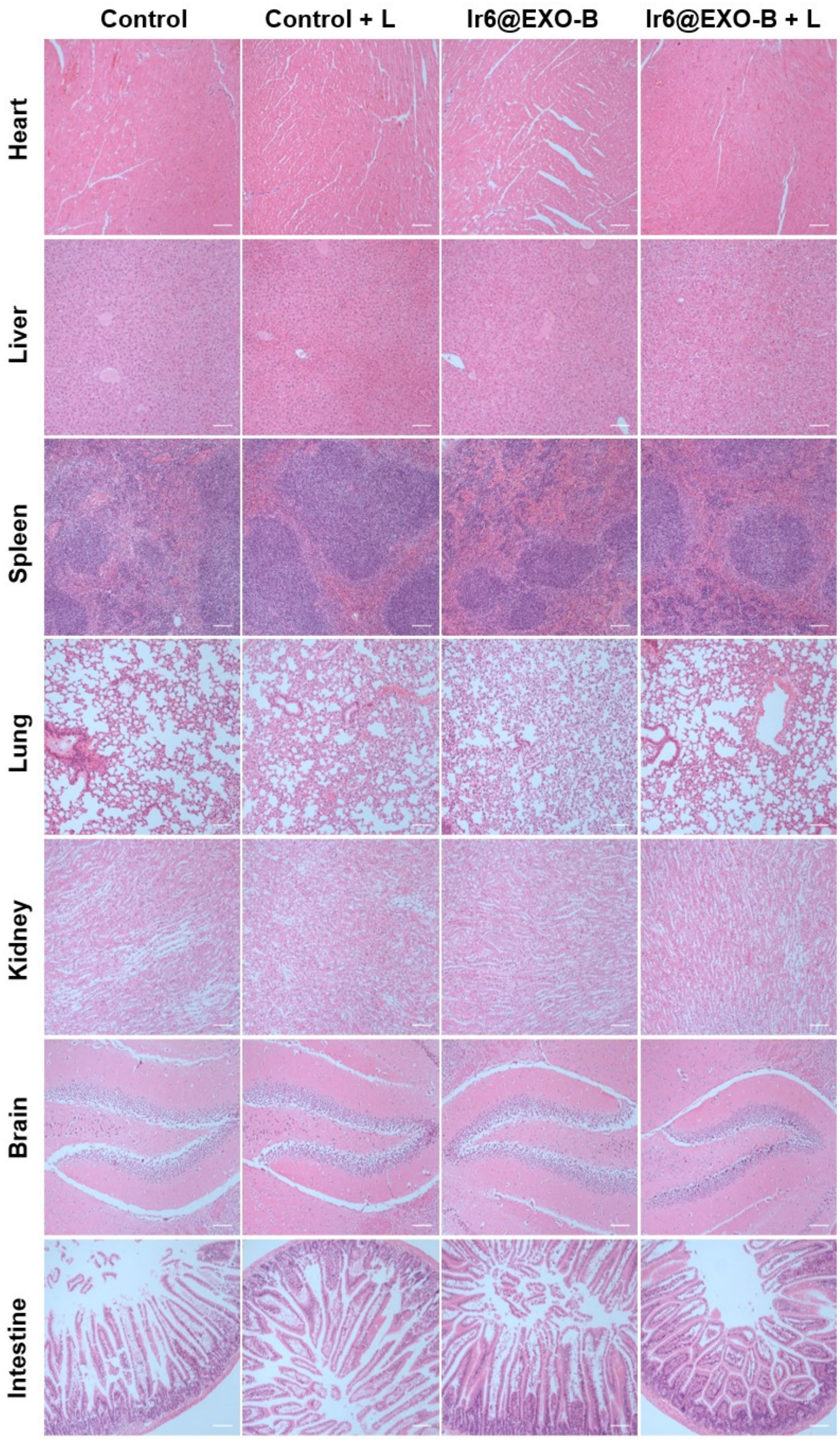

Figure S61. Hematoxylin and eosin stain (H&E) of the major organs of a B16-F10 tumor-bearing mouse model with a primary and secondary tumor 14 days after treatment. I: control, II: control + L, III: **Ir6@EXO-B**, IV: **Ir6@EXO-B** + L. L = two-photon laser irradiation at 720 nm, 50 mW, 300 s. Scale bar = 20 µm.

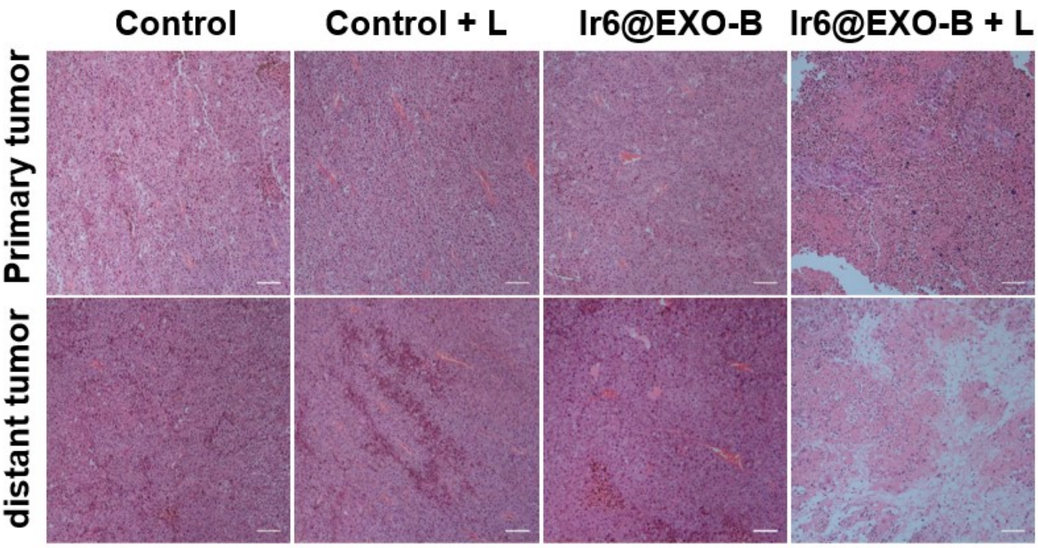

Figure S62. Hematoxylin and eosin stain (H&E) of the tumorous tissue of a B16-F10 tumor-bearing mouse model with a primary and distant tumor 14 days after treatment. I: control, II: control + L, III: **Ir6@EXO-B**, IV: **Ir6@EXO-B** + L. L = two-photon laser irradiation at 720 nm, 50 mW, 300 s. Scale bar = 20 µm.

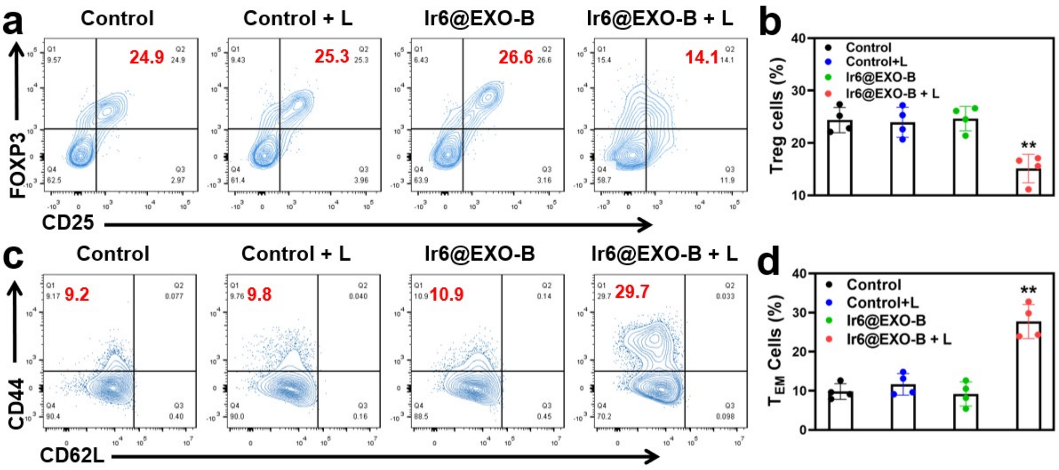

Figure S63. (a) Representative flow cytometry plots and (b) quantitative analysis results of regulatory T cells after gating on CD45+ CD3+ CD4+ T cells in the primary tumor under different treatments. (c) Representative flow cytometry plots and (d) quantitative analysis results of effector memory T cells after gating on CD45+ CD3+ CD8+ T cells in the primary tumor under different treatments. L = two-photon laser irradiation at 720 nm, 50 mW, 300 s. n = 5. **p<0.01.

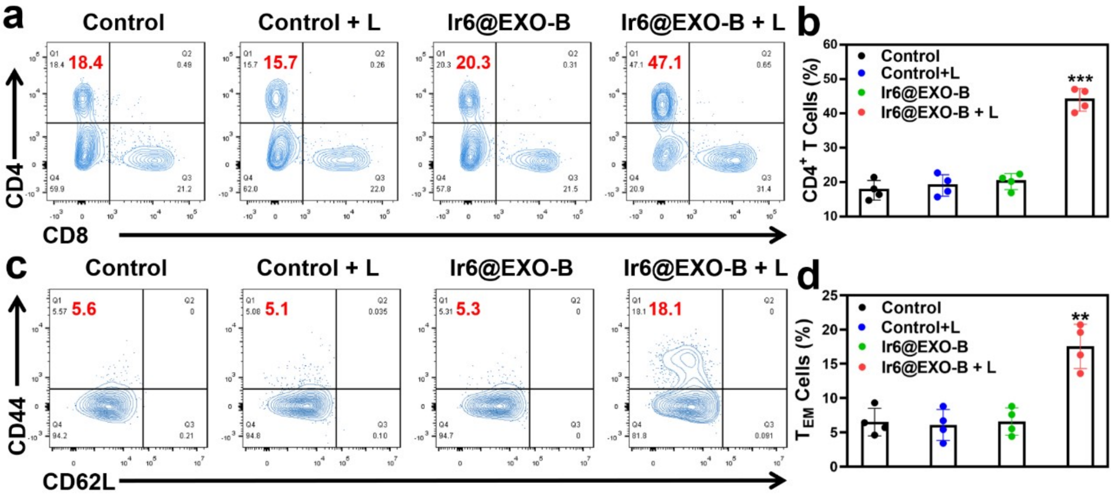

Figure S64. (a) Representative flow cytometry plots and (b) quantitative analysis results of $CD8^+$ and $CD4^+$ T cells after gating on $CD45^+$ $CD3^+$ T cells in the distant tumors under different treatments. (c) Representative flow cytometry plots and (d) quantitative analysis results of effector memory T cells after gating on $CD45^+$ $CD3^+$ $CD8^+$ T cells in the distant tumor under different treatments. L = two-photon laser irradiation at 720 nm, 50 mW, 300 s. n = 5. $^{**}p<0.01$, $^{***}p<0.001$.

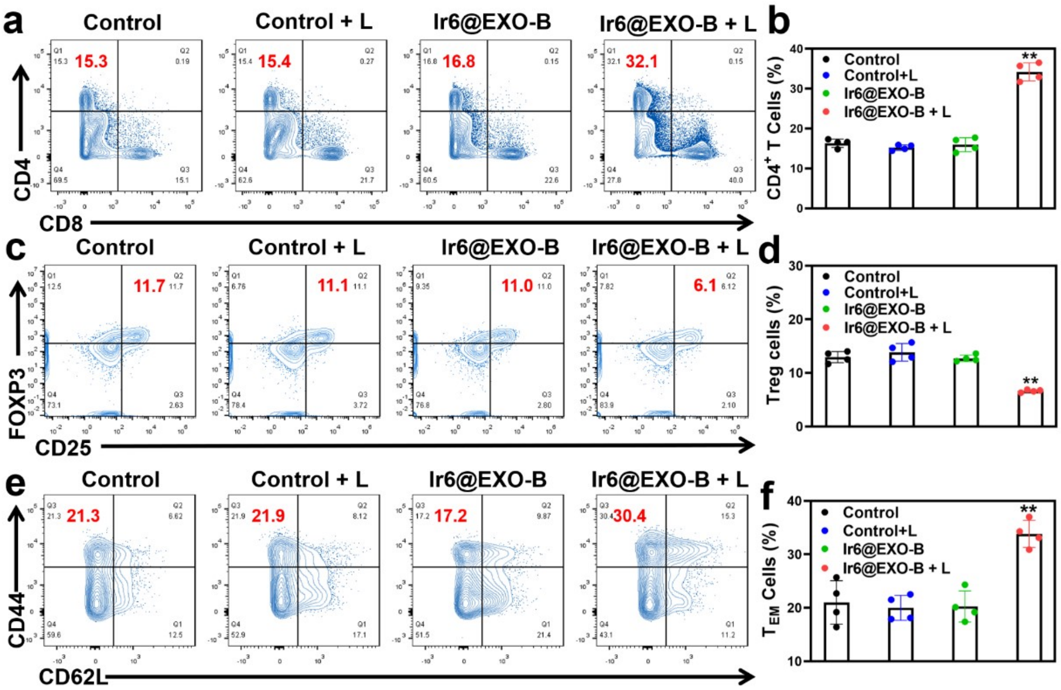

Figure S65. (a) Representative flow cytometry plots and (b) quantitative analysis results of $CD8^+$ and $CD4^+$ T cells after gating on $CD45^+$ $CD3^+$ T cells in the spleen under different treatments. (c) Representative flow cytometry plots and (d) quantitative analysis results of regulatory T cells after gating on $CD45^+$ $CD3^+$ $CD4^+$ T cells in the spleen under different treatments. (e) Representative flow cytometry plots and (f) quantitative analysis results of effector memory T cells after gating on $CD45^+$ $CD3^+$ $CD8^+$ T cells in the spleen under different treatments. L = two-photon laser irradiation at 720 nm, 50 mW, 300 s. n = 5. **$p<0.01$.

Table S1. The purity of **Ir1-Ir6** were assessed by HPLC.

	Peak	R. time	Area	Relative Area (%)
**Ir1**	1	3.610	534627	99.438
	2	5.636	5293	0.562
**Ir2**	1	4.329	9390	3.356
	2	5.702	368557	96.644
**Ir3**	1	4.565	2161	2.531
	2	5.618	83219	97.469
**Ir4**	1	4.048	10804	3.094
	2	5.714	338451	96.096
**Ir5**	1	3.962	11185	3.813
	2	5.743	282167	96.187
**Ir6**	1	5.737	414009	99.351
	2	7.546	2706	0.649

Table S2. $^1O_2$ quantum yields of **Ir1-Ir6**.

**Photosensitizer**	**Slope (-1×10^2)**	**Calculated ϕ_Δ**
Ir1	0.7257	0.300832
Ir2	1.517	0.628858
Ir3	1.739	0.720886
Ir4	1.261	0.522736
Ir5	0.8264	0.342577
Ir6	1.868	0.774362
MB	0.6272	Reference Value 0.52[7]

Table S3. (Photo-) cytotoxicity in µM of MPP+ and **Ir6** in the dark or upon two-photon irradiation (720 nm, 40 mW, 120 s).

**Cell lines**	**MPP+**		**Ir6**		PI(**Ir6**)
	Dark	Light	Dark	Light	
HCT116	>500	>500	85.13 ± 0.21	7.57 ± 0.31	11.25
CT26	>468.41 ± 0.71	>450.73 ± 0.94	75.31 ± 0.32	8.73 ± 0.29	8.63
MDA-MB-231	>500	>500	73.02 ± 0.38	6.52 ± 0.24	11.20
4T1	>500	>500	89.35 ± 0.27	12.75 ± 0.25	7.01
A549	>500	>500	87.79 ± 0.36	9.39 ± 0.31	9.35
LLC	>500	>500	76.45 ± 0.35	8.73 ± 0.33	8.76
A375	>500	>500	88.71 ± 0.24	5.37 ± 0.02	16.52
B16-F10	>500	>500	71.02 ± 0.38	4.26 ± 0.22	16.67
HLF	>500	>500	>100	6.50 ± 0.06	>15.38

Table S4. Routine blood indexes of healthy BALB/c Nude mice after different treatments. The data are presented as mean ± standard deviation (n = 3).

Item	Control	Ir6@EXO-A
WBC (10^9/L)	5.48 ± 0.72	5.12 ± 0.16
RBC (10^12/L)	11.35 ± 0.51	11.01 ± 0.53
HGB (g/L)	182 ± 4.58	176 ± 4.36
HCT (%)	54.36 ± 4.53	51.5 ± 2.01
MCV (fL)	46.25 ± 0.74	46.12 ± 0.76
MCH (pg)	16.15 ± 0.38	15.98 ±0.33
MCHC (g/L)	348.5 ±4.66	346.25 ± 3.2
PLT (10^9/L)	905.5 ± 341.06	1026.25 ± 298.52
RDW-SD (fL)	25.3 ± 2.23	24.68 ± 0.79
RDW-CV (%)	13.9 ± 1.61	13.58 ± 0.4
PDW (fL)	7.13 ± 0.68	8.63 ± 0.16
MPV (fL)	8.1 ± 0.47	8.33 ± 0.13
P-LCR (%)	12.8 ± 4.48	13.75 ± 0.93
PCT (%)	0.53 ± 0.17	0.55 ± 0.22
NRBC (10^9/L)	0.12 ± 0.06	0.1 ±0.06
NRBC (%)	2.08 ± 1.48	1.63 ±0.66
NEUT (10^9/L)	2.43 ± 1.05	2.12 ± 0.66
LYMPH (10^9/L)	3.81 ± 1.59	3.68 ± 1.68
MONO (10^9/L)	0.54 ± 0.41	0.26 ± 0.1
EO (10^9/L)	8.33 ± 0.13	0.25 ± 0.1
NEUT (%)	34.83 ± 3.77	34.35 ± 6.57
LYMPH (%)	55.05 ± 4.47	57.5 ± 5.85
MONO (%)	7.1 ± 1.98	4.23 ± 1.23
EO (%)	2.93 ± 1.08	3.9 ± 0.52
RET (%)	3.74 ± 1.1	3.63 ± 0.22
RET (10^9/L)	438.93 ± 123.83	405.18 ±40.45
IRF (%)	61.63 ± 5.86	55.68 ± 2.42
LFR (%)	38.38 ± 5.86	44.32 ± 2.42
MFR (%)	17.73 ± 1.85	19 ± 1.82
HFR (%)	43.9 ± 7.61	36.68 ± 0.74
RET-He(pg)	17.9 ±0.41	18.15 ±0.13
